# Assessing the association of type 2 diabetes with skin health status: a study of the Northern Finland Birth Cohort 1966

**DOI:** 10.1136/bmjopen-2025-109709

**Published:** 2026-07-10

**Authors:** Maryam Nasserinejad, Laura Huilaja, Suvi-Päivikki Sinikumpu, Seppo Vainio, Juha Röning, Nsrein Ali, Sylvain Sebert

**Affiliations:** 1 Research Unit of Population Health, Faculty of Medicine, University of Oulu, Oulu, Finland; 2 Department of Dermatology, Oulu University Hospital, Research Unit of Clinical Medicine, Medical Research Center, University of Oulu, Oulu, Northern Ostrobothnia, Finland; 3 Faculty of Biochemistry and Molecular Medicine, Disease Networks, Laboratory of Developmental Biology, University of Oulu, Oulu, Finland; 4 Biomimetics and Intelligent Systems Group, Faculty of Information Technology and Electrical Engineering, University of Oulu, Oulu, Finland; 5 Symbiosis Institute of Technology, Symbiosis International (Deemed University), Pune, India; 6 Research Unit of Health Sciences and Technology, Faculty of Medicine, University of Oulu, Oulu, Finland

**Keywords:** Dermatology, Public health, General diabetes

## Abstract

**Objectives:**

There is evidence to suggest that alterations in skin health often co-occur with type 2 diabetes (T2D). However, the underlying mechanisms remain unclear. Despite their clinical relevance, skin conditions are often underdiagnosed in diabetes care. This study applied variable selection methods to identify key dermatological factors linked to T2D and examined their direct and indirect associations.

**Design:**

Prospective birth cohort study.

**Participants:**

This study analysed data from the Northern Finland Birth Cohort 1966, including 1906 participants (1024 women, 882 men) at ages 31 and 46 years. At age 46 years, the cohort participants, living in the region of Northern Finland, were invited to a general health clinical examination including the assessment of their T2D status and a dermatological examination.

**Methods:**

Prior to performing a path analysis, we developed a data-driven procedure to select 47 possible skin-related variables for the model. The variable selection combined test-based methods (ie, a univariate analysis and a backward, forward and stepwise logistic regression) and penalty-based/machine learning methods (ie, Least Absolute Shrinkage and Selection Operator, Ridge, minimax convex penalty and Sparse Stepwise Regression). The selected skin-related variables were then included in a path analysis accounting for sex, body mass index (BMI), diet, education, depression, anxiety and sleep quality.

**Results:**

Eight skin-related variables were selected and included in the path analysis. Psoriasis (OR 1.105, 95% CI 1.058 to 1.156) and Tinea pedis (OR 1.067, 95% CI 1.04 to 1.096) showed direct associations with T2D. Hyperhidrosis (OR 1.017, 95% CI 1.005 to 1.037) exhibited an indirect effect.

**Conclusions:**

These results underscored the interconnectedness of dermatological conditions such as psoriasis and Tinea pedis with T2D. These findings suggest that certain skin conditions may serve as potential indicators of T2D. Early recognition and appropriate management of these conditions may help improve patients’ quality of life.

**STRENGTHS AND LIMITATIONS OF THIS STUDY**

- Dermatological examinations were diagnosed by dermatologists, enhancing the accuracy and validity of the data.
- Use of path analyses to examine direct and indirect associations while adjusting for key confounders.
- Applied multiple statistical and machine learning methods for variable selection to improve reliability.
- A community-based study with limited sample size may introduce selection factors and restrict generalisability to the broader Finnish population.
- Skin-related data were collected at a single time point, preventing a longitudinal assessment and causal inference.

## Introduction

Type 2 diabetes (T2D) is one main segment of chronic non-communicable diseases characterised by elevated levels of blood glucose and is associated with serious complications to the heart, blood vessels, digestive system, eyes, kidneys, nerves, skin, and even psychosocial aspects and early mortality.^1^

An estimated 537 million adults aged 20–79 years worldwide (10.5% of all adults in this age group) had diabetes in 2021. By 2030, this number is projected to rise to 643 million, and by 2045, it could reach 783 million. This would represent a 46% increase in the number of people living with diabetes, while the global population is estimated to grow by only 20% over the same period.^2^ According to the WHO, in the European Region alone, approximately 64 million of adults and around 300 000 children and adolescents are living with diabetes as of 2024, making it one of the most common chronic conditions in Europe.^3^ According to the latest reports, among the European Nordic countries, Finland has the highest prevalence of diabetes, at 9.7%. The number of adults with diabetes is estimated to be 392.9 thousand (95% CI 334.8 to 435.9).^2^

The burden of and the premature mortality attributed to T2D are very high, and most people living with T2D are reported to suffer other complications leading to a dramatic reduction in their quality of life.^4^ The comorbidity of skin disease and T2D is undeniable, and almost one third of people with diabetes have skin lesions.^5^

Numerous studies have shown associations between different types of skin disorders with T2D. For example, substantial evidence supports a link between inflammatory diseases, such as psoriasis and eczema, and T2D.^6 7^ In addition, Azfar *et al.* found that the risk of developing T2D increased with psoriasis severity.^8^ Other skin conditions associated with T2D, such as acanthosis nigricans, diabetic dermopathy, necrobiosis lipoidica, bacterial and fungal infections, and xerosis (dry skin), often appear early, sometimes before T2D is diagnosed. These skin disorders may not only reflect the systemic effects of diabetes but can also serve as early indicators, emphasising the importance of timely recognition and management to improve the prevention and/or reduce the burden of T2D.^9 10^

It is currently hypothesised that a large number of skin-related conditions may be directly associated with T2D either through causal mechanisms or psychosocial pathways. This study was designed to identify the most relevant dermatological conditions associated with T2D in the general population living in Northern Finland to examine possible direct and indirect associations between skin health and T2D, while adjusting for potential confounders and covariates such as diet, education, body mass index (BMI), anxiety, depression, sleep quality and sex.

### Materials and methods

### Overview and data resources

#### The selected population

**Figure 1:**
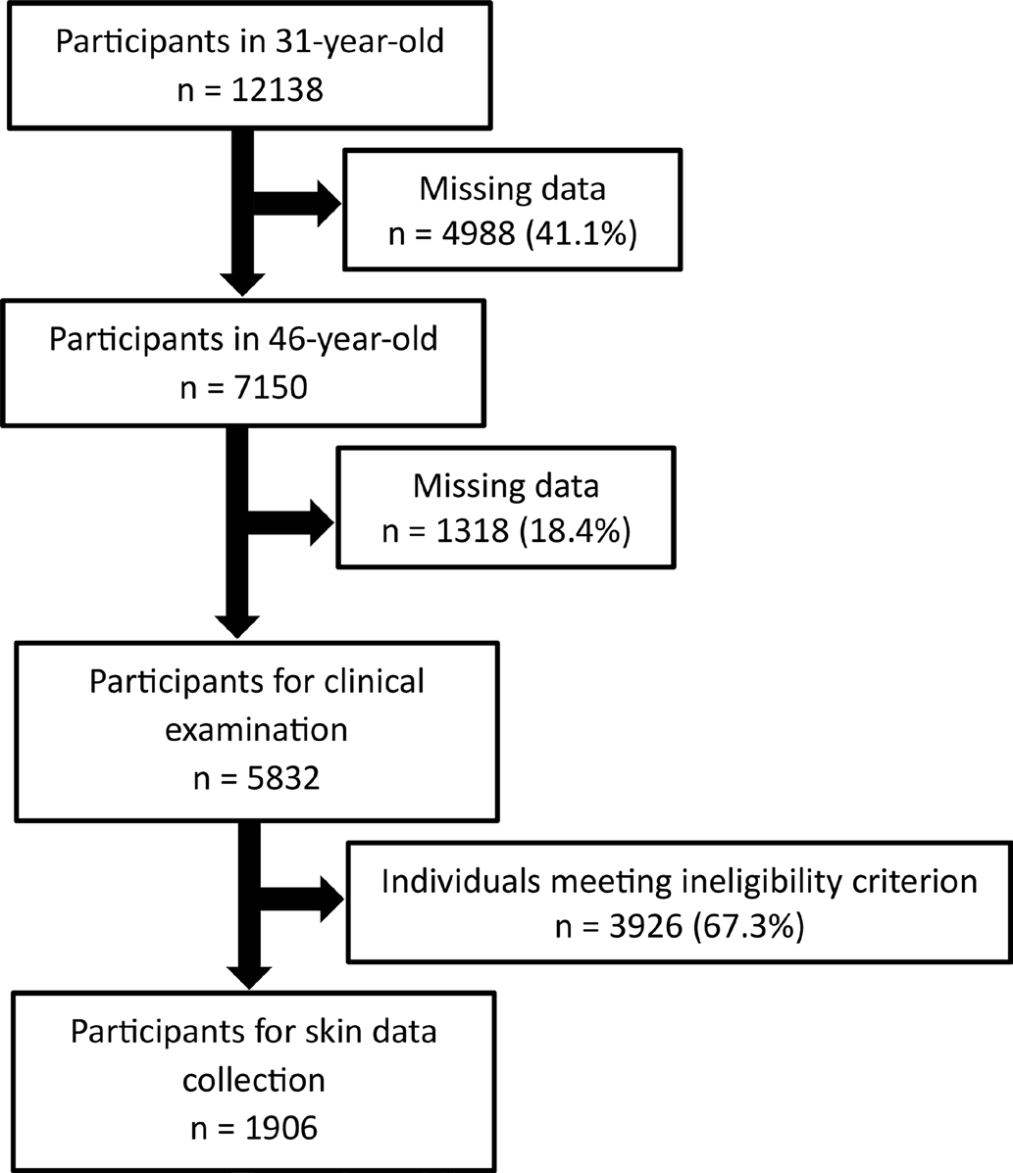
Flow chart demonstrating the process used to select the study participants.

The current study was derived from a longitudinal birth cohort study and used data from the participants of the Northern Finland Birth Cohort 1966 (NFBC1966).^11^ The NFBC1966 is a longitudinal population-based study initiated to investigate factors affecting health and development from prenatal stages into adulthood. The cohort comprised 12 138 live births in 1966, representing 96.3% of all births that year in Finland’s two northernmost provinces, Oulu and Lapland.^12^ The participant selection process from the eligible population is shown in the study flow chart (figure 1). The study included all eligible participants with available follow-up data on their skin health status and T2D defined at age 46 years (in 2012) and all available covariates. Due to logistic and financial constraints, the clinical examination for dermatology diagnosis was only performed for participants living in the region of Oulu and was conducted at the Oulu University Hospital. It included a total of 882 males and 1024 females (N=1906). Multiple imputation by chained equations was used to handle missing data. This approach imputes missing values iteratively by specifying a regression model for each variable with missing data, conditional on the other variables in the dataset. The number of missing values for each variable is presented in online supplemental table S1.

10.1136/bmjopen-2025-109709.supp2 Supplementary data

### Patient and public involvement

Neither patient groups nor participants in the study were involved in the design, conduct, reporting or dissemination plans of this research.

### Exposure variables: skin health status

Each individual's skin health status was assessed using 46 different variables (online supplemental table S1). These variables, also referred to as skin health measures, were included in a comprehensive dermatological assessment protocol designed by dermatologists to assess the prevalence of major skin diseases and disorders in midlife at 46 years of age.^13^ All variables were obtained through clinical examinations, except for doctor-diagnosed psoriasis, which was collected via a postal questionnaire, and two sunscreen-use variables, which were self-reported by the participants.

### Outcome variable: T2D

All T2D-related data were obtained from the 46-year follow-up of the NFBC1966 cohort. We defined cases with T2D according to the following criteria depending on their level of participation or prior status for T2D. All eligible participants were invited to an oral glucose tolerance test (OGTT) with a 75 g anhydrous glucose ingestion. From this test, we classified individuals with T2D (N cases=267) if the fasting OGTT value was 7 mmol/L or higher, and the value at 2 hour-post OGTT was 11.1 mmol/L or greater. Additionally, cases with T2D (N cases=192) were included according to self-reports with the following question: ‘Do you currently have or have you ever had adult-onset diabetes (type 2)?’ Finally, we included additional cases should they report the use of blood sugar-lowering medications like metformin (N cases=102). Some participants met more than one of the above criteria, resulting in overlap between the groups. The total number of unique individuals with T2D was 336.

This comprehensive methodology ensured a robust identification process for individuals with T2D, incorporating both clinical measurements and self-reported information.

### Covariate variables

To account for potential confounding, the model included covariates measured at two different follow-ups. Education, anxiety, depression and diet were used to capture long-term influences from the 31-year follow-up. In contrast, BMI and sleep quality were included as more proximal and time-sensitive factors from the 46-year follow-up to better reflect their temporal proximity to the outcome.

Diet was categorised as a binary variable (healthy vs unhealthy) based on consumption patterns of vegetables, fruits, rye and processed foods.^14^ Education was defined by a combination of two different questions concerning basic education and vocational qualifications with three levels from basic educated to highly educated.

For defining anxiety and depression, we followed the Hopkins symptom checklist.^15^ This questionnaire consists of 14 questions aimed at diagnosing anxiety, focusing on symptoms such as pain, tension, restlessness, trembling, fear and heart pounding. Additionally, there are 13 questions designed to assess depression, covering issues such as difficulty falling asleep, feelings of loneliness and worthlessness, sexual difficulties, lack of energy and suicidal thoughts. The questions were presented in a four-point Likert format, with one being ‘not at all’ and four being ‘very much’. The depression and anxiety scores were derived from the mean scores, which ranged slightly from 1 to 4, where 1 indicated no anxiety or depression and 4 indicated a high level of anxiety or depression.

Information on sleep quality was collected by questionnaire at age 46 years.

To assess potential selection bias arising from participant attrition between the two follow-ups, we compared the baseline covariate distributions at the 31-year follow-up between participants who attended the 46-year follow-up and those who did not; the results are presented in online supplemental table S2.

10.1136/bmjopen-2025-109709.supp3 Supplementary data

### Statistical methods

Descriptive statistics, including counts and percentages for all variables, were computed overall and stratified by sex (male and female) and T2D status (healthy and T2D groups). χ^2^ tests were used to compare categorical variables between sex groups and between health status groups as presented. In addition, covariates were compared over time, and McNemar’s test and paired t-tests were used (online supplemental table S3). Initially, we applied an exploratory factor analysis to reduce the dimensions of the skin status variables, aiming to derive components from the pool of 47 skin health status. However, due to the Kaiser-Meyer-Olkin index of 0.62 and a low cumulative variance (0.275 out of 1.00) for just the first 22 components, we opted against using the outcomes of this model. We opted for an alternative approach to reducing the dimensionality through variable selection.

10.1136/bmjopen-2025-109709.supp4 Supplementary data

### Variable selection

Variable selection is a crucial step in inferential modelling, as accurately identifying the subset of variables truly associated with the outcome of interest is essential. General approaches to variable selection fall into three main categories: test-based, penalty-based and screening-based methods.^16^ While various techniques for variable selection exist, each has distinct mathematical properties, which can lead to differing outcomes in terms of the variables selected. This raises an important challenge for researchers: determining how the choice of selection method influences the study results and their reproducibility across different methods. Investigating the ‘between-method’ variability in variable selection is, therefore, an important area of study.

Best practices suggest employing multiple methods and assigning confidence to variables based on their consistency across methods. In this study, we employed a variety of methods to identify the most appropriate variables for path analysis modelling, including a univariate logistic regression analysis based on p values, as well as a backward stepwise logistic regression (BSLR), forward stepwise logistic regression (FSLR) and stepwise logistic regression (SLR). Additionally, we used machine learning techniques, specifically Least Absolute Shrinkage and Selection Operator regression (Lasso), Ridge regression, minimax convex penalty (MCP) and Sparse Stepwise Regression. The degree of agreement between the results of the various methods was assessed using Cohen’s Kappa index, which ranges from −1 to 1. Values less than 0 indicate poor agreement (worse than chance). A Kappa value below 0 indicates poor agreement (worse than chance), while values between 0.61 and 0.80 suggest substantial agreement, and values above 0.80 indicate almost perfect agreement.^17^ The multicollinearity of the selected variables was assessed using the variance inflation factor (VIF), with a maximum VIF exceeding 10 indicating potential influence of multicollinearity on the least squares estimates.^18^

### Path analysis

Following the selection of skin health status, we employed a path analysis to examine both direct and indirect associations, while controlling for the effects of sex, BMI, diet, education, depression and anxiety (online supplemental figure S1).

10.1136/bmjopen-2025-109709.supp1 Supplementary data

In this study, variables were standardised to facilitate comparison and to eliminate variation arising from differences in scale. The standardisation transformed the original variables so they would have a mean of 0 and an SD of 1, making comparisons between variables with different scales or units more straightforward and for model estimation, the weighted least squares mean and variance adjusted method was used.

The model fit was evaluated using the root mean square error of approximation (RMSEA), the standardised root mean square residual (SRMR) and the relative χ^2^ (χ² minimum discrepancy (CMIN)/df). Values of RMSEA and SRMR below 0.08 and CMIN/df below 5 were considered indicative of an acceptable model fit, based on established recommendations in the structural equation modelling literature.^19^

All analyses were conducted using R software, V.4.5.2, specifically the glmnet package for Lasso and Ridge regression, ncvreg package for MCP method, sparsestep^20^ for sparse stepwise method and the lavaan package for path analysis.

## Results

Among the study participants, the overall prevalence of T2D was 4.8%, with a significantly higher rate observed in men (5.6%) compared with women (4.1%). The prevalence of tinea pedis was 27%, with a higher prevalence in men than in women. Specifically, among patients with T2D, the prevalence of tinea pedis increased to 44.4%, with 55.6% in men and 35.2% in women (table 1). Psoriasis was reported by 2.5% of the participants overall, with a higher prevalence in patients with T2D at 7.1%. In contrast with tinea pedis, this prevalence was higher in women (7.4%) compared with men (6.8%). Rosacea affected 15.2% of the participants, and among those with T2D, the prevalence rose to 23.2%, showing a significant gender disparity with 8.9% in men and 35.2% in women (table 1). Onychomycosis was observed in 9.4% of the participants. Café-au-lait spots were found in 12.5% of the overall population, but only in 3.0% of those with T2D. This was with a notable gender difference: 5.6% in women and no cases reported in men with T2D. Hyperhidrosis was reported in 2.2% of the participants. Lentigo senilis had a prevalence of 13.4%, while Pityriasis versicolor was observed in 1.9% of the participants. Among those with T2D, the prevalence of Pityriasis versicolor was 5.1%, with a higher prevalence in men (6.7%) compared with women (3.7%) (table 1).

**Table 1:** Descriptive characteristics of the study population

Variables	Category	Overall	Men	Women	Healthy	Type 2 diabetes	Type 2 diabetes	
							Men	Women
Sex	Male	3027 (45.5%)	–	–	3027 (45.5%)	180 (53.6%)*	–	–
	Female	3633 (54.5%)	–	–	3633 (54.5%)	156 (46.4%)	–	–
BMI (kg/m^2^)	Underweight	37 (0.6%)	7 (0.3%)	30 (0.9%)*	37 (0.7%)	0 (0.0%)*	0 (0.0%)	0 (0.0%)
	Normal weight	2188 (37.6%)	735 (28.7%)	1453 (44.6%)	2137 (39.3%)	25 (8.8%)	8 (32%)	17 (68%)
	Overweight	3594 (61.8%)	1819 (71.0%)	1775 (54.5%)	3262 (60.0%)	259 (91.2%)	148 (57%)	111 (0.0%)
Diet	Unhealthy	5524 (88.8%)	2317 (83.4%)	3207 (93.2%)*	5208 (89.2%)	234 (83.0%)*	104 (72.2%)	130 (94.2%)
	Healthy	695 (11.2%)	461 (16.6%)	234 (6.8%)	630 (10.8%)	48 (17.0%)	40 (27.8%)	8 (5.8%)
Sleep quality	Satisfactory	3810 (57.2%)	1730 (57.3%)	2080 (57.1%)	3624 (57.3%)	173 (55.1%)	87 (52.7%)	86 (57.7%)
	Somewhat unsatisfactory	2295 (34.5%)	1037 (34.4%)	1258 (34.5%)	2177 (34.4%)	109 (34.7%)	60 (36.4%)	49 (32.9%)
	Significantly unsatisfactory	486 (7.3%)	217 (7.2%)	269 (7.4%)	458 (7.2%)	27 (8.6%)	16 (9.7%)	11 (7.4%)
	Totally unsatisfactory	69 (1%)	33 (1.1%)	36 (1.0%)	62 (1.0%)	5 (1.6%)	2 (1.2%)	3 (2.0%)
Anxiety	Mean (SD)	1.3 (0.3)	1.27 (0.28)	1.33 (0.31)*	1.30 (0.30%)	1.36 (0.33)*	1.36 (0.33)	1.36 (0.34)
Depression	Mean (SD)	1.34 (0.34)	1.29 (0.31)	1.39 (0.36)*	1.34 (0.34%)	1.40 (0.35)*	1.38 (0.35)	1.43 (0.35)
Education	Less than 9 years of comprehensive school	306 (5.5%)	152 (5.8%)	154 (5.2%)	280 (5.4%)	19 (7.3%)*	8 (5.6%)	11 (9.4%)
	Comprehensive school	4362 (78.4%)	2053 (78.9%)	2309 (77.9%)	4080 (78.1%)	214 (82.6%)	121 (85.2%)	93 (79.5%)
	High educated	898 (16.1%)	397 (15.3%)	501 (16.9%)	867 (16.6%)	26 (10.0%)	13 (9.2%)	13 (11.1%)
Psoriasis	No	1783 (97.5%)	820 (97.4%)	963 (97.7%)	1686 (97.8%)	91 (92.9%)*	41 (93.2%)	50 (92.6%)
	Yes	45 (2.5%)	22 (2.6%)	23 (2.3%)	38 (2.2%)	7 (7.1%)	3 (6.8%)	4 (7.4%)
Pityriasis versicolor	No	1869 (98.1%)	864 (98.0%)	1005 (98.1%)	1747 (98.2%)	94 (94.9%)*	42 (93.3%)	52 (96.3%)
	Yes	37 (1.9%)	18 (2.0%)	19 (1.9%)	32 (1.8%)	5 (5.1%)	3 (6.7%)	2 (3.7%)
Tinea pedis	No	1392 (73%)	557 (63.2%)	835 (81.5%)*	1316 (74.0%)	55 (55.6%)*	20 (44.4%)	35 (64.8%)
	Yes	514 (27%)	325 (36.8%)	189 (18.5%)	463 (26.0%)	44 (44.4%)	25 (55.6%)	19 (35.2%)
Onychomycosis	No	1726 (90.6%)	757 (85.8%)	969 (94.6%)*	1619 (91.0%)	83 (83.8%)*	34 (75.6%)	49 (90.7%)
	Yes	180 (9.4%)	125 (14.2%)	55 (5.4%)	160 (9.0%)	16 (16.2%)	11 (24.4%)	5 (9.3%)
Café-au-lait spots	No	1668 (87.5%)	776 (88.0%)	892 (87.1%)*	1550 (87.1%)	96 (97.0%)*	45 (100.0%)	51 (94.4%)
	Yes	238 (12.5%)	106 (12.0%)	132 (12.9%)	229 (12.9%)	3 (3.0%)	0 (0.0%)	3 (5.6%)
Lentigo senilis	No	1650 (86.6%)	794 (90.0%)	856 (83.6%)*	1531 (86.1%)	94 (94.9%)*	45 (100.0%)	49 (90.7%)
	Yes	256 (13.4%)	88 (10.0%)	168 (16.4%)	248 (13.9%)	5 (5.1%)	0 (0.0%)	5 (9.3%)
Rosacea	No	1614 (84.8%)	790 (89.6%)	824 (80.6%)*	1513 (85.1%)	76 (76.8%)*	41 (91.1%)	35 (64.8%)
	Yes	290 (15.2%)	92 (10.4%)	198 (19.4%)	264 (14.9%)	23 (23.2%)	4 (8.9%)	19 (35.2%)
Hyperhidrosis	No	1864 (97.8%)	850 (96.4%)	1014 (99.0%)*	1744 (98.0%)	92 (92.9%)*	42 (93.3%)	50 (92.6%)
	Yes	42 (2.2%)	32 (3.6%)	10 (1.0%)	35 (2.0%)	7 (7.1%)	3 (6.7%)	4 (7.4%)
Type 2 diabetes	No	6660 (95.2%)	3027 (94.4%)	3633 (95.9%)*	–	–	–	–
	Yes	336 (4.8%)	180 (5.6%)	156 (4.1%)	–	–	180 (53.6%)	156 (46.4%)

*Statistical significance at the 0.05 level for differences between male and female and differences between people with diabetes and healthy people.

### Variable selection

**Figure 2:**
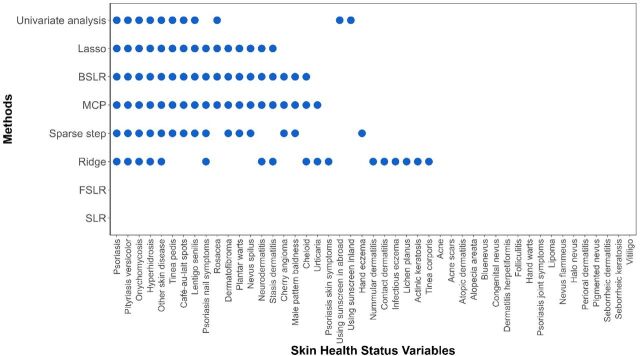
Results of variable selection methods. BSLR, backward stepwise logistic regression; FSLR, forward stepwise logistic regression; MCP, minimax convex penalty; SLR, stepwise logistic regression.

A total of 17 variables were selected using the ridge method, 15 variables with the lasso method, 11 through univariate analysis, 18 with BSLR, 19 with MCP and 15 with sparse stepwise. The FSLR and SLR methods did not select any variables and 18 variables were not selected by any of the methods (figure 2). Significant variables were identified based on predefined, method-specific criteria: ridge and lasso regression retained variables with nonzero coefficients, univariate analysis defined significance at p<0.05 and BSLR and sparse stepwise methods applied model-specific thresholds optimising the predictive performance.

Overall, excluding the two FSLR and SLR methods, the combined agreement among the remaining methods was 0.507, a strong value for six different methods, indicating reliable results. In addition, a pairwise agreement analysis showed that MCP and BSLR, as well as BSLR and Lasso, had the highest agreement values at 0.96 and 0.86, respectively. The lowest agreement was observed between univariate analysis and Ridge (online supplemental table S4). A total of 15 variables were initially selected, each identified by a minimum of 4 different selection methods. From these, to ensure proper control over type I and type II errors, we filtered results with significant p values in the univariate analysis. Machine learning models optimise for prediction accuracy but do not inherently provide measures of statistical significance. By applying a p value threshold, we identified statistically robust associations and reduced the risk of false positives. This approach allowed us to combine the predictive power of machine learning with the inferential strength of traditional statistical methods, ensuring more reliable and interpretable findings. These variables included: tinea pedis, psoriasis, rosacea, onychomycosis, café-au-lait spots, hyperhidrosis, lentigo senilis and pityriasis versicolor. The variable ‘other skin disease’ was eligible for inclusion in the final list of variables based on our selection strategy but was excluded due to its limited informativeness, likely reflecting its broad and non-specific definition during data collection. This highlights the importance of precise variable definitions, as poorly defined variables may reduce their analytical utility.

10.1136/bmjopen-2025-109709.supp5 Supplementary data

The maximum VIF observed was 2.38 (for anxiety and depression), and the mean VIF across all variables was 1.21, both well below the commonly used threshold of 10. This indicates that multicollinearity did not pose a concern in the model (online supplemental table S5).

10.1136/bmjopen-2025-109709.supp6 Supplementary data

### Path analysis

**Figure 3:**
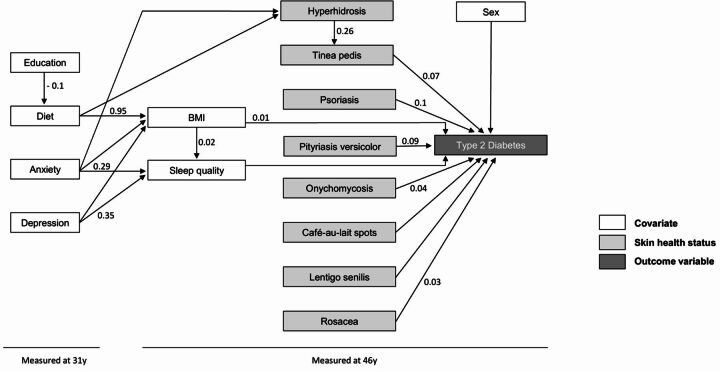
The path analysis was performed in 1906 individuals aged 46 years. All depicted arrows represent statistically significant associations (beta coefficients). Model fit values: SRMR=0.031, RMSEA=0.040. BMI, body mass index; RMSEA, root mean square error of approximation; SRMR, standardised root mean square residual.

The results of the path analysis with the standardised path coefficients are depicted in figure 3 and table 2 and the exponentiation of the coefficients is shown in online supplemental table S6. The model fit was evaluated using several indices. The RMSEA (0.040) and SRMR (0.031) were both below 0.08 and the relative χ² (CMIN/df) was calculated as 4.12, which is <5. These findings indicate an acceptable fit of the model to the data. In the model, sex was not significantly associated with T2D (OR 1.004, 95% CI 0.982 to 1.026), indicating no evidence of a meaningful difference in risk between males and females. BMI had a direct effect on T2D (OR 1.011, 95% CI 1.008 to 1.014) and an indirect effect, mediated through sleep quality (OR 1.00, 95% CI 1.00 to 1.001). The total effect was the sum of direct and indirect effects, at 1.011 with a 95% CI of 1.008 to 1.014. The total effect was significant at the 0.05 level.

10.1136/bmjopen-2025-109709.supp7 Supplementary data

**Table 2:** Standardised path coefficients

	Estimate (95% CI)	P value
T2D		
Sex → T2D	0.004 (−0.018 to 0.026)	0.746
BMI → T2D	0.011 (0.008 to 0.014)	<0.001
Sleep quality → T2D	−0.005 (−0.016 to 0.005)	0.332
Psoriasis → T2D	0.1 (0.056 to 0.145)	<0.001
Pityriasis versicolor → T2D	0.087 (0.037 to 0.137)	0.001
Tinea pedis → T2D	0.065 (0.039 to 0.092)	<0.001
Onychomycosis → T2D	0.041 (0.01 to 0.071)	0.008
Café−au-lait spots → T2D	−0.043 (−0.101 to 0.015)	0.142
Lentigo senilis → T2D	−0.035 (−0.08 to 0.011)	0.136
Rosacea → T2D	0.03 (0.004 to 0.056)	0.023
BMI		
Diet → BMI	0.949 (0.386 to 1.512)	0.001
Anxiety → BMI	0.012 (−0.91 to 0.934)	0.979
Depression → BMI	0.383 (−0.403 to 1.168)	0.340
Diet		
Education → Diet	−0.097 (−0.135 to −0.06)	<0.001
Hyperhidrosis		
Diet → Hyperhidrosis	−0.005 (−0.023 to 0.014)	0.615
Anxiety → Hyperhidrosis	0.018 (−0.023 to 0.059)	0.382
Sleep quality		
BMI → Sleep quality	0.017 (0.004 to 0.03)	0.011
Anxiety → Sleep quality	0.285 (0.022 to 0.549)	0.034
Depression → Sleep quality	0.351 (0.113 to 0.589)	0.004
Tinea pedis		
Hyperhidrosis → Tinea pedis	0.262 (0.131 to 0.392)	<0.001
χ^2^ goodness of fit test	226.773	<0.001
df	55	
CMIN/df	4.12	
RMSEA	0.04	
SRMR	0.031	

Variables listed in the above are standardised variables that are transformed version of an original variable that have been adjusted to have a mean of 0 and an SD of 1.

BMI, body mass index; CMIN/df, χ^2^ minimum discrepancy/df; RMSEA, root mean square error approximation; SRMR, standardised root mean square residual; T2D, type 2 diabetes.

Sleep quality had no significant direct effect on T2D (OR 0.995, 95% CI 0.984 to 1.005). Diet (OR 1, 95% CI 1 to 1.001), anxiety (OR 0.999, 95% CI 0.992 to 1.018), depression (OR 0.998, 95% CI 0.998 to 1.003) and education (OR 1, 95% CI 1 to 1) did not show any indirect effect on T2D. The path analysis showed that tinea pedis (OR 1.067, 95% CI 1.04 to 1.096), psoriasis (OR 1.105, 95% CI 1.058 to 1.156), rosacea (OR 1.03, 95% CI 1.004 to 1.058), onychomycosis (OR 1.042, 95% CI 1.01 to 1.074) and pityriasis versicolor (OR 1.091, 95% CI 1.038 to 1.147) had positive direct effects on T2D. Finally, we observed that hyperhidrosis had a positive indirect effect on T2D mediated through tinea pedis (OR 1.017, 95% CI 1.005 to 1.037) (online supplemental table S6).

## Discussion

To the best of our knowledge, this is the first study to simultaneously investigate multiple skin diseases in relation to T2D and to further incorporate the selected variables into a path analysis. The innovative use of a combination of machine learning techniques and traditional statistical methods for variable selection has led to more robust and high-quality results, with a promising agreement rate of 0.507 across six different methods. This study identified a significant direct association of psoriasis, pityriasis versicolor, tinea pedis, onychomycosis and rosacea with T2D. The corresponding ORs were 1.105 (95% CI 1.058 to 1.156), 1.091 (95% CI 1.038 to 1.147), 1.067 (95% CI 1.04 to 1.096), 1.042 (95% CI 1.01 to 1.074) and 1.03 (95% CI 1.004 to 1.058), respectively.

In our study, we found that the prevalence of psoriasis from self-reported information in the population of 46 years was 2.5% and we did not find any difference between men and women. We identified a significant association between T2D and psoriasis, with individuals who have psoriasis being 1.105 times more likely to develop T2D compared with those without psoriasis. This translates to a 0.105 increase in the odds of having T2D for individuals with psoriasis. These findings align with prior research, such as the meta-analysis conducted by Mirghani *et al,* which reported an OR of 1.38 (95% CI 1.17 to 1.64) for the association between psoriasis and diabetes. It is worth noting that while the meta-analysis included both type 1 and T2D, our study specifically focused on T2D.^21^

It is suggested that diabetes and psoriasis may exacerbate each other, potentially creating a vicious cycle where the presence of one condition aggravates the severity of the other.^22 23^ The link between psoriasis and diabetes has been proposed in other reports demonstrating that psoriasis may increase the risk of developing diabetes. People with psoriasis are more likely to develop diabetes compared with those without the condition, and the risk appears to be even higher in severe cases.^24–26^ Additionally, psoriasis may contribute to insulin resistance, a key factor in the onset of T2D.^27^ On the other hand, diabetes, especially when poorly managed, might also increase the likelihood of developing psoriasis. This complex, possibly bidirectional association suggests that chronic inflammation and immune system dysfunction are central to the development of both diseases. This may be explained by the shared genes and molecular pathways recently identified to be linked with T2D and psoriasis pathogenesis.^28^ This bidirectional association highlights the importance of careful management and monitoring of both conditions to prevent worsening outcomes.

In addition, we examined three fungal diseases: pityriasis versicolor, tinea pedis and onychomycosis. We observed a direct association between diabetes and all three conditions. Our findings regarding fungal infections are consistent with previous research.^21 22^ Saud *et al* reported that the prevalence of fungal diseases in diabetic patients is 7.2 times higher compared with individuals without diabetes.^29^ Furthermore, participants exhibiting abnormal skin findings in the toe web areas had a 2.5 times increased risk of having undiagnosed diabetes based on OGTT results. The risk was even higher (six times) when assessed using haemoglobin A1c (HbA1c) levels.^30^ This highlights the potential role of fungal disease, particularly in the toe web regions, as an indicator for identifying individuals at higher risk of diabetes, which may facilitate earlier detection and intervention.

In our study, individuals with pityriasis versicolor, tinea pedis and onychomycosis were found to be 1.09, 1.07 and 1.04 times more likely, respectively, to present with T2D compared with those without these fungal infections. These results highlight the importance of monitoring and managing fungal infections in patients with diabetes to potentially mitigate the risk and complications associated with T2D.

Furthermore, this study is the first to investigate the association between café-au-lait spots and lentigo senilis with T2D. However, we did not identify a statistically significant association between either of these skin conditions and T2D. Further studies are warranted to confirm these findings and to better understand any potential association. Regarding rosacea, we identified a direct association with T2D. Individuals with rosacea were found to be 1.03 times more likely to present with T2D compared with those without this skin condition. Interestingly, a study by Stefanadi *et al* identified T2D as a risk factor for rosacea.^10^ This hypothesis has been addressed by Spoendlin *et al*, who conducted a population-based case–control analysis using the UK-based General Practice Research Database to analyse the association between rosacea and T2D, reporting a significantly reduced risk of rosacea among individuals with diabetes mellitus (OR=0.80, 95% CI 0.74 to 0.85). This inverse association was more pronounced in individuals with higher HbA1c levels and a longer diabetes duration, suggesting that advanced stages of diabetes may be linked to a lower likelihood of developing rosacea.^31^

Moreover, our study identified hyperhidrosis as an indirect risk factor for T2D. This finding aligns with the literature, which indicates that individuals with T2D exhibit impaired thermoregulation compared with healthy individuals, resulting in sweating dysfunction. This impairment may be attributed to several factors, including cardiovascular complications, metabolic imbalances such as irregular insulin distribution and chronic hyperglycaemia, alterations in autonomic nervous function and reduced blood flow to the skin. Collectively, these factors may influence the sweat gland’s function leading to a diminished ability to sweat.^32–36^

### Limitations

This study has several limitations. In the NFBC1966 study, data on skin conditions were collected by dermatologists at the age of 46 for individuals who were readily accessible and primarily residing in northern Finland. While the involvement of dermatologists enhances the validity of the data, this regional focus, along with the limited sample size, restricts the generalisability of the findings to the broader Finnish population. Additionally, as the data used in this study were collected in 1997 and 2012, the findings may not fully reflect current population characteristics or trends, which could limit their applicability to present-day contexts. Furthermore, the definition of T2D was based on the OGTT results, self-reports and antidiabetic medication use, which may introduce potential misclassification.

In addition, attrition and loss to follow-up are common in longitudinal studies. In the NFBC1966 cohort, differences in characteristics were observed between participants who participated in the 46-year follow-up and those who did not. These differences suggest the possibility of differential attrition, which may introduce selection bias and should be considered when interpreting the findings.

Furthermore, the data were collected at a single time point, so it was impossible to conduct a longitudinal assessment of skin diseases. The relatively small sample size also limited the statistical power and restricted the feasibility of applying more complex models. Consequently, we had to rely on simpler statistical models and associations to maintain the validity of our findings. Moreover, this was an observational study with a cross-sectional assessment where external factors were not controlled, making it impossible to establish causal relationships. Therefore, it is recommended to use a randomised controlled trial study to investigate causal relationships or employ genetic methods such as Mendelian Randomisation to explore the causal link between skin diseases and diabetes.

### Implications

These results may have important implications for people’s health and quality of life. First, skin diseases are often considered insignificant, and particularly when accompanied by other illnesses, their treatment is often given lower priority. However, people’s skin health status is closely linked to their overall health and neglecting it may lead to more serious complications. Second, in people with T2D, skin problems are frequently overlooked until they become severe. Our study has shown that skin diseases are associated with T2D. Based on these findings, we strongly recommend that healthcare providers pay more attention to skin health in people with T2D, ensuring timely diagnosis and treatment to prevent complications and improve people’s overall well-being.

## Conclusions

In this study, our aim was to investigate the pathway effects of skin diseases on T2D. We employed advanced statistical techniques for variable selection and innovatively combined machine learning approaches with traditional methods, ensuring more robust and reliable findings. The results of a path analysis revealed that psoriasis, pityriasis versicolor, tinea pedis, onychomycosis and rosacea had a positive direct effect on T2D.

Finally, it is essential to note that skin problems are common in patients with T2D and can be indicative of poor blood sugar control. Early diagnosis and appropriate treatment of these issues can significantly improve patients’ quality of life.

## Supplementary Material

Reviewer comments

Author's manuscript

## Data Availability

Data sharing not applicable as no datasets generated and/or analysed for this study. NFBC data are available from the University of Oulu, Infrastructure for Population Studies. Permission to use the data can be applied for research purposes via an electronic material request portal. In the use of data, we follow the EU general data protection regulation (679/2016) and the Finnish Data Protection Act. The use of personal data is based on a cohort participant’s written informed consent in their latest follow-up study, which may pose limitations on its use. Please, contact the NFBC project centre (NFBCprojectcenter(at)oulu.fi) and visit the cohort website (www.oulu.fi/nfbc) for more information.
