# Supplementary material for: Assessing the association of type 2 diabetes with skin health status: a study of the Northern Finland Birth Cohort 1966
Source: BMJ Open. 2026 Jul 10;16(7):e109709. doi: 10.1136/bmjopen-2025-109709 (PMC13358341; doi:10.1136/bmjopen-2025-109709)
Supplement: Supplementary data [file bmjopen-16-7-s002.pdf]

Table S1 Descriptive characteristics of the study population

Variable	Category	Overall	Missing	Men	Women	P-value[a]	Healthy	Type 2 diabetes	P-value[b]
Tinea pedis	No	1392 (73.0 %)	0 (0 %)	557 (63.2 %)	835 (81.5 %)	<0.001	1316 (74.0 %)	55 (55.6 %)	<0.001
	Yes	514 (27.0 %)		325 (36.8 %)	189 (18.5 %)		463 (26.0 %)	44 (44.4 %)	
Hyperhidrosis	No	1864 (97.8 %)	0 (0 %)	850 (96.4 %)	1014 (99.0 %)	<0.001	1744 (98.0 %)	92 (92.9 %)	0.003
	Yes	42 (2.2 %)		32 (3.6 %)	10 (1.0 %)		35 (2.0 %)	7 (7.1 %)	
Café-au-lait spots	No	1668 (87.5 %)	0 (0 %)	776 (88.0 %)	892 (87.1 %)	0.614	1550 (87.1 %)	96 (97.0 %)	0.006
	Yes	238 (12.5 %)		106 (12.0 %)	132 (12.9 %)		229 (12.9 %)	3 (3.0 %)	
Pigmented nevus	No	1685 (88.4 %)	0 (0 %)	766 (86.8 %)	919 (89.7 %)	0.058	1572 (88.4 %)	88 (88.9 %)	1
	Yes	221 (11.6 %)		116 (13.2 %)	105 (10.3 %)		207 (11.6 %)	11 (11.1 %)	
Lentigo senilis	No	1650 (86.6 %)	0 (0 %)	794 (90.0 %)	856 (83.6 %)	<0.001	1531 (86.1 %)	94 (94.9 %)	0.018
	Yes	256 (13.4 %)		88 (10.0 %)	168 (16.4 %)		248 (13.9 %)	5 (5.1 %)	
Psoriasis	No	1783 (97.5 %)	78 (4.04 %)	820 (97.4 %)	963 (97.7 %)	0.815	1686 (97.8 %)	91 (92.9 %)	0.006
	Yes	45 (2.5 %)		22 (2.6 %)	23 (2.3 %)		38 (2.2 %)	7 (7.1 %)	
Psoriasis skin symptoms	No	1862 (97.9 %)	4 (0.21 %)	856 (97.4 %)	1006 (98.3 %)	0.198	1741 (98.0 %)	93 (94.9 %)	0.084
	Yes	40 (2.1 %)		23 (2.6 %)	17 (1.7 %)		35 (2.0 %)	5 (5.1 %)	
Psoriasis nail symptoms	No	1889 (99.4 %)	5 (0.26 %)	869 (98.9 %)	1020 (99.8 %)	0.022	1763 (99.3 %)	98 (100.0 %)	0.868
	Yes	12 (0.6 %)		10 (1.1 %)	2 (0.2 %)		12 (0.7 %)	0 (0.0 %)	
Psoriasis joint symptoms	No	1888 (99.3 %)	5 (0.26 %)	874 (99.4 %)	1014 (99.2 %)	0.775	1763 (99.3 %)	97 (99.0 %)	1
	Yes	13 (0.7 %)		5 (0.6 %)	8 (0.8 %)		12 (0.7 %)	1 (1.0 %)	
Rosacea	No	1614 (84.8 %)	2 (0.1 %)	790 (89.6 %)	824 (80.6 %)	<0.001	1513 (85.1 %)	76 (76.8 %)	0.035
	Yes	290 (15.2 %)		92 (10.4 %)	198 (19.4 %)		264 (14.9 %)	23 (23.2 %)	
Stasis dermatitis	No	1890 (99.2 %)	1 (0.05 %)	874 (99.1 %)	1016 (99.3 %)	0.773	1765 (99.3 %)	97 (98.0 %)	0.411
	Yes	15 (0.8 %)		8 (0.9 %)	7 (0.7 %)		13 (0.7 %)	2 (2.0 %)	
Bluenevus	No	1880 (98.6 %)	0 (0 %)	874 (99.1 %)	1006 (98.2 %)	0.162	1755 (98.7 %)	97 (98.0 %)	0.909
	Yes	26 (1.4 %)		8 (0.9 %)	18 (1.8 %)		24 (1.3 %)	2 (2.0 %)	
Nevus spilus	No	1817 (95.3 %)	0 (0 %)	843 (95.6 %)	974 (95.1 %)	0.714	1691 (95.1 %)	98 (99.0 %)	0.121

	Yes	89 (4.7 %)		39 (4.4 %)	50 (4.9 %)		88 (4.9 %)	1 (1.0 %)	
Halo nevus	No	1891 (99.2 %)	0 (0 %)	876 (99.3 %)	1015 (99.1 %)	0.819	1764 (99.2 %)	99 (100.0 %)	0.736
	Yes	15 (0.8 %)		6 (0.7 %)	9 (0.9 %)		15 (0.8 %)	0 (0.0 %)	
Dermatofibroma	No	1481 (77.7 %)	0 (0 %)	730 (82.8 %)	751 (73.3 %)	<0.001	1390 (78.1 %)	71 (71.7 %)	0.17
	Yes	425 (22.3 %)		152 (17.2 %)	273 (26.7 %)		389 (21.9 %)	28 (28.3 %)	
Cherry angioma	No	759 (39.8 %)	1 (0.05 %)	410 (46.5 %)	349 (34.1 %)	<0.001	705 (39.7 %)	44 (44.4 %)	0.4
	Yes	1146 (60.2 %)		472 (53.5 %)	674 (65.9 %)		1073 (60.3 %)	55 (55.6 %)	
Cheloid	No	1857 (97.4 %)	0 (0 %)	859 (97.4 %)	998 (97.5 %)	1	1735 (97.5 %)	95 (96.0 %)	0.526
	Yes	49 (2.6 %)		23 (2.6 %)	26 (2.5 %)		44 (2.5 %)	4 (4.0 %)	
Seborrheic keratosis	No	1061 (55.7 %)	0 (0 %)	522 (59.2 %)	539 (52.6 %)	0.005	987 (55.5 %)	58 (58.6 %)	0.616
	Yes	845 (44.3 %)		360 (40.8 %)	485 (47.4 %)		792 (44.5 %)	41 (41.4 %)	
Lipoma	No	1845 (96.8 %)	0 (0 %)	841 (95.4 %)	1004 (98.0 %)	0.001	1726 (97.0 %)	94 (94.9 %)	0.389
	Yes	61 (3.2 %)		41 (4.6 %)	20 (2.0 %)		53 (3.0 %)	5 (5.1 %)	
Congenital nevus	No	1828 (95.9 %)	0 (0 %)	838 (95.0)	990 (96.7 %)	0.086	1703 (95.7 %)	98 (99.0 %)	0.183
	Yes	78 (4.1 %)		44 (5.0 %)	34 (3.3 %)		76 (4.3 %)	1 (1.0 %)	
Nevus flammeus	No	1419 (74.4 %)	0 (0 %)	690 (78.2 %)	729 (71.2 %)	0.001	1316 (74.0 %)	79 79.8 %)	0.241
	Yes	487 (25.6 %)		192 (21.8 %)	295 (28.8 %)		463 (26.0 %)	20 (20.2 %)	
Actinic keratosis	No	1894 (99.4 %)	0 (0 %)	875 (99.2 %)	1019 (99.5 %)	0.582	1767 (99.3 %)	99 (100.0 %)	0.864
	Yes	12 (0.6 %)		7 (0.8 %)	5 (0.5 %)		12 (0.7 %)	0 (0.0 %)	
Atopic dermatitis	No	1813 (95.2 %)	1 (0.05 %)	847 (96.0 %)	966 (94.4 %)	0.128	1689 (95.0 %)	97 (98.0 %)	0.269
	Yes	92 (4.8 %)		35 (4.0 %)	57 (5.6 %)		89 (5.0 %)	2 (2.0 %)	
Hand eczema	No	1736 (91.1 %)	1 (0.05 %)	817 (92.6 %)	919 (89.8 %)	0.039	1614 (90.8 %)	95 (96.0 %)	0.115
	Yes	169 (8.9 %)		65 (7.4 %)	104 (10.2 %)		164 (9.2 %)	4 (4.0 %)	
Seborrheic dermatitis	No	1765 (92.7 %)	1 (0.05 %)	774 (87.8 %)	991 (96.9 %)	<0.001	1650 (92.8 %)	90 (90.9 %)	0.613
	Yes	140 (7.3 %)		108 (12.2 %)	32 (3.1 %)		128 (7.2 %)	9 (9.1 %)	
Nummular dermatitis	No	1869 (98.1 %)	1 (0.05 %)	858 (97.3 %)	1011 (98.8 %)	0.021	1746 (98.2 %)	96 (97.0 %)	0.618
	Yes	36 (1.9 %)		24 (2.7 %)	12 (1.2 %)		32 (1.8 %)	3 (3.0 %)	
Infectious eczema	No	1878 (98.6 %)	1 (0.05 %)	862 (97.7 %)	1016 (99.3 %)	0.007	1754 (98.7 %)	96 (97.0 %)	0.351

	Yes	27 (1.4 %)		20 (2.3 %)	7 (0.7 %)		24 (1.3 %)	3 (3.0 %)	
Contact dermatitis	No	1887 (99.1 %)	1 (0.05 %)	880 (99.8 %)	1007 (98.4 %)	0.006	1761 (99.0 %)	99 (100.0 %)	0.665
	Yes	18 (0.9 %)		2 (0.2 %)	16 (1.6 %)		17 (1.0 %)	0 (0.0 %)	
Neurodermatitis	No	1871 (98.2 %)	1 (0.05 %)	857 (97.2 %)	1014 (99.1 %)	0.002	1748 (98.3 %)	95 (96.0 %)	0.186
	Yes	34 (1.8 %)		25 (2.8 %)	9 (0.9 %)		30 (1.7 %)	4 (4.0 %)	
Lichen planus	No	1893 (99.3 %)	0 (0 %)	874 (99.1 %)	1019 (99.5 %)	0.407	1766 (99.3 %)	99 (100.0 %)	0.817
	Yes	13 (0.7 %)		8 (0.9 %)	5 (0.5 %)		13 (0.7 %)	0 (0.0 %)	
Dermatitis herpetiformis	No	1900 (99.7 %)	0 (0 %)	880 (99.8 %)	1020 (99.6 %)	0.821	1773 (99.7 %)	99 (100.0 %)	1
	Yes	6 (0.3 %)		2 (0.2 %)	4 (0.4 %)		6 (0.3 %)	0 (0.0 %)	
Vitiligo	No	1874 (98.3 %)	0 (0 %)	872 (98.9 %)	1002 (97.9 %)	0.123	1748 (98.3 %)	98 (99.0 %)	0.881
	Yes	32 (1.7 %)		10 (1.1 %)	22 (2.1 %)		31 (1.7 %)	1 (1.0 %)	
Urticaria	No	1896 (99.5 %)	0 (0 %)	877 (99.4 %)	1019 (99.5 %)	1	1770 (99.5 %)	98 (99.0 %)	1
	Yes	10 (0.5 %)		5 (0.6 %)	5 (0.5 %)		9 (0.5 %)	1 (1.0 %)	
Acne	No	1755 (92.1 %)	1 (0.05 %)	818 (92.7 %)	937 (91.6 %)	0.399	1635 (92.0 %)	94 (94.9 %)	0.377
	Yes	150 (7.9 %)		64 (7.3 %)	86 (8.4 %)		143 (8.0 %)	5 (5.1 %)	
Perioral dermatitis	No	1888 (99.3 %)	4 (0.21 %)	881 (99.9 %)	1007 (98.7 %)	0.007	1762 (99.3 %)	98 (99.0 %)	1
	Yes	14 (0.7 %)		1 (0.1 %)	13 (1.3 %)		13 (0.7 %)	1 (1.0 %)	
Acne scars	No	1716 (90.2 %)	4 (0.21 %)	780 (88.5 %)	936 (91.7 %)	0.026	1602 (90.2 %)	89 (90.8 %)	0.981
	Yes	186 (9.8 %)		101 (11.5 %)	85 (8.3 %)		174 (9.8 %)	9 (9.2 %)	
Pityriasis versicolor	No	1869 (98.1 %)	0 (0 %)	864 (98.0 %)	1005 (98.1 %)	0.9	1747 (98.2 %)	94 (94.9 %)	0.058
	Yes	37 (1.9 %)		18 (2.0 %)	19 (1.9 %)		32 (1.8 %)	5 (5.1 %)	
Onychomycosis	No	1726 (90.6 %)	0 (0 %)	757 (85.8 %)	969 (94.6 %)	<0.001	1619 (91.0 %)	83 (83.8 %)	0.027
	Yes	180 (9.4 %)		125 (14.2 %)	55 (5.4 %)		160 (9.0 %)	16 (16.2 %)	
Tinea corporis	No	1898 (99.6 %)	0 (0 %)	878 (99.5 %)	1020 (99.6 %)	1	1772 (99.6 %)	98 (99.0 %)	0.901
	Yes	8 (0.4 %)		4 (0.5 %)	4 (0.4 %)		7 (0.4 %)	1 (1.0 %)	
Folliculitis	No	1790 (93.9 %)	0 (0 %)	796 (90.2 %)	994 (97.1 %)	<0.001	1667 (93.7 %)	95 (96.0 %)	0.488
	Yes	116 (6.1 %)		86 (9.8 %)	30 (2.9 %)		112 (6.3 %)	4 (4.0 %)	

Plantar warts	No	1732 (90.9 %)	0 (0 %)	809 (91.7 %)	923 (90.1 %)	0.263	1613 (90.7 %)	94 (94.9 %)	0.207
	Yes	174 (9.1 %)		73 (8.3 %)	101 (9.9 %)		166 (9.3 %)	5 (5.1 %)	
Hand warts	No	1855 (97.3 %)	0 (0 %)	860 (97.5 %)	995 (97.2 %)	0.754	1731 (97.3 %)	96 (97.0 %)	1
	Yes	51 (2.7 %)		22 (2.5 %)	29 (2.8 %)		48 (2.7 %)	3 (3.0 %)	
Alopecia areata	No	1899 (99.6 %)	0 (0 %)	877 (99.4 %)	1022 (99.8 %)	0.338	1773 (99.7 %)	99 (100.0 %)	1
	Yes	7 (0.4 %)		5 (0.6 %)	2 (0.2 %)		6 (0.3 %)	0 (0.0 %)	
Male pattern baldness	No	1291 (67.7 %)	0 (0 %)	280 (31.7 %)	1011 (98.7 %)	<0.001	1202 (67.6 %)	69 (69.7 %)	0.741
	Yes	615 (32.3 %)		602 (68.3 %)	13 (1.3 %)		577 (32.4 %)	30 (30.3 %)	
Other skin disease	No	1595 (90.2 %)	0 (0 %)	753 (91.2 %)	842 (89.4 %)	0.453	1497 (90.6 %)	74 (82.2 %)	0.021
	Yes	168 (88.4 %)		71 (8.6 %)	97 (10.3 %)		150 (9.1 %)	16 (17.8 %)	
	Perhaps	5 (11.6 %)		2 (0.2 %)	3 (0.3 %)		5 (0.3 %)	0 (0.0 %)	
Using sunscreen inland	Not at all	260 (14.1 %)	60 (3.11 %)	190 (22.3 %)	70 (7.0 %)	<0.001	241 (13.9 %)	18 (18.4 %)	0.093
	Sometimes	890 (48.2 %)		458 (53.7 %)	432 (43.5 %)		838 (48.2 %)	47 (48.0 %)	
	Requlary	614 (33.3 %)		171 (20.0 %)	443 (44.6 %)		587 (33.7 %)	25 (25.5 %)	
	I do not spend time in the sun	82 (4.4 %)		34 (4.0 %)	48 (4.8 %)		74 (4.3 %)	8 (8.2 %)	
Using sunscreen in abroad	Not at all	230 (13.1 %)	151 (7.82 %)	147 (18.3 %)	83 (8.7 %)	<0.001	216 (13.0 %)	13 (14.4 %)	0.658
	Sometimes	147 (8.4 %)		93 (11.6 %)	54 (5.7 %)		139 (8.4 %)	7 (7.8 %)	
	Requlary	1172 (66.8 %)		472 (58.8 %)	700 (73.5 %)		1111 (67.0 %)	56 (62.2 %)	
	I do not spend time in the sun	206 (11.7 %)		91 (11.3 %)	115 (12.1 %)		192 (11.6 %)	14 (15.6 %)	

P-value[a] for differences between male and female. P-value[b] for differences between people with diabetes and healthy people.
