# Supplementary material for: Assessing the association of type 2 diabetes with skin health status: a study of the Northern Finland Birth Cohort 1966
Source: BMJ Open. 2026 Jul 10;16(7):e109709. doi: 10.1136/bmjopen-2025-109709 (PMC13358341; doi:10.1136/bmjopen-2025-109709)
Supplement: Supplementary data [file bmjopen-16-7-s003.pdf]

*Table S2 Distribution of study covariates at 31-year who participated in the 46-year follow-up and those who did not*

| Variable | Participated in the 46y | Not participated | P-value |
|---|---|---|---|
| **Sex** | | | <0.001 |
| Male | 3280 (46 %) | 2935 (58 %) | |
| Female | 3835 (54 %) | 2086 (42 %) | |
| **Education, N (%)** | | | <0.001 |
| <9 years | 306 (5.5%) | 223 (11.1%) | |
| Comprehensive school | 4359 (78.4%) | 1553 (77.2%) | |
| Highly educated | 898 (16.1%) | 235 (11.7%) | |
| **Diet, N (%)** | | | <0.001 |
| Unhealthy | 5522 (88.8%) | 1929 (85.0%) | |
| Healthy | 694 (11.2%) | 341 (15.0%) | |
| **Anxiety, Mean (sd)** | 1.304 (0.299) | 1.339 (0.342) | <0.001 |
| **Depression, Mean (sd)** | 1.345 (0.345) | 1.382 (0.377) | <0.001 |
