# Supplementary material for: Assessing the association of type 2 diabetes with skin health status: a study of the Northern Finland Birth Cohort 1966
Source: BMJ Open. 2026 Jul 10;16(7):e109709. doi: 10.1136/bmjopen-2025-109709 (PMC13358341; doi:10.1136/bmjopen-2025-109709)
Supplement: Supplementary data [file bmjopen-16-7-s004.pdf]

Table S3 Distribution of study covariates at 31-year and 46-year follow-ups

Variable	31-year follow-up	46-year follow-up	P-value
**Education, N (%)**			<0.001
<9 years	72 (4.71 %)	38 (2.08 %)	
Comprehensive school	1221 (79.91 %)	1310 (71.70 %)	
Highly educated	235 (15.38 %)	479 (26.22 %)	
Missing, N	378	79	
**Diet, N (%)**			<0.001
Unhealthy	1518 (88.51 %)	1603 (93.90 %)	
Healthy	197 (11.49 %)	401 (6.10 %)	
Missing, N	191	199	
**Anxiety, Mean (sd)**	1.29 (0.28)	1.29 (0.30)	0.8113
Missing, N	183	112	
**Depression, Mean (sd)**	1.32 (0.32)	1.35 (0.37)	0.0019
Missing, N	203	112	
