# Supplementary figures and images for: Assessing the association of type 2 diabetes with skin health status: a study of the Northern Finland Birth Cohort 1966
Source: BMJ Open. 2026 Jul 10;16(7):e109709. doi: 10.1136/bmjopen-2025-109709 (PMC13358341; doi:10.1136/bmjopen-2025-109709)

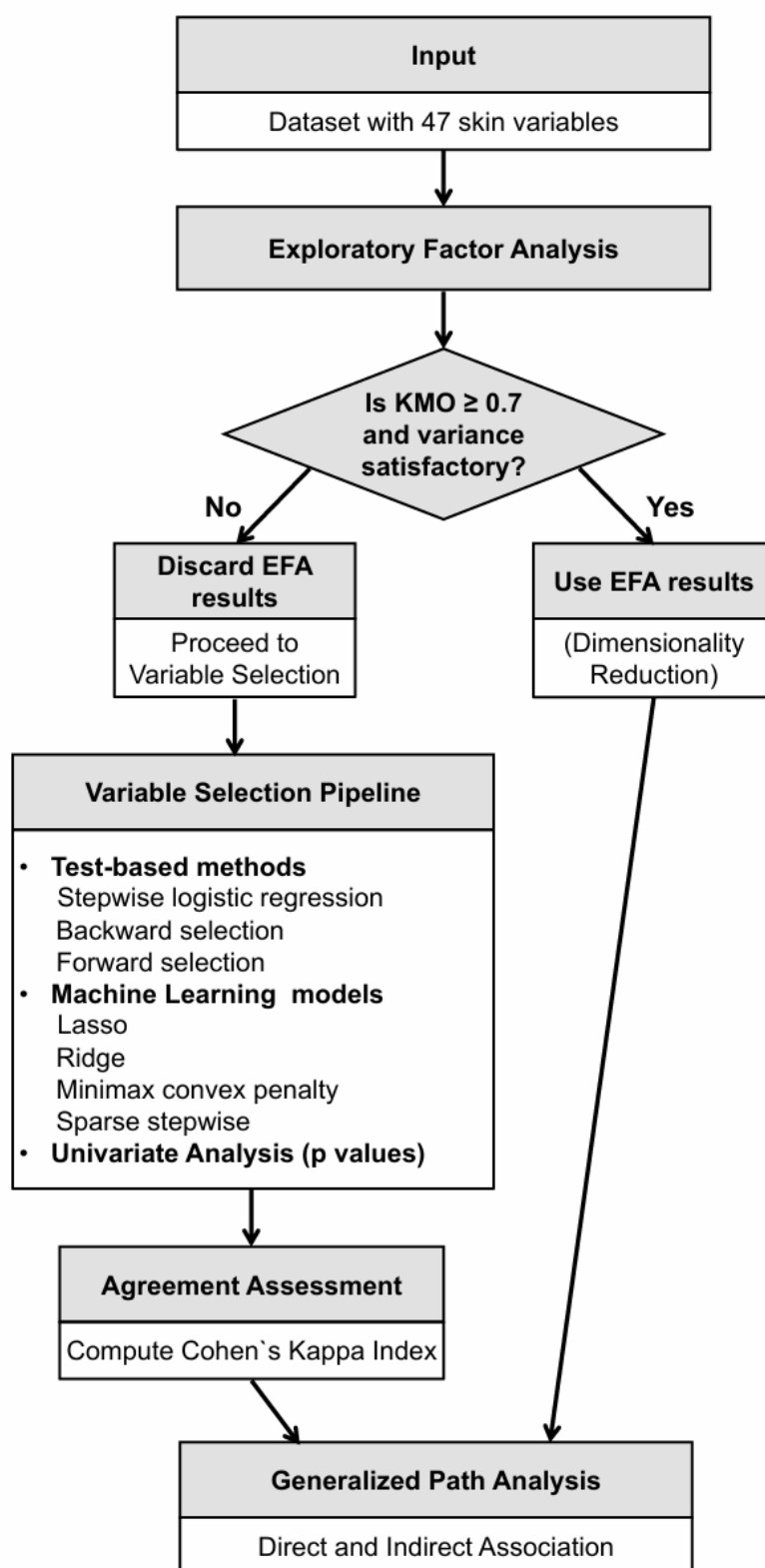

Figure S1 Flow chart demonstrating the analytical plan

Supplement: Supplementary data [file bmjopen-16-7-s001.pdf]
