# Supplementary material for: Assessing the association of type 2 diabetes with skin health status: a study of the Northern Finland Birth Cohort 1966
Source: BMJ Open. 2026 Jul 10;16(7):e109709. doi: 10.1136/bmjopen-2025-109709 (PMC13358341; doi:10.1136/bmjopen-2025-109709)
Supplement: Supplementary data [file bmjopen-16-7-s005.pdf]

Table S4 Agreement coefficient between different methods

Method 1	Method 2	Cohen kappa (95% CI)	P_value
Ridge	Lasso	0.24 (-0.043, 0.53)	0.094
Ridge	Univariate analysis	0.16 (-0.122, 0.448)	0.248
Ridge	BSLR	0.23 (-0.058, 0.511)	0.12
Ridge	MCP	0.28 (0.002, 0.56)	0.053
Ridge	Sparse step	0.05 (-0.233, 0.342)	0.708
Lasso	Univariate analysis	0.53 (0.27, 0.799)	<0.001
Lasso	BSLR	0.86 (0.709, 1)	<0.001
Lasso	MCP	0.82 (0.649, 0.986)	<0.001
Lasso	Sparse step	0.71 (0.488, 0.924)	<0.001
Univariate analysis	BSLR	0.42 (0.159, 0.687)	0.002
Univariate analysis	MCP	0.39 (0.128, 0.651)	0.005
Univariate analysis	Sparse step	0.43 (0.151, 0.711)	0.003
BSLR	MCP	0.96 (0.869, 1)	<0.001
BSLR	Sparse step	0.77 (0.577, 0.958)	<0.001
MCP	Sparse step	0.73 (0.524, 0.927)	<0.001
