# Supplementary material for: Assessing the association of type 2 diabetes with skin health status: a study of the Northern Finland Birth Cohort 1966
Source: BMJ Open. 2026 Jul 10;16(7):e109709. doi: 10.1136/bmjopen-2025-109709 (PMC13358341; doi:10.1136/bmjopen-2025-109709)
Supplement: Supplementary data [file bmjopen-16-7-s006.pdf]

Table S5 Variance inflation factor for selected variables

Variable	VIF
BMI	1.08
Diet	1.04
Poor Quality of sleep	1.06
Anxiety	2.38
Depression	2.38
Education	1.06
Psoriasis	1.03
Pityriasis versicolor	1.02
Tinea pedis	1.16
Onychomycosis	1.12
Café_au_lait_spots	1.01
Lentigo senilis	1.01
Rosacea	1.04
Hyperhidrosis	1.05
