# Supplementary material for: Assessing the association of type 2 diabetes with skin health status: a study of the Northern Finland Birth Cohort 1966
Source: BMJ Open. 2026 Jul 10;16(7):e109709. doi: 10.1136/bmjopen-2025-109709 (PMC13358341; doi:10.1136/bmjopen-2025-109709)
Supplement: Supplementary data [file bmjopen-16-7-s007.pdf]

Table S6 Exponentiated Path coefficient analysis

Independent Variable	Total Effect	Direct Effect	Indirect Effect
Sex	1.004 (0.982, 1.026)	1.004 (0.982, 1.026)	-
BMI	1.011 (1.008, 1.014)	1.011 (1.008, 1.014)	1 (1, 1.001)
Diet	1 (1, 1.001)	-	1 (1, 1.001)
Sleep quality	0.995 (0.984, 1.005)	0.995 (0.984, 1.005)	-
Anxiety	0.999 (0.992, 1.018)	-	0.999 (0.992, 1.018)
Depression	0.998 (0.998, 1.003)	-	0.998 (0.998, 1.003)
Education	1 (1, 1)	-	1 (1, 1)
Psoriasis	1.105 (1.058, 1.156)	1.105 (1.058, 1.156)	-
Pityriasis versicolor	1.091 (1.038, 1.147)	1.091 (1.038, 1.147)	-
Tinea pedis	1.067 (1.04, 1.096)	1.067 (1.04, 1.096)	-
Onychomycosis	1.042 (1.01, 1.074)	1.042 (1.01, 1.074)	-
Café-au-lait spots	0.958 (0.904, 1.015)	0.958 (0.904, 1.015)	-
Lentigo senilis	0.966 (0.923, 1.011)	0.966 (0.923, 1.011)	-
Rosacea	1.03 (1.004, 1.058)	1.03 (1.004, 1.058)	-
Hyperhidrosis	1.017 (1.005, 1.037)	-	1.017 (1.005, 1.037)
