# Supplementary material for: Assessing the association of type 2 diabetes with skin health status: a study of the Northern Finland Birth Cohort 1966
Source: BMJ Open. 2026 Jul 10;16(7):e109709. doi: 10.1136/bmjopen-2025-109709 (PMC13358341; doi:10.1136/bmjopen-2025-109709)
Supplement: Reviewer comments [file bmjopen-2025-109709.reviewer_comments.pdf]

PEER REVIEW HISTORY

BMJ Open publishes all reviews undertaken for accepted manuscripts. Reviewers are asked to complete a checklist review form and are provided with free text boxes to elaborate on their assessment. These free text comments are reproduced below.

ARTICLE DETAILS

Title (Provisional)

Assessing the association of Type 2 diabetes with skin health status: A study of the Northern Finland Birth Cohort 1966

Authors

Nasserinejad, Maryam; Huilaja, Laura; Sinikumpu, Suvi-Päivikki; Vainio, Seppo; Röning, Juha; Ali, Nsrein; Sebert, Sylvain

---

VERSION 1 - REVIEW

**Reviewer**	**1**
**Name**	**Kelson, Zoe**
**Affiliation**	**University of Exeter, Mathematics**
**Date**	**06-Oct-2025**
**COI**	**None**

This study applies variable selection methods to identify key dermatological factors linked to T2D and examines their direct and indirect associations using path analysis.

Reviewer comments:

"Of 47 skin-related variables, key predictors were selected using test-based and penalty-based methods." [Abstract]

Can these methods please be specified in the Abstract?

"We analyzed data from the Northern Finland Birth Cohort 1966 (NFBC1966), including 1,930 participants (1,036 women, 894 men) at ages 31 and 46"

and

"The cohort comprises 12138 live births from 1966, representing 96.3% of all births that year in Finland's two northernmost provinces, Oulu and Lapland. Data collection began during pregnancy, starting between the 10th and 16th gestational weeks, and has continued through multiple follow-up stages, including assessments at ages 1, 14, 31, and 46...

The current study aimed at including all participants with available data on skin health status and type 2 diabetes based on their participation to follow-ups organized when the

participants were approximately 46 years (in 2012), while also utilizing covariate data from age 31 (collected in 1997). Following logistic and financial constraints, the clinical examination for dermatology diagnosis was only intended to participants living in the region of Oulu and was conducted at the University Hospital of Oulu. It included a total of 894 males and 1036 females (N = 1930)."

Can the authors please further comment on whether the analysed sample can be considered to be representative?

"T2D was defined based on oral glucose tolerance tests, self-reports, and Metformin prescriptions. Skin conditions were assessed through dermatologist diagnoses and questionnaires. "

Can the authors please acknowledge the potential for self-reporting bias?

"To account for the long-term impact of diet, education, anxiety, and depression, we employed data from 31 ages in this study...

Information on poor quality sleep was collected by questionnaire at age 46 years. "

Can the authors please consider examining longitudinal data (i.e. at multiple ages) for changes over time?

"Chi-square tests were used to compare categorical variables between sex groups and between health status groups as presented... including Single covariate techniques based on p-values, as well as backward stepwise logistic regression (BSLR), forward stepwise logistic regression (FSLR), and stepwise logistic regression (SLR). Additionally, we utilized machine learning techniques, specifically least absolute shrinkage and selection operator regression (Lasso), Ridge regression, minimax convex penalty (MCP), and Sparse Stepwise Regression. The degree of agreement between the results of various methods was assessed using Cohen's Kappa index"

A range of appropriate analytical methodologies have been applied.

Can these methods please be further described in the Statistical Analysis section, including specifying the 'single covariate techniques' referred to here?

"Following the selection of skin health status, we employed path analysis to examine both direct and indirect associations, while controlling for the effects of sex, body mass index (BMI), diet, education, depression, and anxiety (Supplementary Fig. 1)"

and

"Table 1 Descriptive characteristics of the study population"

Can other factors please be reported and controlled for, including race or ethnicity and medications for instance?

"A total of 15 variables were initially selected using various methods. From these, to ensure proper control over Type I and Type II errors, we filtered results with significant p-values in the single covariate analysis."

Can the authors please also demonstrate that the explored variables are also clinically meaningful?

"It is worth noting that the multicollinearity of the selected variables was assessed by the variance inflation factor (VIF) (Supplementary Table3)"

Can this methodology assessing collinearity please also be specified in the Statistical Analysis section, with an interpretation of outcomes summarised in the Result text?

"Model fit was evaluated using several indices. The RMSEA (0.060) and SRMR (0.045) were both below 0.08 and the relative Chi-square (CMIN/DF) was calculated as 5.89, which is <6."

Can the assessment of model performance metrics please be specified in the Statistical Analysis section?

"Figure 1 Flow chart demonstrating the process used to select the study participants"

Can the authors please further comment on missing data?

How much was missing for each variable or factor, and can it be considered to be missing at random?

Can the authors please clarify how missing data was handled in the analysis?

"However, these studies have faced limitations. In the NFBC1966 study, data on skin conditions were collected by dermatologists at the age of 46 for individuals who were readily accessible, primarily residing in northern Finland. While the involvement of dermatologists enhances the validity of the data, this regional focus, along with the limited sample size, restricts the generalizability of the findings to the broader Finnish population. Furthermore, the data were collected at a single time point, so it was impossible to longitudinal assessment of skin diseases. The dataset also contained a high volume of missing values for certain skin-related variables, which limited the feasibility of applying more complex models. Consequently, we had to rely on simpler models and relationships to maintain the validity of our findings. Moreover, this was a cross-sectional study where external factors were not controlled, making it impossible to establish causal relationships. Therefore, it is recommended to use clinical trials study to investigate causal relationships or employ genetic methods such as Mendelian Randomization (MR) to explore the causal link between skin diseases and diabetes"

A discussion on the study limitations has been provided by the authors.

Can the authors please confirm if they adhered to any reporting guidelines, and provide a copy of the associated checklist?

Reviewer **2**

Name **Yunir, Em**

Affiliation **Universitas Indonesia Fakultas Kedokteran, Division of Endocrinology, Metabolism and Diabetes, Department of Internal Medicine**

Date **11-Feb-2026**

COI **None**

Dear Authors,

This study is interesting, but there are some comments to improve the manuscript:

A. Introduction

- "An estimated 537 million adults aged 20–79 years worldwide." It would be better to specify the year of the data

- It would be better to provide information on how long the diabetes has been present after identifying the skin health status

- I think the introduction is too long. It would be better to make it more concise

- "General approaches to variable selection fall into three main categories…" I think it's more suitable to put this explanation in the method section.

- "In the path model, we examined the direct relationships between selected skin health status and T2D". It would be better to move this sentence to the method section

- "The comprehensive data encompass health records, lifestyle factors, and demographic information, facilitating extensive research into various health outcomes, such as neurological development." I think the authors do not need to mention the variables that are not included in this study, such as neurogical development.

B. Method

- It would be better to state a cross-sectional study as the study design in the abstract and method section.

- Is the questionnaire different for subjects at ages 31 and 46? It would be better to state it in the manuscript.

- I think using metformin alone as an indicator of diabetes is a weakness. You may consider putting it in the limitation of the study.

- Evaluating the impact of diet at age 46 based on data collected at age 31 may problematic due to the long time period interval. I think it should be stated as a limitation of the study

C. Result

- “Among the study participants, the overall prevalence of T2D was 4.8%, with a significantly higher rate observed in men (5.6%) compared to women.” I can’t find this percentage in the table. It would be better to include the percentage in the table as well.

- It would be better to cite the number of the table if the authors mention the results

- “prevalence of tinea pedis was 27%, with a higher incidence in men than in women”. I think the table format should be improved, as it’s a bit confusing. The authors stated that there was a higher incidence of tinea pedis in women, but I couldn’t find it in the table. The table showed that the percentage of tinea pedis was 36.8% in men and 18.5% in women. Please improve all similar cases. You may consider creating a 100% total in a row rather than in a column

- I think variable selection is suitable to put in the method section

- “A total of 17 variables were selected using the ridge method, 15 variables with the lasso method, 12 through single covariate analysis, 18 with BSLM, and 15 with sparse step”. It would be better to recheck the number of variables in the text and in figure 2, as there were 15 dots of Ridge in the figure, whereas the authors stated that there were 17 variables

- “A total of 15 variables were initially selected using various methods.” It would be better to state that various methods.

- I think the explanation about path analysis is more appropriate to put in the method section rather than in the results.

- It would be better to add a narrative description of the figure 3 in the result section

- Figure 3 should not only report numerical values but also include corresponding symbol to improve clarity

- It would be better to provide an explanation for the uncommon variable, such as Café-au-lait-spot in the method section

- I think the format of figure 1 is somewhat confusing, as the final percentage (69.5%) seems to represent the response rate at the beginning (participants in 31-year-old)

D. Discussion

- “This study identified a significant direct association between T2D and pityriasis versicolor, psoriasis”. If the author stated T2D first, as in the sentence above, it looks like T2D as the exposure. Consistency in how the exposure and outcome are described would improve clarity.

- “This hypothesis has been addressed by spoendlin and colleagues, who conducted a population-based case–control analysis using UK-based General Practice Research Database to analyze the association between rosacea and T2D”. It would be better to capitalize the author’s name at the beginning. Moreover, it would be better to explain the results of that study

- It would be better to add the explanation on how hyperhidrosis may lead to diabetes. If the association is bidirectional, please explain in the manuscript

E. Conclusion

- It would be better to make the conclusion more concise

- The limitations of the study should be put at the end of the discussion section

It would be better to provide the funding details of this study

---

VERSION 1 - AUTHOR RESPONSE

Reviewer: 1
Prof. Zoe Kelson, University of Exeter
Comments to the Author:
This study applies variable selection methods to identify key dermatological factors linked to T2D and examines their direct and indirect associations using path analysis.

Reviewer comments:

"Of 47 skin-related variables, key predictors were selected using test-based and penalty-based methods." [Abstract]
Can these methods please be specified in the Abstract?

Answer:

Thank you for the suggestion. I have updated the Abstract as follows:

"Of 47 skin-related variables, key predictors were selected using test-based methods (univariate analysis, backward, forward, and stepwise logistic regression) and penalty-based/machine learning methods (Lasso, Ridge, minimax concave penalty, and Sparse Stepwise Regression)."

"We analyzed data from the Northern Finland Birth Cohort 1966 (NFBC1966), including 1,930 participants (1,036 women, 894 men) at ages 31 and 46"
and
"The cohort comprises 12138 live births from 1966, representing 96.3% of all births that year in Finland's two northernmost provinces, Oulu and Lapland. Data collection began during pregnancy, starting between the 10th and 16th gestational weeks, and has continued through multiple follow-up stages, including assessments at ages 1, 14, 31, and 46...
The current study aimed at including all participants with available data on skin health status and type 2 diabetes based on their participation to follow-ups organized when the participants were approximately 46 years (in 2012), while also utilizing covariate data from age 31 (collected in 1997). Following logistic and financial constraints, the clinical examination for dermatology diagnosis was only intended to participants living in the region of Oulu and was conducted at the University Hospital of Oulu. It included a total of 894 males and 1036 females (N = 1930)."
Can the authors please further comment on whether the analysed sample can be considered to be representative?

Answer:

Thank you for this important comment. The original NFBC1966 cohort is broadly representative of individuals born in 1966 in the two northernmost provinces of Finland. However, the analyzed sample in the present study (N = 1,930) was restricted to participants who attended the 46-year follow-up and underwent dermatological examination in the Oulu region. Due to this regional restriction and participation requirements, the analyzed sample cannot be considered fully representative of the entire original cohort or the broader Finnish population.

We have acknowledged this limitation in the Discussion section, where we note that the regional focus and relatively small sample size may limit the generalizability of the findings.

"T2D was defined based on oral glucose tolerance tests, self-reports, and Metformin prescriptions. Skin conditions were assessed through dermatologist diagnoses and questionnaires. "
Can the authors please acknowledge the potential for self-reporting bias?

Answer:

Thank you for the comment.

Regarding the definition of T2D, we used a combination of oral glucose tolerance test (OGTT) results, self-reported diagnoses, and Metformin prescription data. Since objective clinical measurements (OGTT) and medication records were included alongside self-reports, the likelihood of substantial self-reporting bias in the definition of T2D is minimized.

Concerning skin health status, nearly all skin-related variables were assessed through clinical examinations conducted by dermatologists. The only exceptions were *Using sunscreen inland*, *Using sunscreen abroad*, and *Psoriasis*, which included questionnaire-based information. Therefore, the potential for self-reporting bias is limited and applies only to these specific variables.

"To account for the long-term impact of diet, education, anxiety, and depression, we employed data from 31 ages in this study...
Information on poor quality sleep was collected by questionnaire at age 46 years. "
Can the authors please consider examining longitudinal data (i.e. at multiple ages) for changes over time?

Answer:

We appreciate the reviewer's comment. To address this point, we have assessed the long-term impact of the different variables, and the corresponding data are presented in the tables below.

Education	31-year-old N	46-year-old N
less than 9 years of comprehensive school	529	200
Comprehensive school	5912	4675
Highly educated	1133	1816
Missing	4564	5447
Mean score	2.08	2.24
(McNemar test) p-value < 0.001		

Over the past 15 years, the education distribution has changed. the number of individuals with less than 9 years of comprehensive school decreased by 329, the number of those with a comprehensive school education decreased by 1,237, while the number of highly educated individuals increased by 683.

Diet	31-year-old N (%)	46-year-old N (%)
Unhealthy (0)	7451 (87.8%)	5872 (93.6%)
Healthy (1)	1035 (12.2%)	400 (6.4%)
(McNemar test) p-value < 0.001		

The diet variable also changed significantly over time, with the percentage of healthy diets decreasing from 12.2% to 6.4%, indicating that dietary conditions have worsened.

Variable	Age 31 Mean (sd)	Age 46 Mean (sd)	(McNemar test) p-value
Anxiety	1.313 (0.31)	1.298 (0.32)	0.1153
Depression	1.354 (0.35)	1.368 (0.39)	< 0.001

The mean anxiety score decreased over time, but this change was not statistically significant. The mean depression score increased by 0.01.

"Chi-square tests were used to compare categorical variables between sex groups and between health status groups as presented... including Single covariate techniques based on p-values, as well as backward stepwise logistic regression (BSLR), forward stepwise logistic regression (FSLR), and stepwise logistic regression (SLR). Additionally, we utilized machine learning techniques, specifically least absolute shrinkage and selection operator regression (Lasso), Ridge regression, minimax convex penalty (MCP), and Sparse Stepwise Regression. The degree of agreement between the results of various methods was assessed using Cohen's Kappa index"
A range of appropriate analytical methodologies have been applied.
Can these methods please be further described in the Statistical Analysis section, including specifying the 'single covariate techniques' referred to here?

Answer:

Thank you for the comment, The term “single covariate techniques” refers to univariate analyses, which were performed separately for each variable, with every predictor assessed individually in relation to the outcome.

"Following the selection of skin health status, we employed path analysis to examine both direct and indirect associations, while controlling for the effects of sex, body mass index (BMI), diet, education, depression, and anxiety (Supplementary Fig. 1)"
and
"Table 1 Descriptive characteristics of the study population"
Can other factors please be reported and controlled for, including race or ethnicity and medications for instance?

Answer:

Thank you for the suggestion, We included all relevant potential confounders in the multivariable models to minimize residual confounding.

Regarding race or ethnicity, this variable was not included because the study population consisted exclusively of Finnish participants from the Northern Finland Birth Cohort 1966, and therefore there was no meaningful variation in race or ethnicity within the sample.

"A total of 15 variables were initially selected using various methods. From these, to ensure proper control over Type I and Type II errors, we filtered results with significant p-values in the single covariate analysis."
Can the authors please also demonstrate that the explored variables are also clinically meaningful?

Answer:

Thank you for the comment. All variables included in the study were clinically meaningful, as the study was conducted in collaboration with a dermatologist. Additionally, we reviewed the existing literature to ensure that the selected variables are relevant and supported by previous research.

"It is worth noting that the multicollinearity of the selected variables was assessed by the variance inflation factor (VIF) (Supplementary Table3)"
Can this methodology assessing collinearity please also be specified in the Statistical Analysis section, with an interpretation of outcomes summarised in the Result text?

Answer:

Thank you for the valuable suggestion.

We have updated the manuscript as follows:

The multicollinearity of the selected variables was assessed using the variance inflation factor (VIF), with a maximum VIF exceeding 10 indicating potential influence of multicollinearity on the least squares estimates [19].

The maximum VIF observed was 2.38 (for Anxiety and Depression), and the mean VIF across all variables was 1.21, both well below the commonly used threshold of 10. This indicates that multicollinearity did not pose a concern in the model (Supplementary Table 3)

"Model fit was evaluated using several indices. The RMSEA (0.060) and SRMR (0.045) were both below 0.08 and the relative Chi-square (CMIN/DF) was calculated as 5.89, which is <6."
Can the assessment of model performance metrics please be specified in the Statistical Analysis section?

Thank you for the comment.

We have updated the manuscript as follows:

Model fit was evaluated using the root mean square error of approximation (RMSEA), the standardized root mean square residual (SRMR), and the relative chi-square (Chi-square minimum discrepancy (CMIN)/ Degrees of Freedom (DF)). Values of RMSEA and SRMR below 0.08 and CMIN/DF below 5 were considered indicative of acceptable model fit, based on established recommendations in structural equation modeling literature [20].

"Figure 1 Flow chart demonstrating the process used to select the study participants"
Can the authors please further comment on missing data?
How much was missing for each variable or factor, and can it be considered to be missing at random?
Can the authors please clarify how missing data was handled in the analysis?

Answer:

Thank you for the comment. In the original 31-year follow-up cohort, 4,988 individuals (41.1%) did not participate in the 46-year follow-up conducted approximately 15 years later. Given the long follow-up interval, this degree of attrition is expected in longitudinal cohort studies and is unlikely to be systematic in nature. Therefore, this loss to follow-up can reasonably be considered as missing at random.

After applying the eligibility criteria for the skin data, a total of 1,930 participants were included in the final analytical sample. Thus, the final sample size used in this project was 1,930 participants, and Figure 1 (flow chart) has been revised accordingly.

Regarding missing data at the variable level, the proportion of missing values for individual variables was low. The number of missing observations for each variable is shown in the table below, with detailed information also provided in **Supplementary Table 1**.

Missing data were handled using listwise deletion. Participants with missing values in any of the variables included in a given model were excluded from that specific analysis.

Table 1 Descriptive Characteristics of the Study Population and Extent of Missing Data

Variable	Category	Overal	Observed value (N)	Missing value (N)	Missing value (%)	Total (N)
Tinea pedis	No	1392 (73.0 %)	1906	24	1.24	1930
	Yes	514 (27.0 %)				
Hyperhidrosis	No	1864 (97.8 %)	1906	24	1.24	1930
	Yes	42 (2.2 %)				
Café-au-lait spots	No	1668 (87.5 %)	1906	24	1.24	1930
	Yes	238 (12.5 %)				
Pigmented nevus	No	1685 (88.4 %)	1906	24	1.24	1930
	Yes	221 (11.6 %)				
Lentigo senilis	No	1650 (86.6 %)	1906	24	1.24	1930
	Yes	256 (13.4 %)				

Psoriasis skin symptoms	No	1862 (97.9 %)	1902	28	1.45	1930
	Yes	40 (2.1 %)				
Psoriasis nail symptoms	No	1889 (99.4 %)	1901	29	1.5	1930
	Yes	12 (0.6 %)				
Psoriasis joint symptoms	No	1888 (99.3 %)	1901	29	1.5	1930
	Yes	13 (0.7 %)				
Psoriasis	No	6575 (97.7 %)	6731	419	21.71	7150
	Yes	156 (2.3 %)				
Rosacea	No	1614 (84.8 %)	1904	26	1.35	1930
	Yes	290 (15.2 %)				
Stasis dermatitis	No	1890 (99.2 %)	1905	25	1.3	1930
	Yes	15 (0.8 %)				
Bluenevus	No	1880 (98.6 %)	1906	24	1.24	1930
	Yes	26 (1.4 %)				
Nevus spilus	No	1817 (95.3 %)	1906	24	1.24	1930
	Yes	89 (4.7 %)				
Halo nevus	No	1891 (99.2 %)	1906	24	1.24	1930
	Yes	15 (0.8 %)				
Dermatofibroma	No	1481 (77.7 %)	1906	24	1.24	1930
	Yes	425 (22.3 %)				
Cherry angioma	No	759 (39.8 %)	1905	25	1.3	1930
	Yes	1146 (60.2 %)				
Cheloid	No	1857 (97.4 %)	1906	24	1.24	1930
	Yes	49 (2.6 %)				
Seborrheic keratosis	No	1061 (55.7 %)	1906	24	1.24	1930
	Yes	845 (44.3 %)				
Lipoma	No	1845 (96.8 %)	1906	24	1.24	1930
	Yes	61 (3.2 %)				
Congenital nevus	No	1828 (95.9 %)	1906	24	1.24	1930
	Yes	78 (4.1 %)				
Nevus flammeus	No	1419 (74.4 %)	1906	24	1.24	1930
	Yes	487 (25.6 %)				
Actinic keratosis	No	1894 (99.4 %)	1906	24	1.24	1930
	Yes	12 (0.6 %)				

Atopic dermatitis	No	1813 (95.2 %)	1905	25	1.3	1930
	Yes	92 (4.8 %)				
Hand eczema	No	1736 (91.1 %)	1905	25	1.3	1930
	Yes	169 (8.9 %)				
Seborrheic dermatitis	No	1765 (92.7 %)	1905	25	1.3	1930
	Yes	140 (7.3 %)				
Nummular dermatitis	No	1869 (98.1 %)	1905	25	1.3	1930
	Yes	36 (1.9 %)				
Infectious eczema	No	1878 (98.6 %)	1905	25	1.3	1930
	Yes	27 (1.4 %)				
Contact dermatitis	No	1887 (99.1 %)	1905	25	1.3	1930
	Yes	18 (0.9 %)				
Neurodermatitis	No	1871 (98.2 %)	1905	25	1.3	1930
	Yes	34 (1.8 %)				
Lichen planus	No	1893 (99.3 %)	1906	24	1.24	1930
	Yes	13 (0.7 %)				
Dermatitis herpetiformis	No	1900 (99.7 %)	1906	24	1.24	1930
	Yes	6 (0.3 %)				
Vitiligo	No	1874 (98.3 %)	1906	24	1.24	1930
	Yes	32 (1.7 %)				
Urticaria	No	1896 (99.5 %)	1906	24	1.24	1930
	Yes	10 (0.5 %)				
Acne	No	1755 (92.1 %)	1905	25	1.3	1930
	Yes	150 (7.9 %)				
Perioral dermatitis	No	1888 (99.3 %)	1902	28	1.45	1930
	Yes	14 (0.7 %)				
Acne scars	No	1716 (90.2 %)	1902	28	1.45	1930
	Yes	186 (9.8 %)				
Pityriasis versicolor	No	1869 (98.1 %)	1906	24	1.24	1930
	Yes	37 (1.9 %)				
Onychomycosis	No	1726 (90.6 %)	1906	24	1.24	1930
	Yes	180 (9.4 %)				
Tinea corporis	No	1898 (99.6 %)	1906	24	1.24	1930
	Yes	8 (0.4 %)				
Folliculitis	No	1790 (93.9 %)	1906	24	1.24	1930
	Yes	116 (6.1 %)				
Plantar warts	No	1732 (90.9 %)	1906	24	1.24	1930
	Yes	174 (9.1 %)				
Hand warts	No	1855 (97.3 %)	1906	24	1.24	1930

	Yes	51 (2.7 %)				
Alopecia areata	No	1899 (99.6 %)	1906	24	1.24	1930
	Yes	7 (0.4 %)				
Male pattern baldness	No	1291 (67.7 %)	1906	24	1.24	1930
	Yes	615 (32.3 %)				
Other skin disease	No	1595 (90.2 %)	1768	162	8.39	1930
	Yes	168 (88.4 %)				
	Perhaps	5 (11.6 %)				
Using sunscreen inland	Not at all	1068 (15.9 %)	6727	423	21.92	7150
	Sometimes	3170 (47.1 %)				
	Reqularly	2210 (32.9 %)				
	I do not spend time in the sun	279 (4.1 %)				
Using sunscreen in abroad	Not at all	960 (15.1 %)	6345	805	41.71	7150
	Sometimes	574 (9.0 %)				
	Reqularly	4079 (64.3 %)				
	I do not spend time in the sun	732 (11.5 %)				

"However, these studies have faced limitations. In the NFBC1966 study, data on skin conditions were collected by dermatologists at the age of 46 for individuals who were readily accessible, primarily residing in northern Finland. While the involvement of dermatologists enhances the validity of the data, this regional focus, along with the limited sample size, restricts the generalizability of the findings to the broader Finnish population. Furthermore, the data were collected at a single time point, so it was impossible to longitudinal assessment of skin diseases. The dataset also contained a high volume of missing values for certain skin-related variables, which limited the feasibility of applying more complex models. Consequently, we had to rely on simpler models and relationships to maintain the validity of our findings. Moreover, this was a cross-sectional study where external factors were not controlled, making it impossible to establish causal relationships. Therefore, it is recommended to use clinical trials study to investigate causal relationships or employ genetic methods such as Mendelian Randomization (MR) to explore the causal link between skin diseases and diabetes"
A discussion on the study limitations has been provided by the authors.

Can the authors please confirm if they adhered to any reporting guidelines, and provide a copy of the associated checklist?
STROBE CHECKLIST

Thank you for your suggestion. The completed STROBE checklist has been provided as a separate file titled "STROBE-checklist-bmjopen-2025-109709".

Reviewer: 2
Dr. Em Yunir, Universitas Indonesia Fakultas Kedokteran, Rumah Sakit Umum Pusat Nasional Dr Cipto Mangunkusumo
Comments to the Author:
Dear Authors,
This study is interesting, but there are some comments to improve the manuscript:

A. Introduction
- "An estimated 537 million adults aged 20–79 years worldwide." It would be better to specify the year of the data

Answer:

We thank the editor for this valuable comment. Revisions were made accordingly in the main text:

An estimated 537 million adults aged 20–79 years worldwide (10.5% of all adults in this age group) have diabetes **in 2021**

- It would be better to provide information on how long the diabetes has been present after identifying the skin health status

Answer:

Thank you for this important comment. In our study, both skin assessments and diabetes status were collected at the same time point during the clinical examination. Therefore, it is not possible to determine the duration of diabetes in relation to the observed skin findings.

- I think the introduction is too long. It would be better to make it more concise

Answer:

Thank you for the comment. We have revised the Introduction by removing the sections related to variable selection and path analysis, which have now been moved to the Methods section. As a result, the Introduction has been shortened and made more concise.

- "General approaches to variable selection fall into three main categories…" I think it's more suitable to put this explanation in the method section.

Answer:

Thank you for the suggestion. We have moved this explanation to the Methods section accordingly.

- "In the path model, we examined the direct relationships between selected skin health status and T2D". It would be better to move this sentence to the method section

Answer:

Thank you for the suggestion. We have moved this sentence to the Methods section accordingly.

- “The comprehensive data encompass health records, lifestyle factors, and demographic information, facilitating extensive research into various health outcomes, such as neurological development.” I think the authors do not need to mention the variables that are not included in this study, such as neurogical development.

Answer:

Thank you for the comment. We agree and have removed this part from the manuscript.

B. Method

- It would be better to state a cross-sectional study as the study design in the abstract and method section.

Answer:

Thank you for this suggestion. While the outcomes and exposures were assessed at the age of 46 years, the covariates were derived from data collected at earlier follow-ups (e.g., age 31 years). Therefore, the study design is not purely cross-sectional, as it incorporates longitudinal information. We have clarified this in the Methods section to better describe the study design.

- Is the questionnaire different for subjects at ages 31 and 46? It would be better to state it in the manuscript.

Answer:

The background and lifestyle questionnaires were largely the same at both ages, with only minor changes at age 46. However, Not all questionnaires and clinical data were collected identically at both time points.

- I think using metformin alone as an indicator of diabetes is a weakness. You may consider putting it in the limitation of the study.

Answer:

We thank the reviewer for the comment. For clarification, in our study, we did not rely on Metformin use alone to define diabetes. T2D was defined based on a combination of oral glucose tolerance tests, self-reports, and Metformin prescriptions.

- Evaluating the impact of diet at age 46 based on data collected at age 31 may problematic due to the long time period interval. I think it should be stated as a limitation of the study

Answer:

Thank you for the important comment. We also had diet data available at age 46; however, our aim was to investigate the potential long-term effects of this factor. Therefore, we used the data collected at age 31.

C. Result

- "Among the study participants, the overall prevalence of T2D was 4.8%, with a significantly higher rate observed in men (5.6%) compared to women." I can't find this percentage in the table. It would be better to include the percentage in the table as well.

Answer:

Thank you for the comment, This information is already included in Table 1 (Descriptive characteristics of the study population) as the final variable.

Hyperhidrosis	Yes	42 (2.2 %)	32 (3.6 %)	10 (1.0 %)	P<0.001
Type 2 diabetes	No	6660 (95.2 %)	3027 (94.4 %)	3633 (95.9 %)	0.004
	Yes	336 (4.8 %)	180 (5.6 %)	156 (4.1 %)	

P-value[a] for differences between male and female. P-value[b] for differences between people with diabetes and healthy people

- It would be better to cite the number of the table if the authors mention the results

Answer:

Thank you for the suggestion. This has been done accordingly.

- "prevalence of tinea pedis was 27%, with a higher incidence in men than in women". I think the table format should be improved, as it's a bit confusing. The authors stated that there was a higher incidence of tinea pedis in women, but I couldn't find it in the table. The table showed that the percentage of tinea pedis was 36.8% in men and 18.5% in women. Please improve all similar cases. You may consider creating a 100% total in a row rather than in a column

Answer:

We thank the reviewer for the comment. By the sentence "the prevalence of tinea pedis was 27%, with a higher incidence in men than in women", we intended to indicate that tinea pedis was more prevalent among men than women. This is reflected in Table 1, which shows a higher percentage in men (36.8%) compared to women (18.5%).

To avoid any potential misunderstanding, the manuscript will be reviewed by a proficient English speaker and the relevant sentence will be revised for clarity.

We respectfully disagree with the suggestion to present percentages with a 100% total per row, as this would change the interpretation of the estimates and would no longer reflect the sex-specific prevalence of tinea pedis in men and women.

- I think variable selection is suitable to put in the method section

Answer:

Thank you for the suggestion. All information related to variable selection has been transferred to the Methods section accordingly.

- “A total of 17 variables were selected using the ridge method, 15 variables with the lasso method, 12 through single covariate analysis, 18 with BSLM, and 15 with sparse step”. It would be better to recheck the number of variables in the text and in figure 2, as there were 15 dots of Ridge in the figure, whereas the authors stated that there were 17 variables

Answer:

Thank you for your comment. You are correct that a typographical error occurred in the initial text. We have double-checked the numbers, and the counts have now been corrected: 17 variables were selected using the Ridge method, 15 variables with the Lasso method, 11 through single covariate (univariate) analysis, 18 with BSLR, 19 with MCP and 15 with Sparse Step. The figure and the text are now consistent.

We have updated the manuscript as follows:

The variable “other skin disease” was eligible for inclusion in the final list of variables based on our selection strategy but was excluded due to its limited informativeness, likely reflecting its broad and non-specific definition during data collection. This highlights the importance of precise variable definitions, as poorly defined variables may reduce analytical utility.

- “A total of 15 variables were initially selected using various methods.” It would be better to state that various methods.

Answer:

Thank you so much for this valuable comment. We have revised the sentence to make it clearer and more understandable.

We have updated the manuscript as follows:

A total of 15 variables were initially selected, each identified by a minimum of 4 different selection methods

- I think the explanation about path analysis is more appropriate to put in the method section rather than in the results.

Answer:

Thank you for the comment. You are correct, and we have moved the explanation about path analysis to the Methods section.

- It would be better to add a narrative description of the figure 3 in the result section

Answer:

Thank you for the suggestion. We have provided a concise narrative description of Figure 3, including a summary of the sample size and model goodness-of-fit values. Detailed information on these aspects is already reported in the Methods and Results sections. We believe adding further narrative description of this figure would largely duplicate information already reported and may lead to overlap with existing tables and text.

- Figure 3 should not only report numerical values but also include corresponding symbol to improve clarity

Answer:

Thank you for the comment. We have clarified in the figure legend that the values shown in the plot represent the path (β) coefficients.

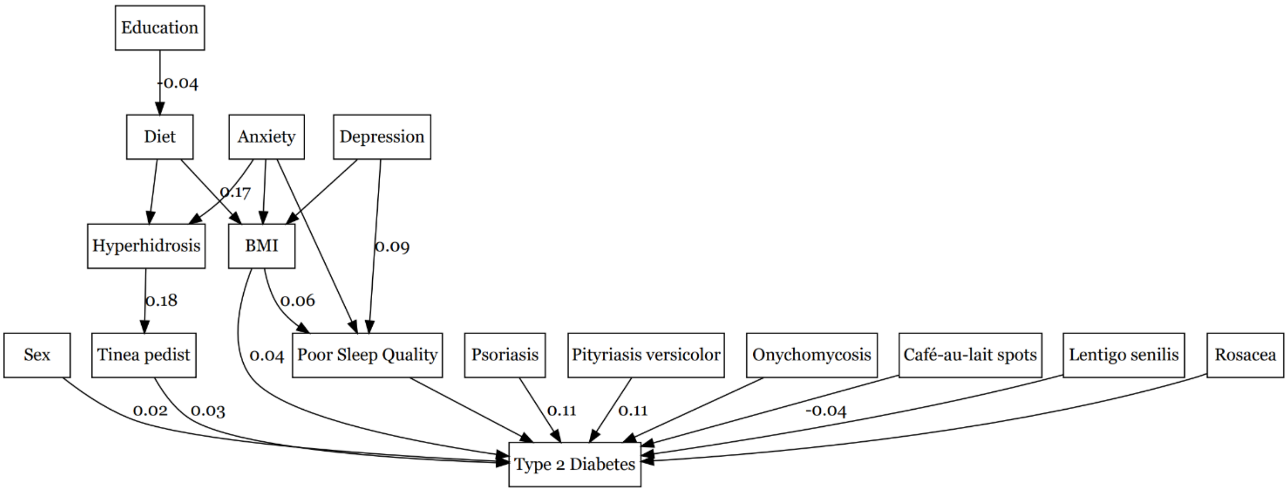

Figure 3 The path analysis was performed in 1930 individuals aged 46 years. All depicted arrows represent statistically significant associations (Beta coefficients). Model fit values: SRMR = 0.045, RMSEA = 0.060.

- It would be better to provide an explanation for the uncommon variable, such as Café-au-lait-spot in the method section

Answer:

Thank you for this valuable comment. Fourteen different benign skin tumors, including café-au-lait spots, were assessed in our study by dermatologists. Café-au-lait spots are not uncommon; in our study population, they were observed in approximately 12% of participants. This is consistent with previous literature, which suggests that around 10–20% of the general population have at least one café-au-lait spot.

- I think the format of figure 1 is somewhat confusing, as the final percentage (69.5%) seems to represent the response rate at the beginning (participants in 31-year-old)

Answer:

Thank you for the comment. The previous flow chart contained an error. We have revised it to reflect the final sample of 1,930 participants, and the updated chart should now be clear and not confusing.

D. Discussion

- “This study identified a significant direct association between T2D and pityriasis versicolor, psoriasis”. If the author stated T2D first, as in the sentence above, it looks like T2D as the exposure. Consistency in how the exposure and outcome are described would improve clarity.

Answer:

Thank you for your comment. We will revise this for clarity and consistency.

We have updated the manuscript as follows:

This study identified a significant direct association of pityriasis versicolor, psoriasis, and tinea pedis with T2D, the corresponding odds ratios were 1.115 (95% CI: 1.03, 1.208), 1.115 (95% CI: 1.038, 1.198), and 1.033 (95% CI: 1.006, 1.059), respectively.

- “This hypothesis has been addressed by spoendlin and colleagues, who conducted a population-based case–control analysis using UK-based General Practice Research Database to analyze the association between rosacea and T2D”. It would be better to capitalize the author’s name at the beginning. Moreover, it would be better to explain the results of that study

Answer:

Thank you for your comment. The author’s name has been capitalized and the study results have been added.

We have updated the manuscript as follows:

This hypothesis has been addressed by Spoendlin and colleagues, who conducted a population-based case–control analysis using UK-based General Practice Research Database to analyze the association between rosacea and T2D, reporting a significantly reduced risk of rosacea among individuals with diabetes mellitus (OR = 0.80, 95% CI 0.74–0.85). This inverse association was more pronounced in individuals with higher HbA1c levels and longer diabetes duration, suggesting that advanced stages of diabetes may be linked to a lower likelihood of developing rosacea [32].

- It would be better to add the explanation on how hyperhidrosis may lead to diabetes. If the association is bidirectional, please explain in the manuscript

Answer:

Thank you for your comment. In our study, we did not observe a bidirectional association between hyperhidrosis and T2D. Instead, the findings suggest that hyperhidrosis may be indirectly associated with T2D through its relationship with tinea pedis

E. Conclusion
- It would be better to make the conclusion more concise

Answer:

Thank you for the comment. We have removed the implications and limitations from the Conclusion section, making it more concise and focused.

- The limitations of the study should be put at the end of the discussion section

Answer:

Thank you for the suggestion. The limitations of the study have been moved to the end of the Discussion section.

It would be better to provide the funding details of this study

Answer:

Thank you for the suggestion.

We have updated the manuscript as follows:

Funding

This work is funded by Infotech Oulu, University of Oulu (M.N.). **Research Council of Finland**

Profi8 funding (decision number 365202) and BF (No. 4417/31/2025) (N.A.).

Reviewer: 1
If you have selected 'Yes' above, please provide details of any competing interests.: Not applicable

Reviewer: 2
If you have selected 'Yes' above, please provide details of any competing interests.: Not applicable

VERSION 2 - REVIEW

**Reviewer**	**1**
**Name**	**Kelson, Zoe**
**Affiliation**	**University of Exeter, Mathematics**
**Date**	**13-Apr-2026**

COI

Thanks to the authors for their responses and revisions. The following reviewer requests are raised:

Author response:

"To address this point, we have assessed the long-term impact of the different variables, and the corresponding data are presented in the tables below... "

Reviewer request:

Can this response to reviewer please be further incorporated into the main article?

Can changes over time please be accounted for in the analyses?

Author response:

"The term “single covariate techniques” refers to univariate analyses, which were performed separately for each variable, with every predictor assessed individually in relation to the outcome."

Reviewer request:

Can the authors please specify the univariate analyses applied here?

Author response:

"In the original 31-year follow-up cohort, 4,988 individuals (41.1%) did not participate in the 46-year follow-up conducted approximately 15 years later. Given the long follow-up interval, this degree of attrition is expected in longitudinal cohort studies and is unlikely to be systematic in nature. Therefore, this loss to follow-up can reasonably be considered as missing at random."

Reviewer request:

Can the authors please assess whether loss-to-follow-up has left a representative sample (e.g. were the 4988 individuals demographically and characteristically similar to those that did respond)?

Author response:

"Regarding missing data at the variable level, the proportion of missing values for individual variables was low. The number of missing observations for each variable is shown in the table below, with detailed information also provided in Supplementary Table 1 . Missing data were handled using listwise deletion. Participants with missing values in any of the variables included in a given model were excluded from that specific analysis"

Reviewer request:

Can the authors please consider undertaking a sensitivity analysis on the approach taken for handling missing data, in particular when variables are >5% missing?

VERSION 2 - AUTHOR RESPONSE

Section 2: Reviewer comment
Reviewer: 1
Prof. Zoe Kelson, University of Exeter
Comments to the Author:
Thanks to the authors for their responses and revisions. The following reviewer requests are raised:

Author response:
"To address this point, we have assessed the long-term impact of the different variables, and the corresponding data are presented in the tables below... "

Reviewer request 1: this request has two questions
Q1: Can this response to reviewer please be further incorporated into the main article?
Q2: Can changes over time please be accounted for in the analyses?

Our Answer to Q1:

We thank the reviewer for this suggestion. We are providing below the information that is now included in the manuscript and as Supplementary Material.

"In addition, covariates were compared over time, and McNemar's test and paired t-tests were used (Supplementary Table S3)." line 157:158

Table S3 Distribution of study covariates at 31-year and 46-year follow-ups

Variable	31-year follow-up	46-year follow-up	P-value
**Education, N (%)**			<0.001
<9 years	72 (4.71 %)	38 (2.08 %)	
Comprehensive school	1221 (79.91 %)	1310 (71.70 %)	
Highly educated	235 (15.38 %)	479 (26.22 %)	
Missing, N	378	79	
**Diet, N (%)**			<0.001
Unhealthy	1518 (88.51 %)	1603 (93.90 %)	
Healthy	197 (11.49 %)	401 (6.10 %)	
Missing, N	191	199	
**Anxiety, Mean (sd)**	1.29 (0.28)	1.29 (0.30)	0.8113
Missing, N	183	112	
**Depression, Mean (sd)**	1.32 (0.32)	1.35 (0.37)	0.0019
Missing, N	203	112	

Our Answer to Q2:

We considered the following variables to be included in the path analysis model to account for their potential influence on the relation between the selected skin variables (skin health status) and the outcome (T2D). We assessed the cross-sectional associations of body mass index (BMI) and sleep quality measured at the 46-year follow-up, along with the long-term effects of diet, education, anxiety, and depression measured at the 31-year follow-up. We indeed took a conceptually driven approach. Variables that are typically more stable over time, such as diet, anxiety, depression, and education, were modelled using data from age 31 to represent longer-term or distal influences. In contrast, more proximal and potentially time-sensitive factors, such as MBI and sleep quality, were derived from the 46-year data to better reflect the closer temporal context of the outcomes.

These latter variables were taken from the 31-year assessment due to their relative stability over time, allowing us to capture potential lasting effects. The distributions of these covariates were compared across time points using McNemar's tests or paired t-tests (Supplementary Table S3). Although significant differences between the two follow-up periods were observed, these likely reflect natural trends (e.g., changes in educational attainment and increasing prevalence of anxiety and depression over time). To reduce the risk of multicollinearity arising from correlated repeated measures, we included covariates variables from only a single time point in the final path model.

Table correlation between covariates in 31- and 46-year-old

	**Education 31**	**Diet 31**	**Anxiety 31**	**Depression 31**
**Education 46**	**0.67**			
**Diet 46**		**0.20**		
**Anxiety 46**			**0.40**	
**Depression 46**				**0.44**

Furthermore, we have revised the path analysis figure to improve its clarity and facilitate interpretation of the model.

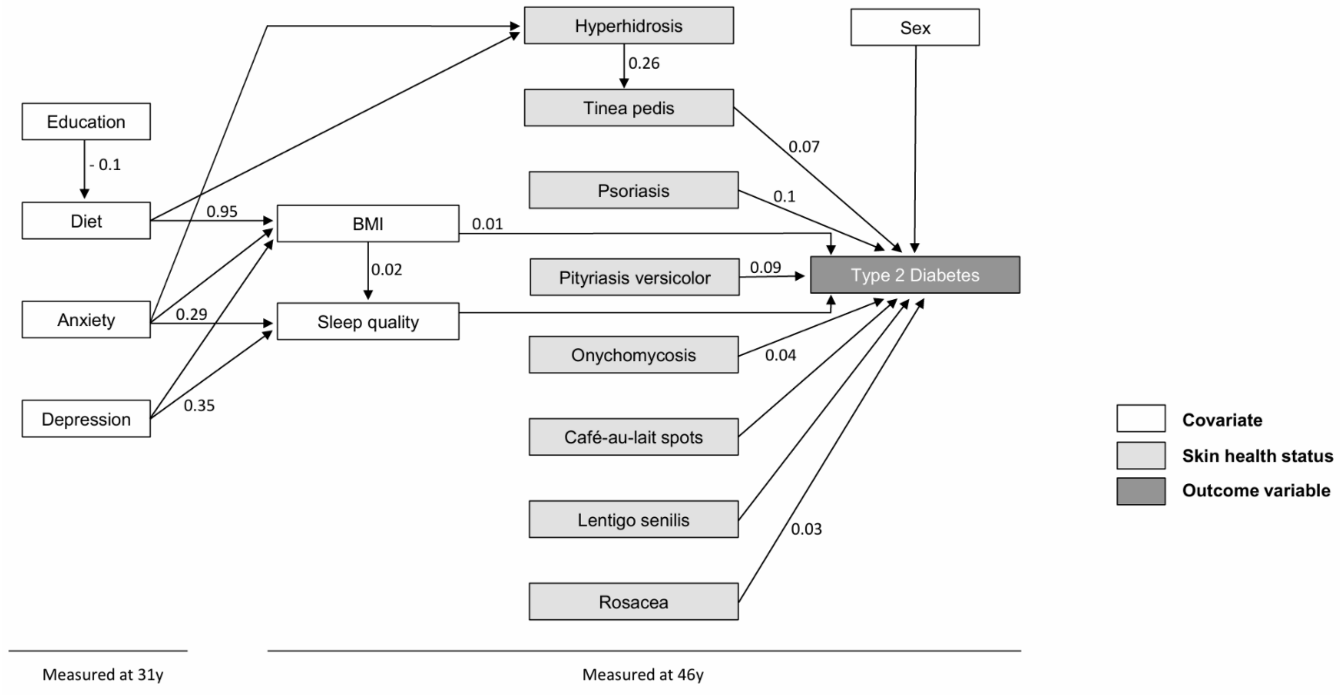

Figure 3 The path analysis was performed in 1906 individuals aged 46 years. All depicted arrows represent statistically significant associations (Beta coefficients). Model fit values: SRMR = 0.031, RMSEA = 0.040.

Author response:
"The term “single covariate techniques” refers to univariate analyses, which were performed separately for each variable, with every predictor assessed individually in relation to the outcome."

Reviewer request 2:
Can the authors please specify the univariate analyses applied here?

Our Answer:

We thank the reviewer for this clarification. The univariate analyses refer to univariate logistic regression models, where each predictor was entered separately in relation to the outcome without adjustment for other covariates. This clarification has now been added to the manuscript.

“In this study, we employed a variety of methods to identify the most appropriate variables for path analysis modelling, including a univariate logistic regression analysis based on p-values, as well as a backward stepwise logistic regression (BSLR), forward stepwise logistic regression (FSLR), and stepwise logistic regression (SLR).” Lines 174-177

Author response:
"In the original 31-year follow-up cohort, 4,988 individuals (41.1%) did not participate in the 46-year follow-up conducted approximately 15 years later. Given the long follow-up interval, this degree of

attrition is expected in longitudinal cohort studies and is unlikely to be systematic in nature. Therefore, this loss to follow-up can reasonably be considered as missing at random."

Reviewer request 3:
Can the authors please assess whether loss-to-follow-up has left a representative sample (e.g. were the 4988 individuals demographically and characteristically similar to those that did respond)?

Our Answer:

We thank the reviewer for this important comment. As reported in the cohort profile of the Northern Finland Birth Cohort 1966 **[Nordström, T., Miettunen, J., Auvinen, J., Ala-Mursula, L., Keinänen-Kiukaanniemi, S., Veijola, J., Järvelin, M. R., Sebert, S., & Männikkö, M. (2022). Cohort Profile: 46 years of follow-up of the Northern Finland Birth Cohort 1966 (NFBC1966).** ***International journal of epidemiology*****,** ***50*****(6), 1786–1787j. https://doi.org/10.1093/ije/dyab109]**.

In 2012 at the age of 46 years, all cohort members who were alive and living in Finland with a known address (n = 10,331) were invited to participate in a large health examination consisting of questionnaires and clinical examinations. The reduction from the original cohort size (n = 12,138 at 31-year-old to n = 10,331 eligible at the 46-year follow-up) is explained by deaths, emigration, and individuals with unknown or invalid addresses over the follow-up period.

The cohort profile further reports that participation in adult follow-ups has shown a somewhat selective pattern over time. Participants in later follow-ups were more likely to be women, employed, married, and to have higher socioeconomic and educational status compared with non-participants. Participation rates in adulthood have ranged from approximately 69–75% for questionnaires and 55–71% for clinical examinations. Although attrition over time is substantial (including 41.1% non-participation between the 31- and 46-year follow-ups), these differences are mainly related to sociodemographic factors such as sex, education, and socioeconomic status. Importantly, no major systematic differences in key health-related characteristics have been reported that would preclude the use of the remaining cohort for epidemiological analyses.

sex	Participants at age 31 who participated in the 46-year follow-upN (%)	Participants at age 31 who not participated in 46 follow-up N (%)	p value
male	3280 (46 %)	2935 (58 %)	2.2e-16
female	3835 (54 %)	2086 (42 %)	

Females were more likely to participate in the 46 year follow-up study than males. Among those who participated, 54% were female and 46% were male.

Education	Participants at age 31 who Participated in 46 follow-up N (%)	Participants at age 31 who Not participated in 46 follow-up N (%)	p value
less than 9 years of comprehensive school	306 (5.5%)	223 (11.1%)	2.2e-16
Comprehensive school	4359 (78.4%)	1553 (77.2%)	
High educated	898 (16.1%)	235 (11.7%)	

Individuals with higher education were more likely to stay in the study. 16.1% of the participants were "High educated" compared to only 11.7% in the non-participant group. On the other hand, those with less than 9 years of school were twice as likely to drop out (11.1% among non-participants vs. 5.5% among participants).

Diet	Participants at age 31 who Participated in 46 follow-up N (%)	Participants at age 31 who Not participated in 46 follow-up N (%)	p value
Unhealthy	5522 (88.8%)	1929 (85.0%)	1.852e-06
Healthy	694 (11.2%)	341 (15.0%)	

individuals with an unhealthy diet had a slightly higher participation rate (88.8%) than those with a healthy diet (11.2%). Among those who did not participate, 15.0% had a healthy diet.

variable	Participants at age 31 who Participated in 46 follow-up	Participants at age 31 who Not participated in 46 follow-up	p value
Anxiety (mean (sd))	1.304 (0.299)	1.339 (0.342)	1.563e-05
Depression (mean (sd))	1.345 (0.345)	1.382 (0.377)	3.93e-05

Individuals who did not participate had higher anxiety levels (mean = 1.339, SD = 0.342) compared to those who participated in the 46-year follow-up (mean = 1.304, SD = 0.299).

Similar to anxiety, those who did not participate in the follow-up exhibited higher baseline depression levels (mean = 1.382, SD = 0.377) than those who remained in the study (mean = 1.345, SD = 0.345).

We have added this point to the Limitations section. “In addition, attrition and loss to follow-up are common in longitudinal studies. In the NFBC1966 cohort, differences in characteristics were observed between participants who participated in the 46-year follow-up and those who did not.

These differences suggest the possibility of differential attrition, which may introduce selection bias and should be considered when interpreting the findings." line 357:360

The corresponding tables have also been added to the Supplementary material.

"To assess potential selection bias arising from participant attrition between the two follow-ups, we compared the baseline covariate distributions at the 31-year follow-up between participants who attended the 46-year follow-up and those who did not; the results are presented in Supplementary Table S2." line 149:152

Table S2 Distribution of study covariates at 31-year who participated in the 46-year follow-up and those who did not

**Variable**	**Participated in the 46y**	**Not participated**	**P-value**
**Sex**			<0.001
Male	3280 (46 %)	2935 (58 %)	
Female	3835 (54 %)	2086 (42 %)	
**Education, N (%)**			<0.001
<9 years	306 (5.5%)	223 (11.1%)	
Comprehensive school	4359 (78.4%)	1553 (77.2%)	
Highly educated	898 (16.1%)	235 (11.7%)	
**Diet, N (%)**			<0.001
Unhealthy	5522 (88.8%)	1929 (85.0%)	
Healthy	694 (11.2%)	341 (15.0%)	
**Anxiety, Mean (sd)**	1.304 (0.299)	1.339 (0.342)	<0.001
**Depression, Mean (sd)**	1.345 (0.345)	1.382 (0.377)	<0.001

Author response:
"Regarding missing data at the variable level, the proportion of missing values for individual variables was low. The number of missing observations for each variable is shown in the table below, with detailed information also provided in Supplementary Table 1. Missing data were handled using listwise deletion. Participants with missing values in any of the variables included in a given model were excluded from that specific analysis"

Reviewer request 4: Can the authors please consider undertaking a sensitivity analysis on the approach taken for handling missing data in particular when variables are >5% missing?

Our Answer:

Thank you very much for this valuable suggestion. We have carefully examined the data, and the results of the imputation are presented below.

24 individuals had completely missing data for all skin-related variables. We considered that imputing individuals with entirely missing skin data would be equivalent to data fabrication, as no observed information was available for these variables. In addition, including them in the imputation model could introduce instability and potentially compromise the validity of the dataset. Therefore, we excluded these individuals from the study and did not perform imputation for them. As a result, the final analytical sample size was 1,906 instead of 1,930.

Missing values for the remaining participants were handled using multiple imputation with the mice package in R.

The results of the analysis based on the imputed dataset are presented in the table below. The model fitted on the imputed data demonstrated much better goodness-of-fit indices, including CMIN/DF, RMSEA, and SRMR, compared to the complete-case analysis. Based on these improvements, we decided to update the results of the manuscript accordingly. In addition, the other tables ("Table 2 Standardized path coefficients"), figure ("Figure 1 Flow chart demonstrating the process used to select the study participants." , "Figure 3 Path diagram and all statistically significant pathways"), supplementary materials ("Supplementary- Table 1 Descriptive characteristics", "Supplementary Table 4 Exponentiated Path coefficient analysis") and the discussion section were revised to reflect these changes.

Overall, the direction and magnitude of most associations remained largely unchanged, indicating that the findings were robust to the method used for handling missing data. However, there were a few notable differences. In the path analysis based on the imputed dataset, sex and Café-au-lait spots were no longer significantly associated with the T2D. In contrast, the associations of onychomycosis and rosacea with T2D, which were not statistically significant in the complete-case analysis, became statistically significant after imputation. Apart from these changes, the remaining results were consistent with those reported in the original analysis.

Furthermore, the Methods section was updated to include the use of multiple imputation with the mice package in R (Page 5 - lines 100-101)

Table 2 Standardized path coefficients

	Estimate (95% CI)	P-value	Estimate (95% CI) (Imputation)	P-value (Imputation)
**T2D**				
**SEX → T2D**	0.024 (0.001, 0.046)	0.038	0.004 (-0.018, 0.026)	0.746
**BMI → T2D**	0.042 (0.031, 0.054)	<0.001	0.011 (0.008, 0.014)	<0.001
**Sleep quality → T2D**	-0.005 (-0.016, 0.006)	0.398	-0.005 (-0.016, 0.005)	0.332
**Psoriasis → T2D**	0.109 (0.037, 0.181)	0.003	0.1 (0.056, 0.145)	<0.001
**Pityriasis versicolor → T2D**	0.109 (0.03, 0.189)	0.007	0.087 (0.037, 0.137)	0.001
**→ Tinea pedis T2D**	0.032 (0.006, 0.057)	0.014	0.065 (0.039, 0.092)	<0.001
**Onychomycosis → T2D**	0.033 (-0.005, 0.072)	0.090	0.041 (0.01, 0.071)	0.008**
**Café-au-lait spots → T2D**	-0.038 (-0.071, -0.004)	0.026	-0.043 (-0.101, 0.015)	0.142
**Lentigo senilis → T2D**	-0.025 (-0.057, 0.007)	0.126	-0.035 (-0.08, 0.011)	0.136
**Rosacea → T2D**	0.011 (-0.021, 0.042)	0.511	0.03 (0.004, 0.056)	0.023**
**BMI**				
**Diet → BMI**	0.171 (0.013, 0.329)	0.034	0.949 (0.386, 1.512)	0.001
**Anxiety → BMI**	-0.008 (-0.089, 0.074)	0.856	0.012 (-0.91, 0.934)	0.979
**Depression → BMI**	0.033 (-0.046, 0.113)	0.413	0.383 (-0.403, 1.168)	0.34
**Diet**				
**Education → Diet**	-0.039 (-0.057, -0.021)	<0.001	-0.097 (-0.135, -0.06)	<0.001
**Hyperhidrosis**				
**Diet → Hyperhidrosis**	0.003 (-0.021, 0.027)	0.812	-0.005 (-0.023, 0.014)	0.615
**Anxiety → Hyperhidrosis**	0.005 (-0.003, 0.013)	0.251	0.018 (-0.023, 0.059)	0.382
**Sleep quality**				
**Bmi → Sleep quality**	0.065 (0.011, 0.118)	0.017	0.017 (0.004, 0.03)	0.011
**Anxiety → Sleep quality**	0.077 (-0.005, 0.159)	0.064	0.285 (0.022, 0.549)	0.034
**Depression → Sleep quality**	0.095 (0.015, 0.175)	0.020	0.351 (0.113, 0.589)	0.004
**Tinea pedis**				
**Hyperhidrosis → Tinea pedis**	0.175 (0.018, 0.332)	0.029	0.262 (0.131, 0.392)	<0.001
**Chi-square goodness fit test**	324.38	<0.001	226.773	<0.001
**DF**	55		55	
**CMIN/DF**	5.89		4.12	
**RMSEA**	0.060		0.04	
**SRMR**	0.045		0.031	

Note that: T2D: Type 2 diabetes; BMI: body mass index; SE: Standard Error; RMSEA: root mean square error approximation; SRMR: standardized root mean square residual. Variables listed in the

above are standardized variables that are transformed version of an original variable that have been adjusted to have a mean of 0 and a standard deviation of 1

Reviewer: 1

If you have selected ‘Yes’ above, please provide details of any competing interests.: Not applicable
