# Supplementary material for: Assessing the association of type 2 diabetes with skin health status: a study of the Northern Finland Birth Cohort 1966
Source: BMJ Open. 2026 Jul 10;16(7):e109709. doi: 10.1136/bmjopen-2025-109709 (PMC13358341; doi:10.1136/bmjopen-2025-109709)
Supplement: Author's manuscript [file bmjopen-2025-109709.draft_revisions.pdf]

BMJ Open is committed to open peer review. As part of this commitment we make the peer review history of every article we publish publicly available.

When an article is published we post the peer reviewers' comments and the authors' responses online. We also post the versions of the paper that were used during peer review. These are the versions that the peer review comments apply to.

The versions of the paper that follow are the versions that were submitted during the peer review process. They are not the versions of record or the final published versions. They should not be cited or distributed as the published version of this manuscript.

BMJ Open is an open access journal and the full, final, typeset and author-corrected version of record of the manuscript is available on our site with no access controls, subscription charges or pay-per-view fees (http://bmjopen.bmj.com).

If you have any questions on BMJ Open's open peer review process please email info.bmjopen@bmj.com

BMJ Open

Assessing association of Type 2 diabetes with skin health status; study in the NFBC 1966

Journal:	*BMJ Open*
Manuscript ID	bmjopen-2025-109709
Article Type:	Original research
Date Submitted by the Author:	21-Aug-2025
Complete List of Authors:	Nasserinejad, Maryam; University of Oulu, Research Unit of Population Health, Faculty of Medicine<br>Huilaja, Laura; University of Oulu, Research Unit of Clinical Medicine, Medical Research Center<br>Sinikumpu, Suvi-Päivikki; University of Oulu, Research Unit of Clinical Medicine, Medical Research Center<br>Vainio, Seppo ; University of Oulu, Faculty of Biochemistry and Molecular Medicine, Disease Networks, Laboratory of Developmental Biology<br>Röning, Juha ; University of Oulu, Biomimetics and Intelligent Systems Group, Faculty of Information Technology and Electrical Engineering<br>Ali, Nsrein ; University of Oulu, Faculty of Biochemistry and Molecular Medicine, Disease Networks, Laboratory of Developmental Biology; University of Oulu, Research Unit of Health Sciences and Technology. Faculty of Medicine<br>Sebert, Sylvain; University of Oulu, Research Unit of Population Health, Faculty of Medicine
Keywords:	Dermatology < INTERNAL MEDICINE, Public health < INFECTIOUS DISEASES, General diabetes < DIABETES & ENDOCRINOLOGY

SCHOLARONE™
Manuscripts

I, the Submitting Author has the right to grant and does grant on behalf of all authors of the Work (as defined in the below author licence), an exclusive licence and/or a non-exclusive licence for contributions from authors who are: i) UK Crown employees; ii) where BMJ has agreed a CC-BY licence shall apply, and/or iii) in accordance with the terms applicable for US Federal Government officers or employees acting as part of their official duties; on a worldwide, perpetual, irrevocable, royalty-free basis to BMJ Publishing Group Ltd ("BMJ") its licensees and where the relevant Journal is co-owned by BMJ to the co-owners of the Journal, to publish the Work in this journal and any other BMJ products and to exploit all rights, as set out in our licence.

The Submitting Author accepts and understands that any supply made under these terms is made by BMJ to the Submitting Author unless you are acting as an employee on behalf of your employer or a postgraduate student of an affiliated institution which is paying any applicable article publishing charge ("APC") for Open Access articles. Where the Submitting Author wishes to make the Work available on an Open Access basis (and intends to pay the relevant APC), the terms of reuse of such Open Access shall be governed by a Creative Commons licence – details of these licences and which Creative Commons licence will apply to this Work are set out in our licence referred to above.

Other than as permitted in any relevant BMJ Author's Self Archiving Policies, I confirm this Work has not been accepted for publication elsewhere, is not being considered for publication elsewhere and does not duplicate material already published. I confirm all authors consent to publication of this Work and authorise the granting of this licence.

For peer review only

Assessing association of Type 2 diabetes with skin health status; study in the NFBC 1966

Maryam Nasserinejad [1]✉, Laura Huilaja [2], Suvi-Päivikki Sinikumpu [2], Seppo Vainio [3], Juha Röning [4], Nsrein Ali [3,5], Sylvain Sebert [1]

[1] Research Unit of Population Health, Faculty of Medicine, University of Oulu, Oulu, Finland. [2] Research Unit of Clinical Medicine, Medical Research Center, University of Oulu and Department of Dermatology, Oulu University Hospital, Oulu, Finland. [3] Faculty of Biochemistry and Molecular Medicine, Disease Networks, Laboratory of Developmental Biology, University of Oulu, Oulu, Finland. [4] Biomimetics and Intelligent Systems Group, Faculty of Information Technology and Electrical Engineering, University of Oulu, 90014 Oulu, Finland. [5] Research Unit of Health Sciences and Technology. Faculty of Medicine, 90220Oulu. Oulu Finland. ✉

email: maryam.nasserinejad@oulu.fi

Abstract

Background: Extensive research has identified comorbidities associated with type 2 diabetes (T2D); however, its relationship with skin disorders remains poorly understood. While evidence suggests skin disorders frequently co-occur with T2D, the underlying mechanisms remain unclear. Despite their clinical relevance, skin conditions are often underdiagnosed in diabetes care. This study applies variable selection methods to identify key dermatological factors linked to T2D and examines their direct and indirect associations using path analysis.

Methods: We analyzed data from the Northern Finland Birth Cohort 1966 (NFBC1966), including 1,930 participants (1,036 women, 894 men) at ages 31 and 46. T2D was defined based on oral glucose tolerance tests, self-reports, and Metformin prescriptions. Skin conditions were assessed through dermatologist diagnoses and questionnaires. Of 47 skin-related variables, key predictors were selected using test-based and penalty-based methods.

For peer review only - http://bmjopen.bmj.com/site/about/guidelines.xhtml

Results: Psoriasis (OR 1.115, 95% CI 1.038–1.198, p = 0.033) and Tinea pedis (OR 1.033, 95% CI 1.006–1.059, p = 0.014) showed direct associations with T2D. Hyperhidrosis (OR 1.175, 95% CI 1.018–1.394, p = 0.029) exhibited an indirect effect.

Conclusion: These results underscore the interconnectedness of dermatological conditions such as psoriasis and Tinea pedis with T2D, while highlighting the potential role of hyperhidrosis as an indirect factor in diabetic manifestations.

Keywords: type 2 diabetes, psoriasis, skin health status, association.

For peer review only

For peer review only - http://bmjopen.bmj.com/site/about/guidelines.xhtml

Introduction

Type 2 diabetes (T2D) is one main segments of chronic non-communicable, metabolic disease characterized by elevated levels of blood glucose which associated with serious complication to the heart, blood vessels, digestive system, eyes, kidneys, nerves, skin and even psychosocial aspect and early mortality [1].

An estimated 537 million adults aged 20–79 years worldwide (10.5% of all adults in this age group) have diabetes. By 2030, this number is projected to rise to 643 million, and by 2045, it could reach 783 million. This represents a 46% increase in diabetes cases, while the global population is estimated to grow by only 20% over the same period [2]. In the WHO European Region alone, approximately 64 million adults and around 300,000 children and adolescents are living with diabetes as of 2024, making it one of the most common chronic conditions in Europe [3]. According to the latest reports, among the European Nordic countries, Finland has the highest prevalence of diabetes with 9.7% and the number of adults with diabetes in 1000s is 392.9 (95% CI: 334.8,435.9) [2].

On the other hand, the burden and premature mortality of diabetes are very high and most people with diabetes struggle with other complication which leads to a drastic reduction in their quality of life and compared to individuals without diabetes, diabetes patients have lower health-related quality of life [4].

The comorbidity of skin disease and diabetes is undeniable, and almost one third of people with diabetes have skin lesions [5]. In general population there is a possibility that mild skin diseases such as eczema, psoriasis, skin spots, bacterial infection, itchy skin etc. by themselves receive less attention and treatment, and when they are associated with diabetes, they become important, and people seek treatment for these. In the causes of skin diseases, Inflammation is a well-established factor in the development of skin diseases, including psoriasis, atopic dermatitis, and seborrheic dermatitis [6]. Accordingly, we will devote a part of this study to investigating inflammatory diseases and diabetes.

For peer review only

Numerous studies have shown the association between skin disorders and diabetes. for example, substantial evidence from various studies supports a significant link between inflammatory diseases, such as psoriasis and eczema, and T2D [8][9]. Additionally, Azfar et al. identified psoriasis as a risk factor for the development of T2D [10].

Conditions commonly associated with diabetes—such as acanthosis nigricans, diabetic dermopathy, necrobiosis lipoidica, bacterial and fungal infections, and xerosis (dry skin)—often appear early, sometimes even before diabetes is diagnosed. These skin disorders not only reflect the systemic effects of diabetes but can also serve as early indicators, emphasizing the importance of timely recognition and management for improved outcomes [11][12]. Beyond inflammation, fungi and bacteria may also contribute to the co-occurrence of skin diseases and diabetes, emphasizing the need for further investigation into these potential connections.

This study explores the relationship between skin health status and T2D in northern Finland. To achieve this, we begin by identifying the most relevant skin health status associated with T2D. Variable selection is a crucial step in inferential modeling, as accurately identifying the subset of variables truly associated with the outcome of interest is essential. General approaches to variable selection fall into three main categories: test-based, penalty-based, and screening-based methods [13]. While various techniques for variable selection exist, each has distinct mathematical properties, which can lead to differing outcomes in terms of the variables selected. This raises an important challenge for researchers: understanding how the choice of selection method affects study results and their reproducibility across different methods. Investigating the "between-method" variability in variable selection is, therefore, an important area of study.

Best practices suggest employing multiple methods and assigning confidence to variables based on their consistency across methods. In this study, we apply several variable selection techniques to dermatological data to identify the most appropriate variables for path analysis modeling.

In the path model, we examined the direct relationships between selected skin health status and T2D, adjusting for potential confounders and covariates such as diet, education, BMI, anxiety, depression, and sex.

For peer review only - http://bmjopen.bmj.com/site/about/guidelines.xhtml

Materials and methods

Overview and data resources

The selected population

In this study, we used the data from the participants of the Northern Finland Birth Cohort (NFBC) 1966 [14].

The NFBC1966 is a longitudinal population-based study initiated to investigate factors affecting health and development from prenatal stages into adulthood. The cohort comprises 12138 live births from 1966, representing 96.3% of all births that year in Finland's two northernmost provinces, Oulu and Lapland. Data collection began during pregnancy, starting between the 10th and 16th gestational weeks, and has continued through multiple follow-up stages, including assessments at ages 1, 14, 31, and 46. The comprehensive data encompass health records, lifestyle factors, and demographic information, facilitating extensive research into various health outcomes, such as neurological development, chronic diseases, and the impact of perinatal events on long-term health [15].

The current study aimed at including all participants with available data on skin health status and type 2 diabetes based on their participation to follow-ups organized when the participants were approximately 46 years (in 2012), while also utilizing covariate data from age 31 (collected in 1997). Following logistic and financial constraints, the clinical examination for dermatology diagnosis was only intended to participants living in the region of Oulu and was conducted at the University Hospital of Oulu. It included a total of 894 males and 1036 females (N = 1930). The studies received ethical approval from Northern Ostrobothnia Hospital District Ethical Committee 94/2011 (12.12.2011).

Exposure: Skin health status variables

For peer review only - http://bmjopen.bmj.com/site/about/guidelines.xhtml

Each individual Skin Health Status could be defined from 46 (Supplementary Table 1) different variables. These variables or measured were part of a dermatological assessment designed by dermatologist to study the prevalence of major skin findings and diseases and disorders in mid-life [16].

The examination included 46 skin health status variables, with clinical evaluations conducted by a dermatologist at age 46 through questionnaire at the age of 46y.In addition, the information for psoriasis was self-reported through postal questionnaires with 7150 (3849 women, 3301 men) individuals.

Fig. 1 shows a flow chart outlining the process used to select participants for this study.

Outcome: T2D

To ascertain individuals with T2D, our approach was multifaceted. Firstly, we identified individuals based on specific criteria related to their oral glucose tolerance test (OGTT) results: if the fasting OGTT value was 7 mmol/l or higher, and the value at two hours after 75g anhydrous glucose was 11.1 mmol/l or greater. Additionally, we incorporated self-reported data obtained through questionnaire responses. Participants were asked whether they currently have or have ever had adult-onset diabetes (type 2), the affirmative responses considered as T2D. Furthermore, the use of blood sugar-lowering medications like metformin was recognized as a valid marker for T2D diagnosis. This comprehensive methodology ensured a robust identification process for individuals with T2D, incorporating both clinical measurements and self-reported information.

Covariates

To account for the long-term impact of diet, education, anxiety, and depression, we employed data from 31 ages in this study.

For defining anxiety and depression, we followed Hopkins symptom checklist [17]. This questionnaire consists of 14 questions aimed at diagnosing anxiety, focusing on symptoms like

For peer review only

For peer review only - http://bmjopen.bmj.com/site/about/guidelines.xhtml

pain, tension, restlessness, trembling, fear and heart pounding, and 13 questions designed to assess depression, covering issues such as difficulty falling asleep, feelings of loneliness and worthlessness, sexual difficulties, lack of energy, and suicidal thoughts. The questions were presented in a four-point Likert format, with one being 'not at all' and four being 'very much.' The depression and anxiety scores were derived from the mean scores, which ranged slightly from 1 to 4, where 1 indicated no anxiety or depression and 4 indicated a high level of anxiety or depression.

Diet was categorized as a binary variable (healthy vs. unhealthy) based on consumption patterns of vegetables, fruits, rye, and processed foods [18]. Education was defined by a combination of two different questions of basic education and vocational qualifications with three levels from basic educated to highly educated.

Information on poor quality sleep was collected by questionnaire at age 46 years.

Statistical methods

Descriptive statistics, including counts and percentages for all variables, were computed overall and stratified by sex (male and female) and health status (healthy and T2D groups). Chi-square tests were used to compare categorical variables between sex groups and between health status groups as presented. Initially, we applied the exploratory factor analysis (EFA) to reduce the dimensions of the skin status variables, aiming to derive components from the pool of 47skin health status. However, due to the Kaiser–Meyer–Olkin (KMO) index of 0.62 and a low cumulative variance (0.275 out of 1.00) for just the first 22 components, we opted against utilizing the outcomes of this model. An alternative approach to reducing dimensionality is variable selection. In this study, we employed a variety of methods for this purpose, including Single covariate techniques based on p-values, as well as backward stepwise logistic regression (BSLR), forward stepwise logistic regression (FSLR), and stepwise logistic regression (SLR). Additionally, we utilized machine learning techniques, specifically least absolute shrinkage and selection operator regression (Lasso), Ridge regression, minimax convex penalty (MCP), and Sparse Stepwise

For peer review only - http://bmjopen.bmj.com/site/about/guidelines.xhtml

For peer review only

Regression. The degree of agreement between the results of various methods was assessed using Cohen's Kappa index, which ranges from -1 to 1. Values less than 0 indicate poor agreement (worse than chance), A Kappa value below 0 indicates poor agreement (worse than chance), while values between 0.61 and 0.80 suggest substantial agreement, and values above 0.80 indicate almost perfect agreement [19].

Following the selection of skin health status, we employed path analysis to examine both direct and indirect associations, while controlling for the effects of sex, body mass index (BMI), diet, education, depression, and anxiety (Supplementary Fig. 1). Notably, we utilized the glmnet package for Lasso and Ridge regression analysis, ncvreg package for MCO method, sparsestep package [20] for sparse step method and the lavaan package for path analysis. All analyses were conducted using R software, version 4.4.0.

Results

Among the study participants, the overall prevalence of T2D was 4.8%, with a significantly higher rate observed in men (5.6%) compared to women (4.1%). The prevalence of tinea pedis was 27%, with a higher incidence in men than in women. Specifically, among patients with T2D, the prevalence of tinea pedis increased to 44.4%, with 55.5% in men and 35.1% in women.

Psoriasis was present in 2.3% of the participants overall, with a higher prevalence in patients with T2D at 4.7%. Contradictory to tinea pedis, notably, this prevalence was twice as high in women (6%) compared to men (3.5%). Rosacea affected 15.2% of the participants, and among those with T2D, the prevalence rose to 23.2%, showing a significant gender disparity with 8.88% in men and 35.18% in women.

Onychomycosis was observed in 9.4% of the participants. Café-au-lait spots were found in 12.5% of the overall population, but only in 3.0% of those with T2D, with a notable gender difference: 5.5% in women and no cases reported in men with T2D. Hyperhidrosis was reported in 2.2% of the participants. Lentigosenilis had a prevalence of 13.4%, while Pityriasis versicolor was observed in 1.9% of the participants. Among those with T2D, the prevalence of Pityriasis

For peer review only

For peer review only - http://bmjopen.bmj.com/site/about/guidelines.xhtml

versicolor was 5.1%, with a higher prevalence in men (6.0%) compared to women (3.7%) (Table 1).

Variable selection

A total of 17 variables were selected using the ridge method, 15 variables with the lasso method, 12 through single covariate analysis, 18 with BSLM, and 15 with sparse step. The FSLM and SLM methods did not select any variables, and 18 variables were not selected by any of the methods (Fig. 2). Significant variables were identified based on predefined criteria for each method: ridge and lasso regression selected variables with nonzero coefficients, single covariate analysis considered variables significant at $p < 0.05$, and BSLM and sparse step methods applied model-specific thresholds optimizing predictive performance.

Overall, excluding the two methods FALM and SLM, the combined agreement among the remaining methods was 0.507—a strong value for six different methods, indicating reliable results. In addition, pairwise agreement analysis showed that MCP and BSLM, as well as BSLM and Lasso, had the highest agreement values, at 0.96 and 0.86, respectively. The lowest agreement was observed between single covariate Analysis and Ridge (Supplementary Table 2). A total of 15 variables were initially selected using various methods. From these, to ensure proper control over Type I and Type II errors, we filtered results with significant p-values in the single covariate analysis. Machine learning models optimize for prediction accuracy, they do not inherently provide statistical significance measures. By applying a p-value threshold, we identified statistically robust associations and reduced the risk of false positives. This approach allowed us to combine the predictive power of machine learning with the inferential strength of traditional statistical methods, ensuring more reliable and interpretable findings. These variables are: Tinea pedis, Psoriasis, Rosacea, Onychomycosis, café-au-lait spots, excessive sweating, Lentigosenilis, and pityriasis versicolor. It is worth noting that the multicollinearity of the selected variables was assessed by the variance inflation factor (VIF) (Supplementary Table3).

For peer review only - http://bmjopen.bmj.com/site/about/guidelines.xhtml

For peer review only

Path analysis

In this study, to facilitate comparison and eliminate the influence of variation due to different scales among the variables, standardization was conducted. Standardization transforms original variables to have a mean of 0 and a standard deviation of 1, making comparison between variables with different scales or units more straightforward.

The results of the path analysis with the standardized path coefficients are depicted in Fig. 3 and Table 2 and the exponentiate of coefficients in supplementary Table 4. Model fit was evaluated using several indices. The RMSEA (0.060) and SRMR (0.045) were both below 0.08 and the relative Chi-square (CMIN/DF) was calculated as 5.89, which is <6. These findings indicate an acceptable fit of the model to the data.

In the model, sex had a direct effect on T2D (OR 1.024, 95%CI 1.001- 1.047), with males having a higher risk compared to females, which was statistically significant at the 0.05 level. BMI had a direct effect on T2D (OR 1.043, 95%CI 1.031- 1.055) and an indirect effect, mediated through Poor sleep quality (OR 1, 95 %CI 1- 1.001). The total effect was the sum of direct and indirect effects, at 1.043 with a 95% CI of 1.031-1.056. The total effect was significant at the 0.05 level. Poor quality of sleep did not show any direct effect on T2D (OR 0.995, 95%CI 0.984-1.006). Diet (OR 1, 95%CI 1- 1.001), Anxiety (OR 0.999, 95%CI 0.998- 1.005), Depression (OR 1, 95%CI 1- 1.001) and Education (OR 1, 95%CI 1- 1) did not show any indirect effect on T2D.

The pathway shows that tinea pedis (OR 1.033 95%CI 1.006-1.059), psoriasis (OR 1.115 95%CI 1.038-1.198), and Pityriasis versicolor (OR 1.115 95%CI 1.03-1.208) had positive direct effects and Café-au-lait spots (OR 0.975 95%CI 0.931-0.996) had a negative direct effect on T2D.

Hyperhidrosis had an indirect effect on T2D, mediated through tinea pedis (OR 1.006, 95%CI 1-1.0019) (Supplementary Table 4).

Discussion

For peer review only - http://bmjopen.bmj.com/site/about/guidelines.xhtml

To the best of our knowledge, this is the first study to simultaneously investigate multiple skin diseases in relation to diabetes. The innovative use of a combination of machine learning techniques and traditional statistical methods for variable selection has led to more robust and high-quality results, with a promising agreement rate of 0.507 across six different methods. This study identified a significant direct association between T2D and pityriasis versicolor, psoriasis, and tinea pedis, with odds ratios of 1.115 (95% CI: 1.03, 1.208), 1.115 (95% CI: 1.038, 1.198), and 1.033 (95% CI: 1.006, 1.059), respectively. In contrast, an inverse association was found between café-au-lait spots and T2D, with an odds ratio of 0.963 (95% CI: 0.931, 0.996).

In our study, we found that the prevalence of psoriasis from self-reported information in the population of 46 years was 2.3% and we did not find any difference between men and women. We identified a significant association between T2D and psoriasis, with individuals who have psoriasis being 1.115 times more likely to develop T2D compared to those without psoriasis. This translates to a 0.115 increase in the odds of having T2D for individuals with psoriasis. These findings align with prior research, such as the meta-analysis conducted by Mirghani H et al., which reported an OR of 1.38 (95% CI: 1.17-1.64) for the association between psoriasis and diabetes. It is worth noting that while the meta-analysis included both type 1 and type 2 diabetes, our study specifically focused on T2D [21].

It is suggested that diabetes and psoriasis may exacerbate each other, potentially creating a vicious cycle where the presence of one condition aggravates the severity of the other [22][23]. The link between psoriasis and diabetes was proposed in other reports demonstrating that psoriasis may increase the risk of developing diabetes. People with psoriasis are more likely to develop diabetes compared to those without the condition, and the risk appears to be even higher in severe cases [24][25][26]. Additionally, psoriasis may contribute to insulin resistance, a key factor in the onset of T2D [27]. On the other hand, diabetes, especially when poorly managed, might also increase the likelihood of developing psoriasis. This complex, possibly bidirectional relationship suggests that chronic inflammation and immune system dysfunction are central to the development of both diseases. This may be explained by the shared genes and molecular

For peer review only - http://bmjopen.bmj.com/site/about/guidelines.xhtml

For peer review only

pathways recently identified to be linked with T2D and psoriasis pathogenesis [28]. This bidirectional relationship highlights the importance of careful management and monitoring of both conditions to prevent worsening outcomes.

In addition, we examined three fungal diseases: pityriasis versicolor, tinea pedis, and onychomycosis. We observed a direct relationship between diabetes and all three conditions; however, the relationship between onychomycosis and diabetes was not statistically significant in our path model. Our findings regarding fungal infections are consistent with previous research [21][22]. Saud B et al., reported that the prevalence of fungal diseases in diabetic patients is 7.2 times higher compared to individuals without diabetes [29]. Furthermore, participants exhibiting abnormal skin findings in the toe web areas had a 2.5 times increased risk of having undiagnosed diabetes based on OGTT results. The risk was even higher (6 times) when assessed using HbA1c levels [30]. This highlights the potential role of fungal disease, particularly in the toe web regions, as an indicator for identifying individuals at higher risk for diabetes, which may facilitate earlier detection and intervention.

In our study, individuals with tinea pedis and pityriasis versicolor were found to be 1.03 and 1.115 times more likely, respectively, to present with T2D compared to those without these fungal infections. These results highlight the importance of monitoring and managing fungal infections in patients with diabetes to potentially mitigate the risk and complications associated with T2D.

Furthermore, this study is the first to investigate the relationship between café-au-lait spots and lentigo senilis with T2D. Our findings suggest that individuals with café-au-lait spots and lentigo senilis may have a lower prevalence of T2D compared to those without these skin conditions, though more studies are needed to prove this relationship. Regarding rosacea, we identified a direct association with T2D, although this association was not statistically significant. Interestingly, a study by Stefani EC et al. identified T2D as a risk factor for rosacea [12]. This hypothesis has been addressed by spoendlin and colleagues, who conducted a population-based case–control analysis using UK-based General Practice Research Database to analyze the association between rosacea and T2D [32].

For peer review only

For peer review only - http://bmjopen.bmj.com/site/about/guidelines.xhtml

Moreover, our study identified hyperhidrosis as an indirect risk factor for T2D. This finding aligns with the literature, which indicates that individuals with T2D exhibit impaired thermoregulation compared to healthy individuals, resulting in sweating dysfunction. This impairment may be attributed to several factors, including cardiovascular complications, metabolic imbalances such as irregular insulin distribution and chronic hyperglycemia, alterations in autonomic nervous function, and reduced blood flow to the skin. Collectively, these factors may influence the sweat gland's function leading to a diminished ability to sweat [33][34][35][36][37].

Conclusion

In this study, our aim was to investigate the pathway effects of skin diseases on T2D. We employed advanced statistical techniques for variable selection and innovatively combined machine learning approaches with traditional methods, ensuring more robust and reliable findings. the results of path analysis revealed that tinea pedis, psoriasis, and pityriasis versicolor had a positive effect on T2D, while café-au-lait spots were associated with a negative effect on the condition.

These results have important implications for people's health and quality of life. first, Skin diseases are often considered insignificant and, particularly when accompanied by other illnesses, their treatment is prioritized secondarily. However, people's skin health status is closely linked to their overall health and neglecting it may lead to more serious complications. Second, in people with T2D, skin problems are frequently overlooked until they become severe. Our study has shown that skin diseases are associated with T2D. Based on these findings, we strongly recommend that healthcare providers give more attention to skin health in people with T2D, ensuring timely diagnosis and treatment to prevent complications and improve people's overall well-being.

However, these studies have faced limitations. In the NFBC1966 study, data on skin conditions were collected by dermatologists at the age of 46 for individuals who were readily accessible, primarily residing in northern Finland. While the involvement of dermatologists enhances the

For peer review only - http://bmjopen.bmj.com/site/about/guidelines.xhtml

validity of the data, this regional focus, along with the limited sample size, restricts the generalizability of the findings to the broader Finnish population. Furthermore, the data were collected at a single time point, so it was impossible to longitudinal assessment of skin diseases. The dataset also contained a high volume of missing values for certain skin-related variables, which limited the feasibility of applying more complex models. Consequently, we had to rely on simpler models and relationships to maintain the validity of our findings. Moreover, this was a cross-sectional study where external factors were not controlled, making it impossible to establish causal relationships. Therefore, it is recommended to use clinical trials study to investigate causal relationships or employ genetic methods such as Mendelian Randomization (MR) to explore the causal link between skin diseases and diabetes.

Finally, it is essential to note that skin problems are common in diabetic patients and can be indicative of poor blood sugar control. Early diagnosis and appropriate treatment of these issues can significantly improve patients' quality of life.

Acknowledgments

We designed this study, while Laura and Suvi contributed by providing expertise on skin diseases and offering comments on the research. All authors have read and approved the final version of the study.

We wish to thank all cohort members, researchers and NFBC project center personnel who participated in the NFBC data collections.

Data Availability Statement

NFBC data are available from the University of Oulu, Infrastructure for Population Studies. Permission to use the data can be applied for research purposes via an electronic material request portal. In the use of data, we follow the EU general data protection regulation (679/2016) and the Finnish Data Protection Act.

The use of personal data is based on a cohort participant's written informed consent in their latest follow-up study, which may cause limitations to its use. Please, contact the NFBC project center

For peer review only

(NFBCprojectcenter(at)oulu.fi) and visit the cohort website (www.oulu.fi/nfbc) for more information.

Competing interests

No, there are no competing interests for any author.

References

[1] N. D. D. G. (US), N. I. of Diabetes, and K. D. (US), *Diabetes in America*, no. 95. National Institutes of Health, National Institute of Diabetes and Digestive …, 1995.

[2] H. Sun *et al.*, "IDF Diabetes Atlas: Global, regional and country-level diabetes prevalence estimates for 2021 and projections for 2045," *Diabetes Res. Clin. Pract.*, vol. 183, p. 109119, 2022.

[3] S. Del Prato, B. Torbeyns, and C. Mathieu, "2024: The year to take European action on diabetes to the next level," *Diabetologia*, vol. 67, no. 7, pp. 1155–1158, 2024, doi: 10.1007/s00125-024-06166-9.

[4] J. Holmes, S. McGill, P. Kind, J. Bottomley, S. Gillam, and M. Murphy, "Health-related quality of life in type 2 diabetes (TARDIS-2).," *Value Heal. J. Int. Soc. Pharmacoeconomics Outcomes Res.*, vol. 3 Suppl 1, pp. 47–51, 2000, doi: 10.1046/j.1524-4733.2000.36028.x.

[5] P. Gkogkolou and M. Böhm, "Skin disorders in diabetes mellitus," *JDDG - J. Ger. Soc. Dermatology*, vol. 12, no. 10, pp. 847–864, 2014, doi: 10.1111/ddg.12424.

[6] J. D. Bos, *Skin immune system (SIS): Cutaneous immunology and clinical immunodermatology*. CRC press, 1997.

[7] H. N. Wilkinson and M. J. Hardman, "Wound healing: cellular mechanisms and pathological outcomes.," *Open Biol.*, vol. 10, no. 9, p. 200223, Sep. 2020, doi: 10.1098/rsob.200223.

[8] A. S. Lonberg, L. Skov, A. Skytthe, K. O. Kyvik, O. B. Pedersen, and S. F. Thomsen, "Association of psoriasis with the risk for type 2 diabetes mellitus and obesity," *JAMA Dermatology*, vol. 152, no. 7, pp. 761–767, 2016, doi: 10.1001/jamadermatol.2015.6262.

For peer review only

[9] J. I. Silverberg and P. Greenland, “Eczema and cardiovascular risk factors in 2 US adult population studies,” *J. Allergy Clin. Immunol.*, vol. 135, no. 3, pp. 721-728.e6, 2015, doi: 10.1016/j.jaci.2014.11.023.

[10] R. S. Azfar, N. M. Seminara, D. B. Shin, A. B. Troxel, D. J. Margolis, and J. M. Gelfand, “Increased risk of diabetes mellitus and likelihood of receiving diabetes mellitus treatment in patients with psoriasis.,” *Arch. Dermatol.*, vol. 148, no. 9, pp. 995–1000, Sep. 2012, doi: 10.1001/archdermatol.2012.1401.

[11] M. I. Perez and S. R. Kohn, “Cutaneous manifestations of diabetes mellitus.,” *J. Am. Acad. Dermatol.*, vol. 30, no. 4, pp. 514–519, Apr. 1994, doi: 10.1016/s0190-9622(94)70058-3.

[12] E. C. Stefanadi *et al.*, “Metabolic syndrome and the skin: a more than superficial association. Reviewing the association between skin diseases and metabolic syndrome and a clinical decision algorithm for high risk patients.,” *Diabetol. Metab. Syndr.*, vol. 10, p. 9, 2018, doi: 10.1186/s13098-018-0311-z.

[13] T. Hastie, “The elements of statistical learning: data mining, inference, and prediction.” Springer, 2009.

[14] University of Oulu, “Northern Finland Birth Cohort 1966,” 1966. http://urn.fi/urn:nbn:fi:att:bc1e5408-980e-4a62-b899-43bec3755243

[15] T. Nordström *et al.*, “Cohort Profile: 46 years of follow-up of the Northern Finland Birth Cohort 1966 (NFBC1966).,” *Int. J. Epidemiol.*, vol. 50, no. 6, pp. 1786-1787j, Jan. 2022, doi: 10.1093/ije/dyab109.

[16] S.-P. Sinikumpu *et al.*, “High prevalence of skin diseases and need for treatment in a middle-aged population. A Northern Finland Birth Cohort 1966 study.,” *PLoS One*, vol. 9, no. 6, p. e99533, 2014, doi: 10.1371/journal.pone.0099533.

[17] D. Lhewa, S. Banu, B. Rosenfeld, and A. Keller, “Validation of a Tibetan translation of the Hopkins Symptom Checklist 25 and the Harvard Trauma Questionnaire.,” *Assessment*, vol. 14, no. 3, pp. 223–230, Sep. 2007, doi: 10.1177/1073191106298876.

[18] J. Laitinen, K. Pietiläinen, M. Wadsworth, U. Sovio, and M.-R. Järvelin, “Predictors of

For peer review only - http://bmjopen.bmj.com/site/about/guidelines.xhtml

abdominal obesity among 31-y-old men and women born in Northern Finland in 1966.," *Eur. J. Clin. Nutr.*, vol. 58, no. 1, pp. 180–190, Jan. 2004, doi: 10.1038/sj.ejcn.1601765.

[19] H. L. Kundel and M. Polansky, "Measurement of observer agreement.," *Radiology*, vol. 228, no. 2, pp. 303–308, Aug. 2003, doi: 10.1148/radiol.2282011860.

[20] G. J. J. van den Burg, P. J. F. Groenen, and A. Alfons, "SparseStep: Approximating the Counting Norm for Sparse Regularization," pp. 1–15, 2017, [Online]. Available: http://arxiv.org/abs/1701.06967

[21] H. Mirghani *et al.*, "The Association of Psoriasis, Diabetes Mellitus, and Hypertension: A Meta-Analysis.," *Cureus*, vol. 15, no. 11, p. e48855, Nov. 2023, doi: 10.7759/cureus.48855.

[22] R. Abramczyk, J. N. Queller, A. W. Rachfal, and S. S. Schwartz, "Diabetes and Psoriasis: Different Sides of the Same Prism.," *Diabetes. Metab. Syndr. Obes.*, vol. 13, pp. 3571–3577, 2020, doi: 10.2147/DMSO.S273147.

[23] A. Vesikansa *et al.*, "Comorbidities and Medication Use in Finnish Patients with Psoriasis: A Population-Based Registry Study.," *Acta Derm. Venereol.*, vol. 103, p. adv00886, Mar. 2023, doi: 10.2340/actadv.v103.3491.

[24] A. W. Armstrong, C. T. Harskamp, and E. J. Armstrong, "Psoriasis and the risk of diabetes mellitus: a systematic review and meta-analysis.," *JAMA dermatology*, vol. 149, no. 1, pp. 84–91, Jan. 2013, doi: 10.1001/2013.jamadermatol.406.

[25] M. T. Wan, D. B. Shin, R. A. Hubbard, M. H. Noe, N. N. Mehta, and J. M. Gelfand, "Psoriasis and the risk of diabetes: A prospective population-based cohort study.," *J. Am. Acad. Dermatol.*, vol. 78, no. 2, pp. 315-322.e1, Feb. 2018, doi: 10.1016/j.jaad.2017.10.050.

[26] M. Mamizadeh, Z. Tardeh, and M. Azami, "The association between psoriasis and diabetes mellitus: A systematic review and meta-analysis.," *Diabetes Metab. Syndr.*, vol. 13, no. 2, pp. 1405–1412, 2019, doi: 10.1016/j.dsx.2019.01.009.

[27] A. L. Neimann, D. B. Shin, X. Wang, D. J. Margolis, A. B. Troxel, and J. M. Gelfand, "Prevalence of cardiovascular risk factors in patients with psoriasis.," *J. Am. Acad.*

For peer review only - http://bmjopen.bmj.com/site/about/guidelines.xhtml

Dermatol., vol. 55, no. 5, pp. 829–835, Nov. 2006, doi: 10.1016/j.jaad.2006.08.040.

[28] L. Yang, L. Zhang, Q. Du, X. Gong, and J. Tian, "Exploring the molecular mechanism underlying the psoriasis and T2D by using microarray data analysis.," *Sci. Rep.*, vol. 13, no. 1, p. 19313, Nov. 2023, doi: 10.1038/s41598-023-46795-5.

[29] B. Saud *et al.*, "Fungal Infection among Diabetic and Nondiabetic Individuals in Nepal.," *Interdiscip. Perspect. Infect. Dis.*, vol. 2020, p. 7949868, 2020, doi: 10.1155/2020/7949868.

[30] S.-P. Sinikumpu *et al.*, "Abnormal skin in toe webs is a marker for abnormal glucose metabolism. A cross-sectional survey among 1,849 adults in Finland.," *Sci. Rep.*, vol. 7, no. 1, p. 9125, Aug. 2017, doi: 10.1038/s41598-017-09354-3.

[31] A. Akin Belli, S. Ozbas Gok, G. Akbaba, F. Etgu, and G. Dogan, "The relationship between rosacea and insulin resistance and metabolic syndrome.," *Eur. J. Dermatol.*, vol. 26, no. 3, pp. 260–264, Jun. 2016, doi: 10.1684/ejd.2016.2748.

[32] J. Spoendlin, J. J. Voegel, S. S. Jick, and C. R. Meier, "Risk of rosacea in patients with diabetes using insulin or oral antidiabetic drugs.," *J. Invest. Dermatol.*, vol. 133, no. 12, pp. 2790–2793, Dec. 2013, doi: 10.1038/jid.2013.225.

[33] S. Chahal, K. Vohra, and A. Syngle, "Association of sudomotor function with peripheral artery disease in type 2 diabetes.," *Neurol. Sci. Off. J. Ital. Neurol. Soc. Ital. Soc. Clin. Neurophysiol.*, vol. 38, no. 1, pp. 151–156, Jan. 2017, doi: 10.1007/s10072-016-2742-3.

[34] W. B. Horton and E. J. Barrett, "Microvascular Dysfunction in Diabetes Mellitus and Cardiometabolic Disease.," *Endocr. Rev.*, vol. 42, no. 1, pp. 29–55, Jan. 2021, doi: 10.1210/endrev/bnaa025.

[35] Y. Liu *et al.*, "Factors influencing sweat gland innervation in diabetes.," *Neurology*, vol. 84, no. 16, pp. 1652–1659, Apr. 2015, doi: 10.1212/WNL.0000000000001488.

[36] G. P. Kenny, R. J. Sigal, and R. McGinn, "Body temperature regulation in diabetes.," *Temp. (Austin, Tex.)*, vol. 3, no. 1, pp. 119–145, 2016, doi: 10.1080/23328940.2015.1131506.

[37] B. Liu, X. Cheng, M. Chen, W. Dong, J. Sun, and Y. Lun, "Changes of sweat gland

For peer review only

For peer review only - http://bmjopen.bmj.com/site/about/guidelines.xhtml

function in type 2 diabetes mellitus patients with peripheral neuropathy," *Int. J. Diabetes Dev. Ctries.*, vol. 44, no. 1, pp. 84–90, 2024.

For peer review only

For peer review only - http://bmjopen.bmj.com/site/about/guidelines.xhtml

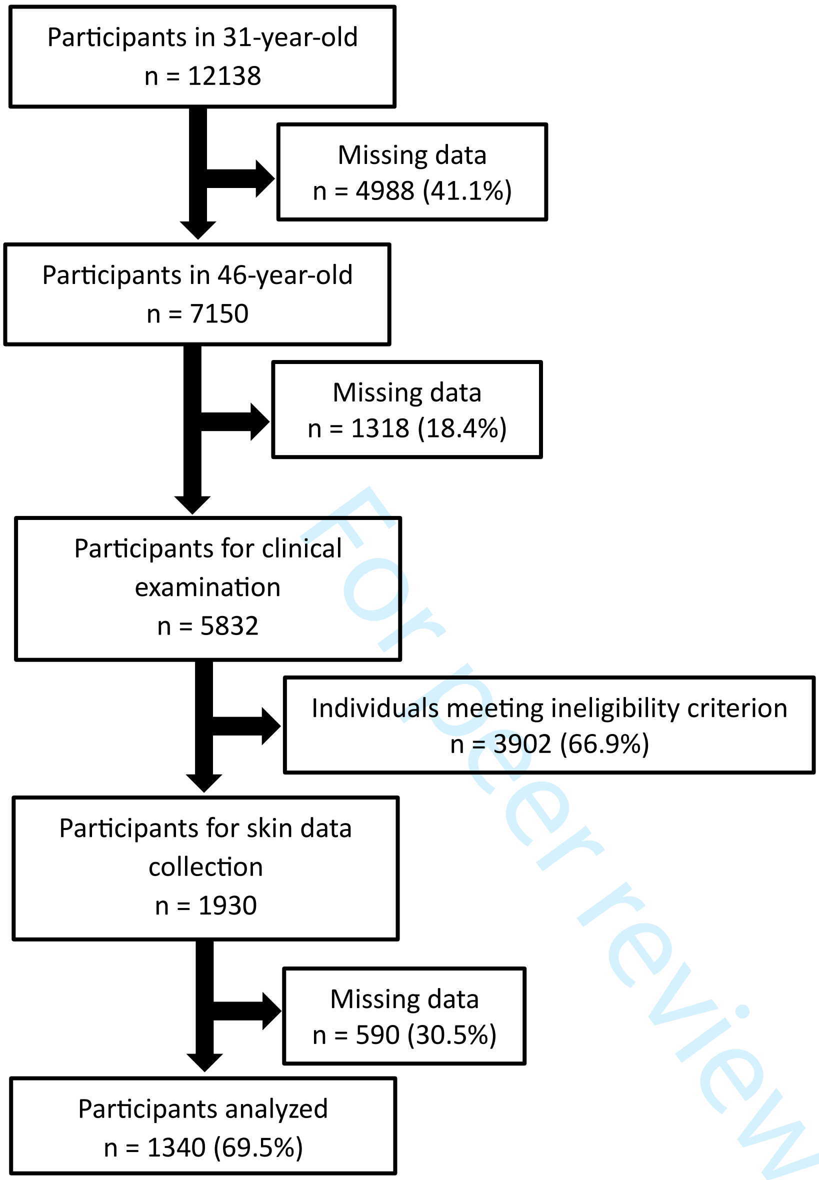

Figure 1 Flow chart demonstrating the process used to select the study participants.

For peer review only

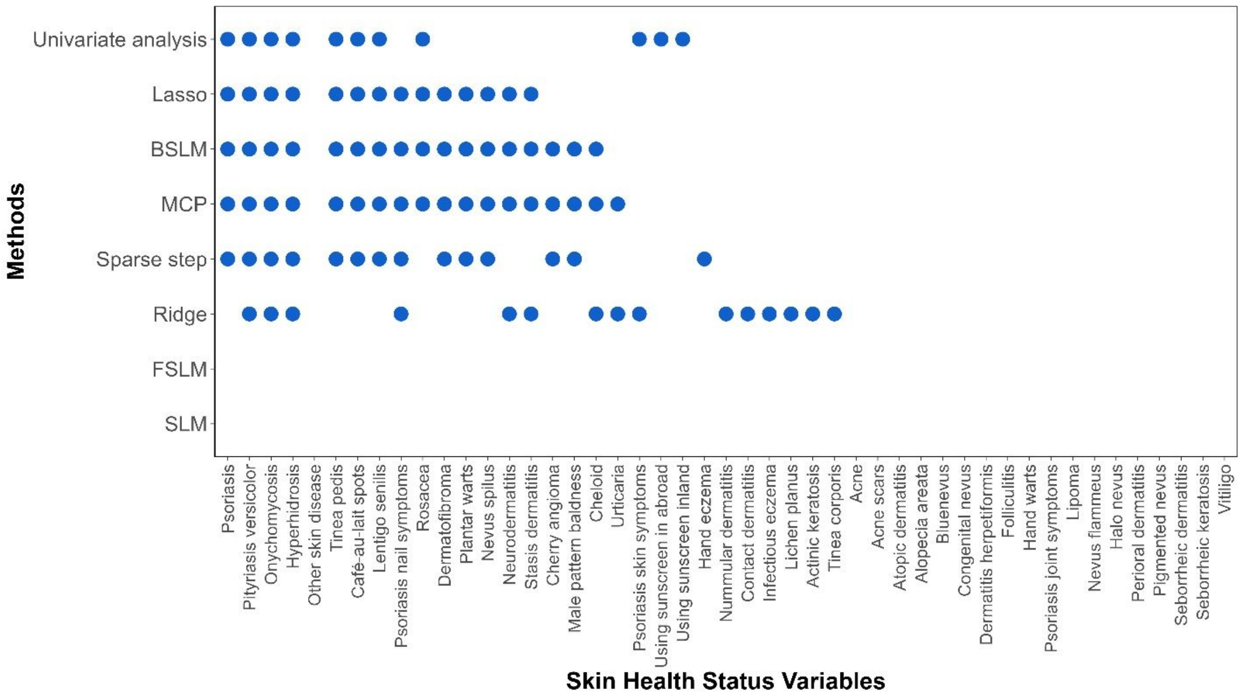

Figure 2 Results of variable selection methods

For peer review only

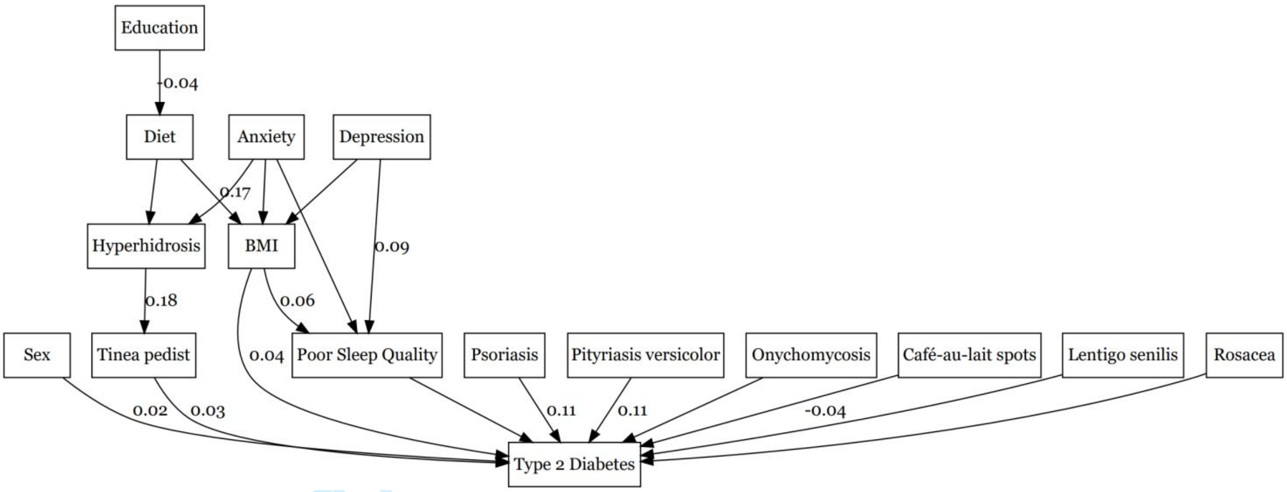

Figure 3 The path analysis was performed in 1930 individuals aged 46 years. All depicted arrows represent statistically significant associations (Beta coefficients). Model fit values: SRMR = 0.045, RMSEA = 0.060.

For peer review only

Table 1 Descriptive characteristics of the study population

Variables	Category	Overall	Men	Women	P-value[a]	Type 2 diabetes	healthy	P-value[b]
Sex	Male	3027 (45.5 %)	-	-	-	180 (53.6 %)	3027 (45.5 %)	0.0042
	Female	3633 (54.5 %)	-	-		156 (46.4 %)	3633 (54.5 %)	
BMI (kg/m2)	Underweight	37 (0.6 %)	7 (0.3 %)	30 (0.9 %)	P<0.001	0 (0.0 %)	37 (0.7 %)	P<0.001
	Normalweight	2188 (37.6 %)	735 (28.7 %)	1453 (44.6 %)		25 (8.8 %)	2137 (39.3 %)	
	Overweight	3594 (61.8 %)	1819 (71.0 %)	1775 (54.5 %)		259 (91.2 %)	3262 (60.0 %)	
Diet	Unhealthy	5524 (88.8 %)	2317 (83.4 %)	3207 (93.2 %)	P<0.001	234 (83.0 %)	5208 (89.2 %)	0.002
	Healthy	695 (11.2 %)	461 (16.6 %)	234 (6.8 %)		48 (17.0 %)	630 (10.8 %)	
Poor Quality of sleep	Satisfactory	3810 (57.2 %)	1730 (57.3 %)	2080 (57.1 %)	0.962	173 (55.1 %)	3624 (57.3 %)	0.318
	Somewhat unsatisfactory	2295 (34.5 %)	1037 (34.4 %)	1258 (34.5 %)		109 (34.7 %)	2177 (34.4 %)	
	Significantly unsatisfactory	486 (7.3 %)	217 (7.2 %)	269 (7.4 %)		27 (8.6 %)	458 (7.2 %)	
	Totally unsatisfactory	69 (1 %)	33 (1.1 %)	36 (1.0 %)		5 (1.6 %)	62 (1.0 %)	
Anxiety	Mean (sd)	1.3 (0.3)	1.27 (0.28)	1.33 (0.31)	P<0.001	1.36 (0.33)	1.30 (0.30 %)	0.002
Depression	Mean (sd)	1.34 (0.34)	1.29 (0.31)	1.39 (0.36)	P<0.001	1.40 (0.35)	1.34 (0.34 %)	0.004
Education	less than 9 years of comprehensive school	306 (5.5 %)	152 (5.8 %)	154 (5.2 %)	0.169	19 (7.3 %)	280 (5.4 %)	0.003
	Comprehensive school	4362 (78.4 %)	2053 (78.9 %)	2309 (77.9 %)		214 (82.6 %)	4080 (78.1 %)	
	High educated	898 (16.1 %)	397 (15.3 %)	501 (16.9 %)		26 (10.0 %)	867 (16.6 %)	
Psoriasis	No	6575 (97.7 %)	2996 (97.6 %)	3579 (97.8 %)	0.702	304 (95.3 %)	6253 (97.8 %)	0.006
	Yes	156 (2.3 %)	74 (2.4 %)	82 (2.2 %)		15 (4.7 %)	140 (2.2 %)	
Pityriasis versicolor	No	1869 (98.1 %)	864 (98.0 %)	1005 (98.1 %)	0.899	94 (94.9 %)	1747 (98.2 %)	0.042
	Yes	37 (1.9 %)	18 (2.0 %)	19 (1.9 %)		5 (5.1 %)	32 (1.8 %)	
Tinea pedis	No	1392 (73 %)	557 (63.2 %)	835 (81.5 %)	P<0.001	55 (55.6 %)	1316 (74.0 %)	P<0.001
	Yes	514 (27 %)	325 (36.8 %)	189 (18.5 %)		44 (44.4 %)	463 (26.0 %)	
Onychomycosis	No	1726 (90.6 %)	757 (85.8 %)	969 (94.6 %)	P<0.001	83 (83.8 %)	1619 (91.0 %)	0.027
	Yes	180 (9.4 %)	125 (14.2 %)	55 (5.4 %)		16 (16.2 %)	160 (9.0 %)	

For peer review only

Café-au-lait spots	No	1668 (87.5 %)	776 (88.0 %)	892 (87.1 %)	0.614	96 (97.0 %)	1550 (87.1 %)	0.006
	Yes	238 (12.5 %)	106 (12.0 %)	132 (12.9 %)		3 (3.0 %)	229 (12.9 %)	
Lentigo senilis	No	1650 (86.6 %)	794 (90.0 %)	856 (83.6 %)	P<0.001	94 (94.9 %)	1531 (86.1 %)	0.018
	Yes	256 (13.4 %)	88 (10.0 %)	168 (16.4 %)		5 (5.1 %)	248 (13.9 %)	
Rosacea	No	1614 (84.8 %)	790 (89.6 %)	824 (80.6 %)	P<0.001	76 (76.8 %)	1513 (85.1 %)	0.035
	Yes	290 (15.2 %)	92 (10.4 %)	198 (19.4 %)		23 (23.2 %)	264 (14.9 %)	
Hyperhidrosis	No	1864 (97.8 %)	850 (96.4 %)	1014 (99.0 %)	P<0.001	92 (92.9 %)	1744 (98.0 %)	0.003
	Yes	42 (2.2 %)	32 (3.6 %)	10 (1.0 %)		7 (7.1 %)	35 (2.0 %)	
Type 2 diabetes	No	6660 (95.2 %)	3027 (94.4 %)	3633 (95.9 %)	0.004	-	-	-
	Yes	336 (4.8 %)	180 (5.6 %)	156 (4.1 %)		-	-	

P-value[a] for differences between male and female. P-value[b] for differences between people with diabetes and healthy people

Table 2 Standardized path coefficients

	Estimate (95% CI)	P-value
**T2D**		
SEX → T2D	0.024 (0.001, 0.046)	0.038
BMI → T2D	0.042 (0.031, 0.054)	<0.001
Poor Quality of sleep → T2D	-0.005 (-0.016, 0.006)	0.398
Psoriasis → T2D	0.109 (0.037, 0.181)	0.003
Pityriasis versicolor → T2D	0.109 (0.03, 0.189)	0.007
Tinea pedis → T2D	0.032 (0.006, 0.057)	0.014
Onychomycosis → T2D	0.033 (-0.005, 0.072)	0.090
Café-au-lait spots → T2D	-0.038 (-0.071, -0.004)	0.026
Lentigo senilis → T2D	-0.025 (-0.057, 0.007)	0.126
Rosacea → T2D	0.011 (-0.021, 0.042)	0.511
**BMI**		
Diet → BMI	0.171 (0.013, 0.329)	0.034
Anxiety → BMI	-0.008 (-0.089, 0.074)	0.856
Depression → BMI	0.033 (-0.046, 0.113)	0.413
**Diet**		
Education → Diet	-0.039 (-0.057, -0.021)	<0.001
**Hyperhidrosis**		
Diet → Hyperhidrosis	0.003 (-0.021, 0.027)	0.812
Anxiety → Hyperhidrosis	0.005 (-0.003, 0.013)	0.251
**Poor Quality of sleep**		
Bmi → Poor Quality of sleep	0.065 (0.011, 0.118)	0.017
Anxiety → Poor Quality of sleep	0.077 (-0.005, 0.159)	0.064
Depression → Poor Quality of sleep	0.095 (0.015, 0.175)	0.020
**Tinea pedis**		
Hyperhidrosis → Tinea pedis	0.175 (0.018, 0.332)	0.029
**Chi-square goodness fit test**	324.38	<0.001
**DF**	55	
**CMIN/DF**	5.89	
**RMSEA**	0.060	
**SRMR**	0.045	

Note that: T2D: Type 2 diabetes; BMI: body mass index; SE: Standard Error; RMSEA: root mean square error approximation; SRMR: standardized root mean square residual. Variables listed in the above are standardized variables that are transformed version of an original variable that have been adjusted to have a mean of 0 and a standard deviation of 1

For peer review only

Table 1 Descriptive characteristics of the study population

Variable	Category	Overall	Men	Women	P-value[a]	Type 2 diabetes	healthy	P-value[b]
Tinea pedis	No	1392 (73.0 %)	557 (63.2 %)	835 (81.5 %)	<0.001	55 (55.6 %)	1316 (74.0 %)	<0.001
	Yes	514 (27.0 %)	325 (36.8 %)	189 (18.5 %)		44 (44.4 %)	463 (26.0 %)	
Hyperhidrosis	No	1864 (97.8 %)	850 (96.4 %)	1014 (99.0 %)	<0.001	92 (92.9 %)	1744 (98.0 %)	0.003
	Yes	42 (2.2 %)	32 (3.6 %)	10 (1.0 %)		7 (7.1 %)	35 (2.0 %)	
Café-au-lait spots	No	1668 (87.5 %)	776 (88.0 %)	892 (87.1 %)	0.614	96 (97.0 %)	1550 (87.1 %)	0.006
	Yes	238 (12.5 %)	106 (12.0 %)	132 (12.9 %)		3 (3.0 %)	229 (12.9 %)	
Pigmented nevus	No	1685 (88.4 %)	766 (86.8 %)	919 (89.7 %)	0.058	88 (88.9 %)	1572 (88.4 %)	1
	Yes	221 (11.6 %)	116 (13.2 %)	105 (10.3 %)		11 (11.1 %)	207 (11.6 %)	
Lentigo senilis	No	1650 (86.6 %)	794 (90.0 %)	856 (83.6 %)	<0.001	94 (94.9 %)	1531 (86.1 %)	0.018
	Yes	256 (13.4 %)	88 (10.0 %)	168 (16.4 %)		5 (5.1 %)	248 (13.9 %)	
Psoriasis skin symptoms	No	1862 (97.9 %)	856 (97.4 %)	1006 (98.3 %)	0.198	93 (94.9 %)	1741 (98.0 %)	0.084
	Yes	40 (2.1 %)	23 (2.6 %)	17 (1.7 %)		5 (5.1 %)	35 (2.0 %)	
Psoriasis nail symptoms	No	1889 (99.4 %)	869 (98.9 %)	1020 (99.8 %)	0.022	98 (100.0 %)	1763 (99.3 %)	0.868
	Yes	12 (0.6 %)	10 (1.1 %)	2 (0.2 %)		0 (0.0 %)	12 (0.7 %)	
Psoriasis joint symptoms	No	1888 (99.3 %)	874 (99.4 %)	1014 (99.2 %)	0.775	97 (99.0 %)	1763 (99.3 %)	1
	Yes	13 (0.7 %)	5 (0.6 %)	8 (0.8 %)		1 (1.0 %)	12 (0.7 %)	
Psoriasis	No	6575 (97.7 %)	2996 (97.6 %)	3579 (97.8 %)		304 (95.3 %)	6253 (97.8 %)	
	Yes	156 (2.3 %)	74 (2.4 %)	82 (2.2 %)	0.702	15 (4.7 %)	140 (2.2 %)	0.006
Rosacea	No	1614 (84.8 %)	790 (89.6 %)	824 (80.6 %)	<0.001	76 (76.8 %)	1513 (85.1 %)	0.035
	Yes	290 (15.2 %)	92 (10.4 %)	198 (19.4 %)		23 (23.2 %)	264 (14.9 %)	
Stasis dermatitis	No	1890 (99.2 %)	874 (99.1 %)	1016 (99.3 %)	0.773	97 (98.0 %)	1765 (99.3 %)	0.411
	Yes	15 (0.8 %)	8 (0.9 %)	7 (0.7 %)		2 (2.0 %)	13 (0.7 %)	
Bluenevus	No	1880 (98.6 %)	874 (99.1 %)	1006 (98.2 %)	0.162	97 (98.0 %)	1755 (98.7 %)	0.909
	Yes	26 (1.4 %)	8 (0.9 %)	18 (1.8 %)		2 (2.0 %)	24 (1.3 %)	
Nevus spilus	No	1817 (95.3 %)	843 (95.6 %)	974 (95.1 %)	0.714	98 (99.0 %)	1691 (95.1 %)	0.121
	Yes	89 (4.7 %)	39 (4.4 %)	50 (4.9 %)		1 (1.0 %)	88 (4.9 %)	
Halo nevus	No	1891 (99.2 %)	876 (99.3 %)	1015 (99.1 %)	0.819	99 (100.0 %)	1764 (99.2 %)	0.736
	Yes	15 (0.8 %)	6 (0.7 %)	9 (0.9 %)		0 (0.0 %)	15 (0.8 %)	
Dermatofibroma	No	1481 (77.7 %)	730 (82.8 %)	751 (73.3 %)	<0.001	71 (71.7 %)	1390 (78.1 %)	0.17
	Yes	425 (22.3 %)	152 (17.2 %)	273 (26.7 %)		28 (28.3 %)	389 (21.9 %)	
Cherry angioma	No	759 (39.8 %)	410 (46.5 %)	349 (34.1 %)	<0.001	44 (44.4 %)	705 (39.7 %)	0.4
	Yes	1146 (60.2 %)	472 (53.5 %)	674 (65.9 %)		55 (55.6 %)	1073 (60.3 %)	

For peer review only

Cheloid	No	1857 (97.4 %)	859 (97.4 %)	998 (97.5 %)	1	95 (96.0 %)	1735 (97.5 %)	0.526
	Yes	49 (2.6 %)	23 (2.6 %)	26 (2.5 %)		4 (4.0 %)	44 (2.5 %)	
Seborrheic keratosis	No	1061 (55.7 %)	522 (59.2 %)	539 (52.6 %)	0.005	58 (58.6 %)	987 (55.5 %)	0.616
	Yes	845 (44.3 %)	360 (40.8 %)	485 (47.4 %)		41 (41.4 %)	792 (44.5 %)	
Lipoma	No	1845 (96.8 %)	841 (95.4 %)	1004 (98.0 %)	0.001	94 (94.9 %)	1726 (97.0 %)	0.389
	Yes	61 (3.2 %)	41 (4.6 %)	20 (2.0 %)		5 (5.1 %)	53 (3.0 %)	
Congenital nevus	No	1828 (95.9 %)	838 (95.0 %)	990 (96.7 %)	0.086	98 (99.0 %)	1703 (95.7 %)	0.183
	Yes	78 (4.1 %)	44 (5.0 %)	34 (3.3 %)		1 (1.0 %)	76 (4.3 %)	
Nevus flammeus	No	1419 (74.4 %)	690 (78.2 %)	729 (71.2 %)	0.001	79 (79.8 %)	1316 (74.0 %)	0.241
	Yes	487 (25.6 %)	192 (21.8 %)	295 (28.8 %)		20 (20.2 %)	463 (26.0 %)	
Actinic keratosis	No	1894 (99.4 %)	875 (99.2 %)	1019 (99.5 %)	0.582	99 (100.0 %)	1767 (99.3 %)	0.864
	Yes	12 (0.6 %)	7 (0.8 %)	5 (0.5 %)		0 (0.0 %)	12 (0.7 %)	
Atopic dermatitis	No	1813 (95.2 %)	847 (96.0 %)	966 (94.4 %)	0.128	97 (98.0 %)	1689 (95.0 %)	0.269
	Yes	92 (4.8 %)	35 (4.0 %)	57 (5.6 %)		2 (2.0 %)	89 (5.0 %)	
Hand eczema	No	1736 (91.1 %)	817 (92.6 %)	919 (89.8 %)	0.039	95 (96.0 %)	1614 (90.8 %)	0.115
	Yes	169 (8.9 %)	65 (7.4 %)	104 (10.2 %)		4 (4.0 %)	164 (9.2 %)	
Seborrheic dermatitis	No	1765 (92.7 %)	774 (87.8 %)	991 (96.9 %)	<0.001	90 (90.9 %)	1650 (92.8 %)	0.613
	Yes	140 (7.3 %)	108 (12.2 %)	32 (3.1 %)		9 (9.1 %)	128 (7.2 %)	
Nummular dermatitis	No	1869 (98.1 %)	858 (97.3 %)	1011 (98.8 %)	0.021	96 (97.0 %)	1746 (98.2 %)	0.618
	Yes	36 (1.9 %)	24 (2.7 %)	12 (1.2 %)		3 (3.0 %)	32 (1.8 %)	
Infectious eczema	No	1878 (98.6 %)	862 (97.7 %)	1016 (99.3 %)	0.007	96 (97.0 %)	1754 (98.7 %)	0.351
	Yes	27 (1.4 %)	20 (2.3 %)	7 (0.7 %)		3 (3.0 %)	24 (1.3 %)	
Contact dermatitis	No	1887 (99.1 %)	880 (99.8 %)	1007 (98.4 %)	0.006	99 (100.0 %)	1761 (99.0 %)	0.665
	Yes	18 (0.9 %)	2 (0.2 %)	16 (1.6 %)		0 (0.0 %)	17 (1.0 %)	
Neurodermatitis	No	1871 (98.2 %)	857 (97.2 %)	1014 (99.1 %)	0.002	95 (96.0 %)	1748 (98.3 %)	0.186
	Yes	34 (1.8 %)	25 (2.8 %)	9 (0.9 %)		4 (4.0 %)	30 (1.7 %)	
Lichen planus	No	1893 (99.3 %)	874 (99.1 %)	1019 (99.5 %)	0.407	99 (100.0 %)	1766 (99.3 %)	0.817
	Yes	13 (0.7 %)	8 (0.9 %)	5 (0.5 %)		0 (0.0 %)	13 (0.7 %)	
Dermatitis herpetiformis	No	1900 (99.7 %)	880 (99.8 %)	1020 (99.6 %)	0.821	99 (100.0 %)	1773 (99.7 %)	1
	Yes	6 (0.3 %)	2 (0.2 %)	4 (0.4 %)		0 (0.0 %)	6 (0.3 %)	
Vitiligo	No	1874 (98.3 %)	872 (98.9 %)	1002 (97.9 %)	0.123	98 (99.0 %)	1748 (98.3 %)	0.881
	Yes	32 (1.7 %)	10 (1.1 %)	22 (2.1 %)		1 (1.0 %)	31 (1.7 %)	
Urticaria	No	1896 (99.5 %)	877 (99.4 %)	1019 (99.5 %)	1	98 (99.0 %)	1770 (99.5 %)	1
	Yes	10 (0.5 %)	5 (0.6 %)	5 (0.5 %)		1 (1.0 %)	9 (0.5 %)	
Acne	No	1755 (92.1 %)	818 (92.7 %)	937 (91.6 %)	0.399	94 (94.9 %)	1635 (92.0 %)	0.377

For peer review only

	Yes	150 (7.9 %)	64 (7.3 %)	86 (8.4 %)		5 (5.1 %)	143 (8.0 %)	
Perioral dermatitis	No	1888 (99.3 %)	881 (99.9 %)	1007 (98.7 %)	0.007	98 (99.0 %)	1762 (99.3 %)	1
	Yes	14 (0.7 %)	1 (0.1 %)	13 (1.3 %)		1 (1.0 %)	13 (0.7 %)	
Acne scars	No	1716 (90.2 %)	780 (88.5 %)	936 (91.7 %)	0.026	89 (90.8 %)	1602 (90.2 %)	0.981
	Yes	186 (9.8 %)	101 (11.5 %)	85 (8.3 %)		9 (9.2 %)	174 (9.8 %)	
Pityriasis versicolor	No	1869 (98.1 %)	864 (98.0 %)	1005 (98.1 %)	0.9	94 (94.9 %)	1747 (98.2 %)	0.058
	Yes	37 (1.9 %)	18 (2.0 %)	19 (1.9 %)		5 (5.1 %)	32 (1.8 %)	
Onychomycosis	No	1726 (90.6 %)	757 (85.8 %)	969 (94.6 %)	<0.001	83 (83.8 %)	1619 (91.0 %)	0.027
	Yes	180 (9.4 %)	125 (14.2 %)	55 (5.4 %)		16 (16.2 %)	160 (9.0 %)	
Tinea corporis	No	1898 (99.6 %)	878 (99.5 %)	1020 (99.6 %)	1	98 (99.0 %)	1772 (99.6 %)	0.901
	Yes	8 (0.4 %)	4 (0.5 %)	4 (0.4 %)		1 (1.0 %)	7 (0.4 %)	
Folliculitis	No	1790 (93.9 %)	796 (90.2 %)	994 (97.1 %)	<0.001	95 (96.0 %)	1667 (93.7 %)	0.488
	Yes	116 (6.1 %)	86 (9.8 %)	30 (2.9 %)		4 (4.0 %)	112 (6.3 %)	
Plantar warts	No	1732 (90.9 %)	809 (91.7 %)	923 (90.1 %)	0.263	94 (94.9 %)	1613 (90.7 %)	0.207
	Yes	174 (9.1 %)	73 (8.3 %)	101 (9.9 %)		5 (5.1 %)	166 (9.3 %)	
Hand warts	No	1855 (97.3 %)	860 (97.5 %)	995 (97.2 %)	0.754	96 (97.0 %)	1731 (97.3 %)	1
	Yes	51 (2.7 %)	22 (2.5 %)	29 (2.8 %)		3 (3.0 %)	48 (2.7 %)	
Alopecia areata	No	1899 (99.6 %)	877 (99.4 %)	1022 (99.8 %)	0.338	99 (100.0 %)	1773 (99.7 %)	1
	Yes	7 (0.4 %)	5 (0.6 %)	2 (0.2 %)		0 (0.0 %)	6 (0.3 %)	
Male pattern baldness	No	1291 (67.7 %)	280 (31.7 %)	1011 (98.7 %)	<0.001	69 (69.7 %)	1202 (67.6 %)	0.741
	Yes	615 (32.3 %)	602 (68.3 %)	13 (1.3 %)		30 (30.3 %)	577 (32.4 %)	
Other skin disease	No	1595 (90.2 %)	753 (91.2 %)	842 (89.4 %)	0.453	74 (82.2 %)	1497 (90.6 %)	0.021
	Yes	1685 (88.4 %)	71 (8.6 %)	97 (10.3 %)		16 (17.8 %)	150 (9.1 %)	
	Perhaps	221 (11.6 %)	2 (0.2 %)	3 (0.3 %)		0 (0.0 %)	5 (0.3 %)	
Using sunscreen inland	Not at all	1068 (15.9 %)	759 (24.7 %)	309 (8.5 %)	<0.001	75 (23.2 %)	987 (15.5 %)	<0.001
	Sometimes	3170 (47.1 %)	1600 (52.1 %)	1570 (43.0 %)		150 (46.4 %)	3004 (47.1 %)	
	Reqularly	2210 (32.9 %)	624 (20.3 %)	1586 (43.4 %)		78 (24.1 %)	2128 (33.4 %)	
	I do not spend time in the sun	279 (4.1 %)	89 (2.9 %)	190 (5.2 %)		20 (6.2 %)	259 (4.1 %)	
Using sunscreen in abroad	Not at all	960 (15.1 %)	640 (22.1 %)	320 (9.3 %)	<0.001	71 (23.1 %)	885 (14.7 %)	<0.001
	Sometimes	574 (9.0 %)	367 (12.7 %)	207 (6.0 %)		28 (9.1 %)	542 (9.0 %)	
	Reqularly	4079 (64.3 %)	1620 (55.9 %)	2459 (71.4 %)		166 (54.1 %)	3900 (64.8 %)	
	I do not spend time in the sun	732 (11.5 %)	272 (9.4 %)	460 (13.3 %)		42 (13.7 %)	690 (11.5 %)	

P-value[a] for differences between male and female. P-value[b] for differences between people with diabetes and healthy.

For peer review only

Table 2 Agreement coefficient between different methods

Method1	Method2	Cohen kappa (95% CI)	P_value
Ridge	Lasso	0.24 (-0.043, 0.53)	0.094
Ridge	Univariate analysis	0.16 (-0.122, 0.448)	0.248
Ridge	BSLM	0.23 (-0.058, 0.511)	0.12
Ridge	MCP	0.28 (0.002, 0.56)	0.053
Ridge	Sparse step	0.05 (-0.233, 0.342)	0.708
Lasso	Univariate analysis	0.53 (0.27, 0.799)	<0.001
Lasso	BSLM	0.86 (0.709, 1)	<0.001
Lasso	MCP	0.82 (0.649, 0.986)	<0.001
Lasso	Sparse step	0.71 (0.488, 0.924)	<0.001
Univariate analysis	BSLM	0.42 (0.159, 0.687)	0.002
Univariate analysis	MCP	0.39 (0.128, 0.651)	0.005
Univariate analysis	Sparse step	0.43 (0.151, 0.711)	0.003
BSLM	MCP	0.96 (0.869, 1)	<0.001
BSLM	Sparse step	0.77 (0.577, 0.958)	<0.001
MCP	Sparse step	0.73 (0.524, 0.927)	<0.001

For peer review only

Table 3 Variance inflation factor for selected variables

Variable	VIF
BMI	1.08
Diet	1.04
Poor Quality of sleep	1.06
Anxiety	2.38
Depression	2.38
Education	1.06
Psoriasis	1.03
Pityriasis versicolor	1.02
Tinea pedis	1.16
Onychomycosis	1.12
Café_au_lait_spots	1.01
Lentigo senilis	1.01
Rosacea	1.04
Hyperhidrosis	1.05

For peer review only

Table 4 Exponentiated Path coefficient analysis

Independent Variable	Total Effect	Direct Effect	Indirect Effect
Sex	1.024 (1.001, 1.047)	1.024 (1.001, 1.047)	-
BMI	1.043 (1.031, 1.056)	1.043 (1.031, 1.055)	1 (1, 1.001)
Diet	1 (1, 1.001)	-	1 (1, 1.001)
Poor Quality of sleep	0.995 (0.984, 1.006)	0.995 (0.984, 1.006)	-
Anxiety	0.999 (0.997, 1.005)	-	0.999 (0.997, 1.005)
Depression	1 (1, 1.001)	-	1 (1, 1.001)
Education	1 (1, 1)	-	1 (1, 1)
Psoriasis	1.115 (1.038, 1.198)	1.115 (1.038, 1.198)	-
Pityriasis versicolor	1.115 (1.03, 1.208)	1.115 (1.03, 1.208)	-
Tinea pedis	1.033 (1.006, 1.059)	1.033 (1.006, 1.059)	-
Onychomycosis	1.034 (0.995, 1.075)	1.034 (0.995, 1.075)	-
Café-au-lait spots	0.963 (0.931, 0.996)	0.963 (0.931, 0.996)	-
Lentigo senilis	0.975 (0.945, 1.007)	0.975 (0.945, 1.007)	-
Rosacea	1.011 (0.979, 1.043)	1.011 (0.979, 1.043)	-
Hyperhidrosis	1.006 (1, 1.019)	-	1.006 (1, 1.019)

For peer review only

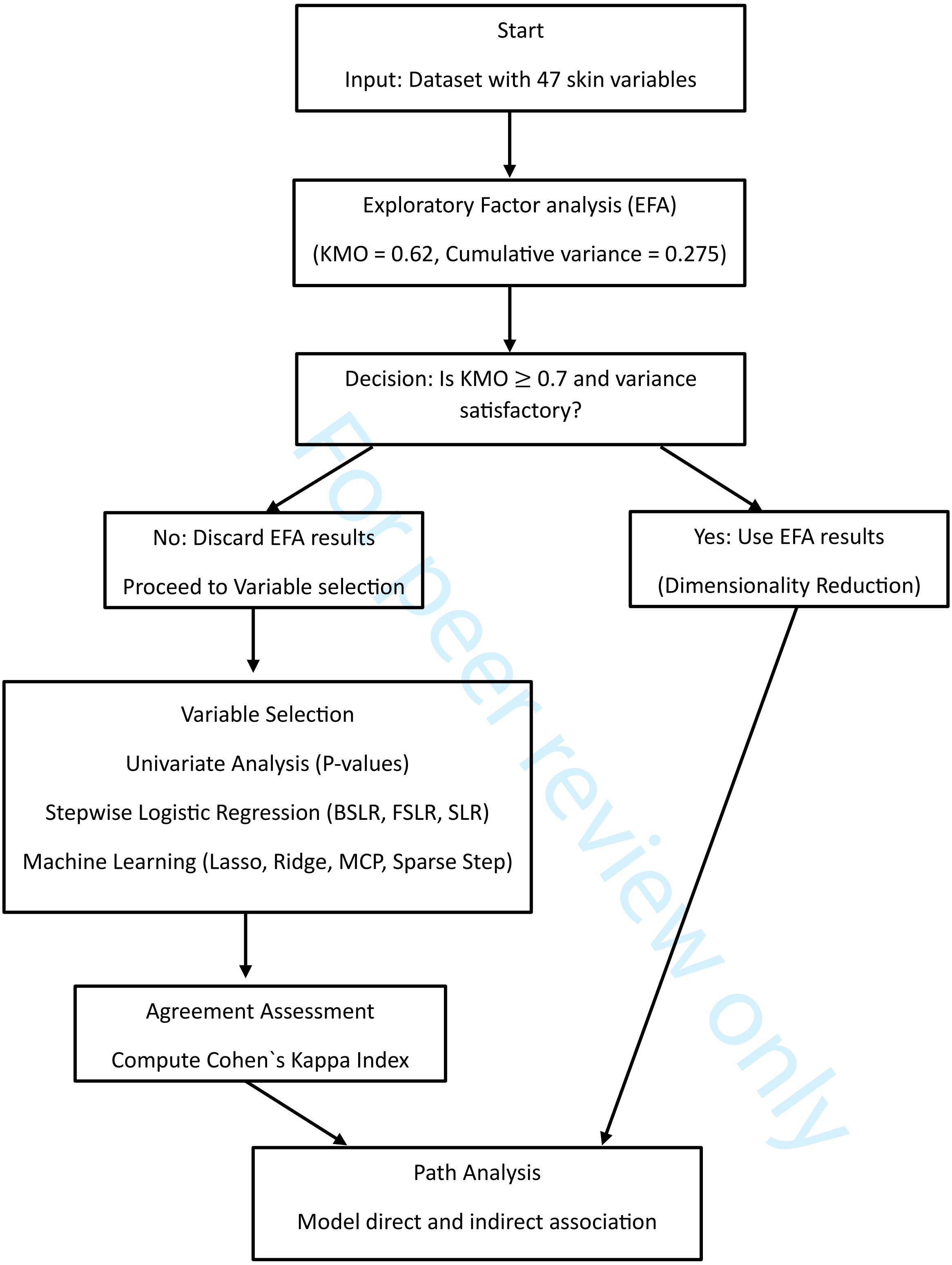

Figure 1 Flow chart demonstrating the analytical plan

For peer review only

BMJ Open

Assessing the association of Type 2 diabetes with skin health status: A study of the Northen Finland Birth Cohort 1966

Journal:	*BMJ Open*
Manuscript ID	bmjopen-2025-109709.R1
Article Type:	Original research
Date Submitted by the Author:	31-Mar-2026
Complete List of Authors:	Nasserinejad, Maryam; University of Oulu, Research Unit of Population Health, Faculty of Medicine<br>Huilaja, Laura; University of Oulu, Research Unit of Clinical Medicine, Medical Research Center<br>Sinikumpu, Suvi-Päivikki; University of Oulu, Research Unit of Clinical Medicine, Medical Research Center<br>Vainio, Seppo ; University of Oulu, Faculty of Biochemistry and Molecular Medicine, Disease Networks, Laboratory of Developmental Biology<br>Röning, Juha ; University of Oulu, Biomimetics and Intelligent Systems Group, Faculty of Information Technology and Electrical Engineering<br>Ali, Nsrein ; University of Oulu, Faculty of Biochemistry and Molecular Medicine, Disease Networks, Laboratory of Developmental Biology; University of Oulu, Research Unit of Health Sciences and Technology. Faculty of Medicine<br>Sebert, Sylvain; University of Oulu, Research Unit of Population Health, Faculty of Medicine
**Primary Subject Heading**:	Diabetes and endocrinology
Secondary Subject Heading:	Dermatology
Keywords:	Dermatology < INTERNAL MEDICINE, Public health < INFECTIOUS DISEASES, General diabetes < DIABETES & ENDOCRINOLOGY

SCHOLARONE™ MANUSCRIPTS

I, the Submitting Author has the right to grant and does grant on behalf of all authors of the Work (as defined in the below author licence), an exclusive licence and/or a non-exclusive licence for contributions from authors who are: i) UK Crown employees; ii) where BMJ has agreed a CC-BY licence shall apply, and/or iii) in accordance with the terms applicable for US Federal Government officers or employees acting as part of their official duties; on a worldwide, perpetual, irrevocable, royalty-free basis to BMJ Publishing Group Ltd ("BMJ") its licensees and where the relevant Journal is co-owned by BMJ to the co-owners of the Journal, to publish the Work in this journal and any other BMJ products and to exploit all rights, as set out in our licence.

The Submitting Author accepts and understands that any supply made under these terms is made by BMJ to the Submitting Author unless you are acting as an employee on behalf of your employer or a postgraduate student of an affiliated institution which is paying any applicable article publishing charge ("APC") for Open Access articles. Where the Submitting Author wishes to make the Work available on an Open Access basis (and intends to pay the relevant APC), the terms of reuse of such Open Access shall be governed by a Creative Commons licence – details of these licences and which Creative Commons licence will apply to this Work are set out in our licence referred to above.

Other than as permitted in any relevant BMJ Author's Self Archiving Policies, I confirm this Work has not been accepted for publication elsewhere, is not being considered for publication elsewhere and does not duplicate material already published. I confirm all authors consent to publication of this Work and authorise the granting of this licence.

For peer review only

Assessing the association of Type 2 diabetes with skin health status: A study of the Northen Finland Birth Cohort 1966

Maryam Nasserinejad [1]✉ Laura Huilaja,[2] Suvi-Päivikki Sinikumpu,[2] Seppo Vainio,[3] Juha Röning,[4,5] Nsrein Ali,[3,6] Sylvain Sebert[1]

[1] Research Unit of Population Health, Faculty of Medicine, University of Oulu, Oulu, Finland. [2] Research Unit of Clinical Medicine, Medical Research Center, University of Oulu and Department of Dermatology, Oulu University Hospital, Oulu, Finland. [3] Faculty of Biochemistry and Molecular Medicine, Disease Networks, Laboratory of Developmental Biology, University of Oulu, Oulu, Finland. [4] Biomimetics and Intelligent Systems Group, Faculty of Information Technology and Electrical Engineering, University of Oulu, 90014 Oulu, Finland. [5] Symbiosis Institute of Technology, Symbiosis International (Deemed University) India. [6] Research Unit of Health Sciences and Technology. Faculty of Medicine, 90220Oulu. Oulu Finland. ✉ email: maryam.nasserinejad@oulu.fi

For peer review only

For peer review only - http://bmjopen.bmj.com/site/about/guidelines.xhtml

Abstract

Background: There is evidence to suggest that alterations in skin health often co-occur with T2D. However, the underlying mechanisms remain unclear. Despite their clinical relevance, skin conditions are often underdiagnosed in diabetes care. This study applied variable selection methods to identify key dermatological factors linked to T2D and examined their direct and indirect associations.

Methods: This study analysed data from the Northern Finland Birth Cohort 1966 (NFBC1966), including 1,930 participants (1,036 women, 894 men) at ages 31y and 46y. At age 46y the cohort participants, living in the region of Northern Finland, were invited to a general health clinical examination including the assessment of their T2D status and a dermatological examination. Prior to performing a path analysis, we developed a data driven procedure to select 47 possible skin-related variables into the model. The variable selection combined test-based methods (i.e. a univariate analysis, and a backward, forward, and stepwise logistic regression) and penalty-based/machine learning methods (i.e. Lasso, Ridge, minimax convex penalty, and Sparse Stepwise Regression). The selected skin-related variables were then included in a path analyses accounting for sex, body mass index (BMI), diet, education, depression, anxiety and sleep quality.

Results:

Eight skin-related variables were selected and included in the path analysis. Psoriasis (Odds Raito (OR) 1.115, 95% CI 1.038–1.198, p = 0.033) and Tinea pedis (OR 1.033, 95% CI 1.006–1.059, p = 0.014) showed direct associations with T2D. Hyperhidrosis (OR 1.175, 95% CI 1.018–1.394, p = 0.029) exhibited an indirect effect.

Conclusion: These results underscored the interconnectedness of dermatological conditions such as psoriasis and Tinea pedis with T2D. These findings suggest that certain skin conditions may serve as potential indicators of T2D. Early recognition and appropriate management of these conditions may help improve patients' quality of life.

Strengths and limitations of this study:

For peer review only - http://bmjopen.bmj.com/site/about/guidelines.xhtml

- Dermatological examinations were diagnosed by dermatologists, enhancing the accuracy and validity of the data.
- Use of path analyses to examine direct and indirect associations while adjusting for key confounders.
- Applied multiple statistical and machine learning methods for variable selection to improve reliability.
- A community-based study with limited sample size may introduce selection factors and restrict generalisability to the broader Finnish population.
- Data were collected at a single time point, preventing a longitudinal assessment and causal inference.

For peer review only

For peer review only - http://bmjopen.bmj.com/site/about/guidelines.xhtml

Introduction

Type 2 diabetes (T2D) is one main segments of chronic non-communicable diseases characterised by elevated levels of blood glucose and is associated with serious complications to the heart, blood vessels, digestive system, eyes, kidneys, nerves, skin, and even psychosocial aspects and early mortality [1].

An estimated 537 million adults aged 20–79 years worldwide (10.5% of all adults in this age group) had diabetes in 2021. By 2030, this number is projected to rise to 643 millions, and by 2045, it could reach 783 millions. This would represent a 46% increase in the number of people living with diabetes, while the global population is estimated to grow by only 20% over the same period [2]. According to the World Health Organisation, in the European Region alone, approximately 64 millions of adults and around 300,000 children and adolescents are living with diabetes as of 2024, making it one of the most common chronic conditions in Europe [3]. According to the latest reports, among the European Nordic countries, Finland has the highest prevalence of diabetes, at 9.7%. The number of adults with diabetes is estimated to be 392.9 thousand (95% CI: 334.8,435.9) [2].

The burden of and the premature mortality attributed to T2D are very high, and most people living with T2D are reported to suffer other complications leading to a dramatic reduction in their quality of life [4]. The comorbidity of skin disease and T2D is undeniable, and almost one third of people with diabetes have skin lesions [5].

Numerous studies have shown associations between different types of skin disorders with T2D. For example, substantial evidence supports a link between inflammatory diseases, such as psoriasis and eczema, and T2D [6][7]. In addition, Azfar et *al.* proposed previous incidence of psoriasis as a risk factor for the development of T2D [8]. Other skin conditions associated with T2D, such as acanthosis nigricans, diabetic dermopathy, necrobiosis lipoidica, bacterial and fungal infections, and xerosis (dry skin), often appear early, sometimes before T2D is diagnosed. These skin disorders may not only reflect the systemic effects of diabetes but can also serve as

For peer review only

For peer review only - http://bmjopen.bmj.com/site/about/guidelines.xhtml

early indicators, emphasising the importance of timely recognition and management to improve the prevention and/or reduce the burden of T2D [9][10].

It is currently hypothesised that a large number of skin-related conditions may be directly associated with T2D either through causal mechanisms or psycho-social pathways. This study was designed to identify the most relevant dermatological conditions associated with T2D in the general population living in Northern Finland to examine possible direct and indirect relationships between skin health and T2D, while adjusting for potential confounders and covariates such as diet, education, BMI, anxiety, depression, sleep quality, and sex.

Materials and methods

Overview and data resources

The selected population

The current study is derived from a longitudinal birth cohort study and used data from the participants of the Northern Finland Birth Cohort 1966 (NFBC1966) [11]. The NFBC1966 is a longitudinal population-based study initiated to investigate factors affecting health and development from prenatal stages into adulthood. The cohort comprised 12,138 live births from 1966, representing 96.3% of all births that year in Finland's two northernmost provinces, Oulu and Lapland [12]. The selection process to define the selected study participants from the eligible population is shown in the study flowchart (Fig. 1). The study included all eligible participants with available follow-up data on their skin health status and T2D defined at age 46 years (in 2012) and all available covariates. Due to logistic and financial constraints, the clinical examination for dermatology diagnosis was only performed for participants living in the region of Oulu and was conducted at the University Hospital of Oulu. It included a total of 894 males and 1,036 females (N = 1,930). Missing data were handled using listwise deletion, whereby participants with missing values in any of the variables included in a given model were excluded from that specific analysis. The number of missing observations for each variable is presented in Supplementary Table S1. The studies received ethical approval from Northern Ostrobothnia Hospital District Ethical Committee 94/2011 (12.12.2011).

For peer review only - http://bmjopen.bmj.com/site/about/guidelines.xhtml

Patient and public involvement

Neither patient groups nor participants in the study were involved in the design, conduct, reporting, or dissemination plans of this research.

Exposure variables: skin health status variables

Each individual skin health status could be defined from 46 (Supplementary Table S1) different variables. These variables, also referred to as measures, were part of a clinical protocol for a comprehensive dermatological assessment, designed by dermatologist, to study the prevalence of major skin diseases and disorders in mid-life [13]. All variables were collected through clinical examinations, except for doctor-diagnosed psoriasis, which was obtained via a postal questionnaire, and two variables related to sunscreen use, which were self-reported by the participants.

Outcome variable: T2D

All T2D-related data were obtained from the 46-year follow-up of the NFBC1966 cohort. We defined cases with T2D according to the following criteria depending on their level of participation or prior status for T2D. All eligible participants were invited to an oral glucose tolerance test (OGTT) with a 75g anhydrous glucose ingestion. From this test, we classified individuals with T2D (N cases = 267) if the fasting OGTT value was 7 mmol/l or higher, and the value at two hours-post OGTT was 11.1 mmol/l or greater. Additionally, cases with T2D (N cases = 192) where included according to self-reports with the following question: "Do you currently have or have you ever had adult-onset diabetes (type 2)?" Finaly, we included additional cases should they report the use of blood sugar-lowering medications like metformin (N cases = 102). Some participants met more than one of the above criteria, resulting in overlap between the groups. The total number of unique individuals with T2D was estimated to be 336.

This comprehensive methodology ensured a robust identification process for individuals with T2D, incorporating both clinical measurements and self-reported information.

For peer review only - http://bmjopen.bmj.com/site/about/guidelines.xhtml

Covariates

We consider the following covariates to include in the model possible covariation with BMI, sleep quality and the long-term impact of diet, education, anxiety, and depression. We employed data from 31 year in this study.

Diet was categorised as a binary variable (healthy vs. unhealthy) based on consumption patterns of vegetables, fruits, rye, and processed foods [14]. Education was defined by a combination of two different questions concerning basic education and vocational qualifications with three levels from basic educated to highly educated.

For defining anxiety and depression, we followed the Hopkins symptom checklist [15]. This questionnaire consists of 14 questions aimed at diagnosing anxiety, focusing on symptoms such as pain, tension, restlessness, trembling, fear, and heart pounding. Additionally, there are 13 questions designed to assess depression, covering issues such as difficulty falling asleep, feelings of loneliness and worthlessness, sexual difficulties, lack of energy, and suicidal thoughts. The questions were presented in a four-point Likert format, with one being 'not at all' and four being 'very much'. The depression and anxiety scores were derived from the mean scores, which ranged slightly from 1 to 4, where 1 indicated no anxiety or depression and 4 indicated a high level of anxiety or depression.

Information on sleep quality was collected by questionnaire at age 46 years.

Statistical methods

Descriptive statistics, including counts and percentages for all variables, were computed overall and stratified by sex (male and female) and T2D status (healthy and T2D groups). Chi-square tests were used to compare categorical variables between sex groups and between health status groups as presented. Initially, we applied an exploratory factor analysis (EFA) to reduce the dimensions of the skin status variables, aiming to derive components from the pool of 47 skin health status. However, due to the Kaiser–Meyer–Olkin (KMO) index of 0.62 and a low cumulative

For peer review only - http://bmjopen.bmj.com/site/about/guidelines.xhtml

variance (0.275 out of 1.00) for just the first 22 components, we opted against utilising the outcomes of this model. We opted for an alternative approach to reducing the dimensionality through variable selection.

Variable selection

Variable selection is a crucial step in inferential modelling, as accurately identifying the subset of variables truly associated with the outcome of interest is essential. General approaches to variable selection fall into three main categories: test-based, penalty-based, and screening-based methods [16]. While various techniques for variable selection exist, each has distinct mathematical properties, which can lead to differing outcomes in terms of the variables selected. This raises an important challenge for researchers: determining how the choice of selection method influences the study results and their reproducibility across different methods. Investigating the “between-method” variability in variable selection is, therefore, an important area of study.

Best practices suggest employing multiple methods and assigning confidence to variables based on their consistency across methods. In this study, we employed a variety of methods to identify the most appropriate variables for path analysis modelling, including a univariate analysis based on p-values, as well as a backward stepwise logistic regression (BSLR), forward stepwise logistic regression (FSLR), and stepwise logistic regression (SLR). Additionally, we utilised machine learning techniques, specifically least absolute shrinkage and selection operator regression (Lasso), Ridge regression, minimax convex penalty (MCP), and Sparse Stepwise Regression. The degree of agreement between the results of the various methods was assessed using Cohen’s Kappa index, which ranges from -1 to 1. Values less than 0 indicate poor agreement (worse than chance), A Kappa value below 0 indicates poor agreement (worse than chance), while values between 0.61 and 0.80 suggest substantial agreement, and values above 0.80 indicate almost perfect agreement [17]. The multicollinearity of the selected variables was

For peer review only

For peer review only - http://bmjopen.bmj.com/site/about/guidelines.xhtml

assessed using the variance inflation factor (VIF), with a maximum VIF exceeding 10 indicating potential influence of multicollinearity on the least squares estimates [18].

Path analysis

Following the selection of skin health status, we employed a path analysis to examine both direct and indirect associations, while controlling for the effects of sex, body mass index (BMI), diet, education, depression, and anxiety (Supplementary Fig. S1).

In this study, variables were standardised to facilitate comparison and to eliminate variation arising from differences in scale. The standardisation transformed the original variables so they would have a mean of 0 and a standard deviation of 1, making comparisons between variables with different scales or units more straightforward.

The model fit was evaluated using the root mean square error of approximation (RMSEA), the standardised root mean square residual (SRMR), and the relative chi-square (Chi-square minimum discrepancy (CMIN)/Degrees of Freedom (DF)). Values of RMSEA and SRMR below 0.08 and CMIN/DF below 5 were considered indicative of an acceptable model fit, based on established recommendations in the structural equation modelling literature [19].

All analyses were conducted using R software, version 4.4.0. specifically the glmnet package for Lasso and Ridge regression, ncvreg package for MCP method, sparsestep [20] for sparse step method and the lavaan package for path analysis.

For peer review only

Results

Among the study participants, the overall prevalence of T2D was 4.8%, with a significantly higher rate observed in men (5.6%) compared to women (4.1%). The prevalence of tinea pedis was 27%, with a higher prevalence in men than in women. Specifically, among patients with T2D, the prevalence of tinea pedis increased to 44.4%, with 55.5% in men and 35.1% in women (Table 1). Psoriasis was reported by 2.3% of the participants overall, with a higher prevalence in patients with T2D at 4.7%. In contrast with tinea pedis, this prevalence was notably twice as high in women (6%) compared to men (3.5%). Rosacea affected 15.2% of the participants, and among those with T2D, the prevalence rose to 23.2%, showing a significant gender disparity with 8.8% in men and 35.2% in women (Table 1). Onychomycosis was observed in 9.4% of the participants. Café-au-lait spots were found in 12.5% of the overall population, but only in 3.0% of those with T2D. This was with a notable gender difference: 5.5% in women and no cases reported in men with T2D. Hyperhidrosis was reported in 2.2% of the participants. Lentigosenilis had a prevalence of 13.4%, while Pityriasis versicolor was observed in 1.9% of the participants. Among those with T2D, the prevalence of Pityriasis versicolor was 5.1%, with a higher prevalence in men (6.0%) compared to women (3.7%) (Table 1).

For peer review only - http://bmjopen.bmj.com/site/about/guidelines.xhtml

228 *Table 1 Descriptive characteristics of the study population*

Variables	Category	Overall	Men	Women	P-value[a]	Type 2 diabetes	healthy	P-value[b]
Sex	Male	3027 (45.5 %)	-	-	-	180 (53.6 %)	3027 (45.5 %)	0.0042
	Female	3633 (54.5 %)	-	-		156 (46.4 %)	3633 (54.5 %)	
BMI (kg/m2)	Underweight	37 (0.6 %)	7 (0.3 %)	30 (0.9 %)	P<0.001	0 (0.0 %)	37 (0.7 %)	P<0.001
	Normalweight	2188 (37.6 %)	735 (28.7 %)	1453 (44.6 %)		25 (8.8 %)	2137 (39.3 %)	
	Overweight	3594 (61.8 %)	1819 (71.0 %)	1775 (54.5 %)		259 (91.2 %)	3262 (60.0 %)	
Diet	Unhealthy	5524 (88.8 %)	2317 (83.4 %)	3207 (93.2 %)	P<0.001	234 (83.0 %)	5208 (89.2 %)	0.002
	Healthy	695 (11.2 %)	461 (16.6 %)	234 (6.8 %)		48 (17.0 %)	630 (10.8 %)	
Sleep quality	Satisfactory	3810 (57.2 %)	1730 (57.3 %)	2080 (57.1 %)	0.962	173 (55.1 %)	3624 (57.3 %)	0.318
	Somewhat unsatisfactory	2295 (34.5 %)	1037 (34.4 %)	1258 (34.5 %)		109 (34.7 %)	2177 (34.4 %)	
	Significantly unsatisfactory	486 (7.3 %)	217 (7.2 %)	269 (7.4 %)		27 (8.6 %)	458 (7.2 %)	
	Totally unsatisfactory	69 (1 %)	33 (1.1 %)	36 (1.0 %)		5 (1.6 %)	62 (1.0 %)	
Anxiety	Mean (sd)	1.3 (0.3)	1.27 (0.28)	1.33 (0.31)	P<0.001	1.36 (0.33)	1.30 (0.30 %)	0.002
Depression	Mean (sd)	1.34 (0.34)	1.29 (0.31)	1.39 (0.36)	P<0.001	1.40 (0.35)	1.34 (0.34 %)	0.004
Education	less than 9 years of comprehensive school	306 (5.5 %)	152 (5.8 %)	154 (5.2 %)	0.169	19 (7.3 %)	280 (5.4 %)	0.003
	Comprehensive school	4362 (78.4 %)	2053 (78.9 %)	2309 (77.9 %)		214 (82.6 %)	4080 (78.1 %)	
	Highly educated	898 (16.1 %)	397 (15.3 %)	501 (16.9 %)		26 (10.0 %)	867 (16.6 %)	
Psoriasis	No	6575 (97.7 %)	2996 (97.6 %)	3579 (97.8 %)	0.702	304 (95.3 %)	6253 (97.8 %)	0.006
	Yes	156 (2.3 %)	74 (2.4 %)	82 (2.2 %)		15 (4.7 %)	140 (2.2 %)	
Pityriasis versicolor	No	1869 (98.1 %)	864 (98.0 %)	1005 (98.1 %)	0.899	94 (94.9 %)	1747 (98.2 %)	0.042
	Yes	37 (1.9 %)	18 (2.0 %)	19 (1.9 %)		5 (5.1 %)	32 (1.8 %)	
Tinea pedis	No	1392 (73 %)	557 (63.2 %)	835 (81.5 %)	P<0.001	55 (55.6 %)	1316 (74.0 %)	P<0.001
	Yes	514 (27 %)	325 (36.8 %)	189 (18.5 %)		44 (44.4 %)	463 (26.0 %)	
Onychomycosis	No	1726 (90.6 %)	757 (85.8 %)	969 (94.6 %)	P<0.001	83 (83.8 %)	1619 (91.0 %)	0.027
	Yes	180 (9.4 %)	125 (14.2 %)	55 (5.4 %)		16 (16.2 %)	160 (9.0 %)	

For peer review only - http://bmjopen.bmj.com/site/about/guidelines.xhtml

Café-au-lait spots	No	1668 (87.5 %)	776 (88.0 %)	892 (87.1 %)	0.614	96 (97.0 %)	1550 (87.1 %)	0.006
	Yes	238 (12.5 %)	106 (12.0 %)	132 (12.9 %)		3 (3.0 %)	229 (12.9 %)	
Lentigo senilis	No	1650 (86.6 %)	794 (90.0 %)	856 (83.6 %)	P<0.001	94 (94.9 %)	1531 (86.1 %)	0.018
	Yes	256 (13.4 %)	88 (10.0 %)	168 (16.4 %)		5 (5.1 %)	248 (13.9 %)	
Rosacea	No	1614 (84.8 %)	790 (89.6 %)	824 (80.6 %)	P<0.001	76 (76.8 %)	1513 (85.1 %)	0.035
	Yes	290 (15.2 %)	92 (10.4 %)	198 (19.4 %)		23 (23.2 %)	264 (14.9 %)	
Hyperhidrosis	No	1864 (97.8 %)	850 (96.4 %)	1014 (99.0 %)	P<0.001	92 (92.9 %)	1744 (98.0 %)	0.003
	Yes	42 (2.2 %)	32 (3.6 %)	10 (1.0 %)		7 (7.1 %)	35 (2.0 %)	
Type 2 diabetes	No	6660 (95.2 %)	3027 (94.4 %)	3633 (95.9 %)	0.004	-	-	-
	Yes	336 (4.8 %)	180 (5.6 %)	156 (4.1 %)		-	-	

P-value[a] for differences between male and female. P-value[b] for differences between people with diabetes and healthy people

For peer review only

For peer review only - http://bmjopen.bmj.com/site/about/guidelines.xhtml

Variable selection

A total of 17 variables were selected using the ridge method, 15 variables with the lasso method, 11 through univariate analysis, 18 with BSLR, 19 with MCP, and 15 with sparse step. The FSLR and SLR methods did not select any variables, and 18 variables were not selected by any of the methods (Fig. 2). Significant variables were identified based on predefined, method-specific criteria: ridge and lasso regression retained variables with nonzero coefficients, univariate analysis defined significance at $p < 0.05$, and BSLR and sparse step methods applied model-specific thresholds optimising the predictive performance.

Overall, excluding the two FALR and SLR methods, the combined agreement among the remaining methods was 0.507—a strong value for six different methods, indicating reliable results. In addition, a pairwise agreement analysis showed that MCP and BSLR, as well as BSLR and Lasso, had the highest agreement values, at 0.96 and 0.86, respectively. The lowest agreement was observed between univariate analysis and Ridge (Supplementary Table S2). A total of 15 variables were initially selected, each identified by a minimum of 4 different selection methods. From these, to ensure proper control over Type I and Type II errors, we filtered results with significant p-values in the univariate analysis. Machine learning models optimise for prediction accuracy, they do not inherently provide statistical significance measures. By applying a p-value threshold, we identified statistically robust associations and reduced the risk of false positives. This approach allowed us to combine the predictive power of machine learning with the inferential strength of traditional statistical methods, ensuring more reliable and interpretable findings. These variables included: Tinea pedis, Psoriasis, Rosacea, Onychomycosis, café-au-lait spots, hyperhidrosis, Lentigo senilis, and pityriasis versicolor. The variable "other skin disease" was eligible for inclusion in the final list of variables based on our selection strategy but was excluded due to its limited informativeness, likely reflecting its broad and non-specific definition during data collection. This highlights the importance of precise variable definitions, as poorly defined variables may reduce their analytical utility.

For peer review only - http://bmjopen.bmj.com/site/about/guidelines.xhtml

The maximum VIF observed was 2.38 (for Anxiety and Depression), and the mean VIF across all variables was 1.21, both well below the commonly used threshold of 10. This indicates that multicollinearity did not pose a concern in the model (Supplementary Table S3)

Path analysis

The results of the path analysis with the standardised path coefficients are depicted in Fig. 3 and Table 2 and the exponentiate of the coefficients is shown in supplementary Table S4. The model fit was evaluated using several indices. The RMSEA (0.060) and SRMR (0.045) were both below 0.08 and the relative Chi-square (CMIN/DF) was calculated as 5.89, which is <6. These findings indicate an acceptable fit of the model to the data. In the model, sex had a direct effect on T2D (OR 1.024, 95%CI 1.001- 1.047), with males having a higher risk compared to females. BMI had a direct effect on T2D (OR 1.043, 95%CI 1.031- 1.055) and an indirect effect, mediated through sleep quality (OR 1, 95 %CI 1- 1.001). The total effect was the sum of direct and indirect effects, at 1.043 with a 95% CI of 1.031-1.056. The total effect was significant at the 0.05 level.

Sleep quality did not show any direct effect on T2D (OR 0.995, 95%CI 0.984-1.006). Diet (OR 1, 95%CI 1- 1.001), anxiety (OR 0.999, 95%CI 0.998- 1.005), depression (OR 1, 95%CI 1- 1.001) and education (OR 1, 95%CI 1- 1) did not show any indirect effect on T2D. The path analysis showed that tinea pedis (OR 1.033 95%CI 1.006-1.059), psoriasis (OR 1.115 95%CI 1.038-1.198), and pityriasis versicolor (OR 1.115 95%CI 1.03-1.208) had positive direct effects and Café-au-lait spots (OR 0.975 95%CI 0.931-0.996) had a negative direct effect on T2D. Finally, we observed that hyperhidrosis had an indirect effect on T2D, mediated through tinea pedis (OR 1.006, 95%CI 1- 1.0019) (Supplementary Table S4).

Table 2 Standardized path coefficients

	Estimate (95% CI)	P-value
**T2D**		

For peer review only - http://bmjopen.bmj.com/site/about/guidelines.xhtml

SEX → T2D	0.024 (0.001, 0.046)	0.038
BMI → T2D	0.042 (0.031, 0.054)	<0.001
Sleep quality → T2D	-0.005 (-0.016, 0.006)	0.398
Psoriasis → T2D	0.109 (0.037, 0.181)	0.003
Pityriasis versicolor → T2D	0.109 (0.03, 0.189)	0.007
Tinea pedis → T2D	0.032 (0.006, 0.057)	0.014
Onychomycosis → T2D	0.033 (-0.005, 0.072)	0.090
Café-au-lait spots → T2D	-0.038 (-0.071, -0.004)	0.026
Lentigo senilis → T2D	-0.025 (-0.057, 0.007)	0.126
Rosacea → T2D	0.011 (-0.021, 0.042)	0.511
**BMI**		
Diet → BMI	0.171 (0.013, 0.329)	0.034
Anxiety → BMI	-0.008 (-0.089, 0.074)	0.856
Depression → BMI	0.033 (-0.046, 0.113)	0.413
**Diet**		
Education → Diet	-0.039 (-0.057, -0.021)	<0.001
**Hyperhidrosis**		
Diet → Hyperhidrosis	0.003 (-0.021, 0.027)	0.812
Anxiety → Hyperhidrosis	0.005 (-0.003, 0.013)	0.251
**Sleep quality**		
Bmi → Sleep quality	0.065 (0.011, 0.118)	0.017
Anxiety → Sleep quality	0.077 (-0.005, 0.159)	0.064
Depression → Sleep quality	0.095 (0.015, 0.175)	0.020
**Tinea pedis**		
Hyperhidrosis → Tinea pedis	0.175 (0.018, 0.332)	0.029
**Chi-square goodness fit test**	324.38	<0.001
**DF**	55	
**CMIN/DF**	5.89	
**RMSEA**	0.060	
**SRMR**	0.045	

Note that: T2D: Type 2 diabetes; BMI: body mass index; SE: Standard Error; RMSEA: root mean square error approximation; SRMR: standardized root mean square residual. Variables listed in the above are standardized variables that are transformed version of an original variable that have been adjusted to have a mean of 0 and a standard deviation of 1

Discussion

To the best of our knowledge, this is the first study to simultaneously investigate multiple skin diseases in relation to type II diabetes and to further incorporate the selected variables into a path analysis. The innovative use of a combination of machine learning techniques and traditional

For peer review only

statistical methods for variable selection has led to more robust and high-quality results, with a promising agreement rate of 0.507 across six different methods. This study identified a significant direct association of pityriasis versicolor, psoriasis, and tinea pedis with T2D. The corresponding odds ratios were 1.115 (95% CI: 1.03, 1.208), 1.115 (95% CI: 1.038, 1.198), and 1.033 (95% CI: 1.006, 1.059), respectively. In contrast, an inverse association was found between café-au-lait spots and T2D, with an odds ratio of 0.963 (95% CI: 0.931, 0.996).

In our study, we found that the prevalence of psoriasis from self-reported information in the population of 46 years was 2.3% and we did not find any difference between men and women. We identified a significant association between T2D and psoriasis, with individuals who have psoriasis being 1.115 times more likely to develop T2D compared to those without psoriasis. This translates to a 0.115 increase in the odds of having T2D for individuals with psoriasis. These findings align with prior research, such as the meta-analysis conducted by Mirghani *et al.,* which reported an OR of 1.38 (95% CI: 1.17-1.64) for the association between psoriasis and diabetes. It is worth noting that while the meta-analysis included both type 1 and type 2 diabetes, our study specifically focused on T2D [21].

It is suggested that diabetes and psoriasis may exacerbate each other, potentially creating a vicious cycle where the presence of one condition aggravates the severity of the other [22][23]. The link between psoriasis and diabetes has been proposed in other reports demonstrating that psoriasis may increase the risk of developing diabetes. People with psoriasis are more likely to develop diabetes compared to those without the condition, and the risk appears to be even higher in severe cases [24][25][26]. Additionally, psoriasis may contribute to insulin resistance, a key factor in the onset of T2D [27]. On the other hand, diabetes, especially when poorly managed, might also increase the likelihood of developing psoriasis. This complex, possibly bidirectional relationship suggests that chronic inflammation and immune system dysfunction are central to the development of both diseases. This may be explained by the shared genes and molecular pathways recently identified to be linked with T2D and psoriasis pathogenesis [28]. This bidirectional relationship highlights the importance of careful management and monitoring of both conditions to prevent worsening outcomes.

For peer review only - http://bmjopen.bmj.com/site/about/guidelines.xhtml

In addition, we examined three fungal diseases: pityriasis versicolor, tinea pedis, and onychomycosis. We observed a direct relationship between diabetes and all three conditions; however, the relationship between onychomycosis and diabetes was not statistically significant in our path model. Our findings regarding fungal infections are consistent with previous research [21][22]. Saud B *et al.*, reported that the prevalence of fungal diseases in diabetic patients is 7.2 times higher compared to individuals without diabetes [29]. Furthermore, participants exhibiting abnormal skin findings in the toe web areas had a 2.5 times increased risk of having undiagnosed diabetes based on OGTT results. The risk was even higher (6 times) when assessed using haemoglobin A1c levels [30]. This highlights the potential role of fungal disease, particularly in the toe web regions, as an indicator for identifying individuals at higher risk of diabetes, which may facilitate earlier detection and intervention.

In our study, individuals with tinea pedis and pityriasis versicolor were found to be 1.03 and 1.115 times more likely, respectively, to present with T2D compared to those without these fungal infections. These results highlight the importance of monitoring and managing fungal infections in patients with diabetes to potentially mitigate the risk and complications associated with T2D.

Furthermore, this study is the first to investigate the relationship between café-au-lait spots and lentigo senilis with T2D. Our findings suggest that individuals with café-au-lait spots and lentigo senilis may have a lower prevalence of T2D compared to those without these skin conditions, although more studies are needed to prove this relationship. Regarding rosacea, we identified a direct association with T2D, although this association was not statistically significant. Interestingly, a study by Stefani EC et al. identified T2D as a risk factor for rosacea [10]. This hypothesis has been addressed by Spoendlin et al., who conducted a population-based case–control analysis using the UK-based General Practice Research Database to analyse the association between rosacea and T2D, reporting a significantly reduced risk of rosacea among individuals with diabetes mellitus (OR = 0.80, 95% CI 0.74–0.85). This inverse association was more pronounced in individuals with higher HbA1c levels and a longer diabetes duration, suggesting that advanced stages of diabetes may be linked to a lower likelihood of developing rosacea [31].

For peer review only - http://bmjopen.bmj.com/site/about/guidelines.xhtml

Moreover, our study identified hyperhidrosis as an indirect risk factor for T2D. This finding aligns with the literature, which indicates that individuals with T2D exhibit impaired thermoregulation compared to healthy individuals, resulting in sweating dysfunction. This impairment may be attributed to several factors, including cardiovascular complications, metabolic imbalances such as irregular insulin distribution and chronic hyperglycaemia, alterations in autonomic nervous function, and reduced blood flow to the skin. Collectively, these factors may influence the sweat gland's function leading to a diminished ability to sweat [32][33][34][35][36].

For peer review only

Limitations

This study has several limitations. In the NFBC1966 study, data on skin conditions were collected by dermatologists at the age of 46 for individuals who were readily accessible and primarily residing in northern Finland. While the involvement of dermatologists enhances the validity of the data, this regional focus, along with the limited sample size, restricts the generalisability of the findings to the broader Finnish population. Additionally, as the data used in this study were collected in 1997 and 2012, the findings may not fully reflect current population characteristics or trends, which could limit their applicability to present-day contexts.

Furthermore, the data were collected at a single time point, so it was impossible to conduct a longitudinal assessment of skin diseases. The relatively small sample size also limited the statistical power and restricted the feasibility of applying more complex models. Consequently, we had to rely on simpler models and relationships to maintain the validity of our findings. Moreover, this was a observational study with a cross-sectional assessment where external factors were not controlled, making it impossible to establish causal relationships. Therefore, it is recommended to use a randomised controlled trial study to investigate causal relationships or employ genetic methods such as Mendelian Randomisation (MR) to explore the causal link between skin diseases and diabetes.

Implications

For peer review only - http://bmjopen.bmj.com/site/about/guidelines.xhtml

These results may have important implications for people's health and quality of life. First, skin diseases are often considered insignificant, and, particularly when accompanied by other illnesses, their treatment is prioritised secondarily. However, people's skin health status is closely linked to their overall health and neglecting it may lead to more serious complications. Second, in people with T2D, skin problems are frequently overlooked until they become severe. Our study has shown that skin diseases are associated with T2D. Based on these findings, we strongly recommend that healthcare providers pay more attention to skin health in people with T2D, ensuring timely diagnosis and treatment to prevent complications and improve people's overall well-being.

Conclusion

In this study, our aim was to investigate the pathway effects of skin diseases on T2D. We employed advanced statistical techniques for variable selection and innovatively combined machine learning approaches with traditional methods, ensuring more robust and reliable findings. The results of a path analysis revealed that tinea pedis, psoriasis, and pityriasis versicolor had a positive effect on T2D, while café-au-lait spots were associated with a negative effect on the condition.

Finally, it is essential to note that skin problems are common in patients with T2D and can be indicative of poor blood sugar control. Early diagnosis and appropriate treatment of these issues can significantly improve patients' quality of life.

Acknowledgments

We wish to thank all cohort members, researchers and NFBC project centre personnel who participated in the NFBC data collections.

Author contributions

MN: Writing - Review & Editing, Conceptualisation, Data curation, Formal analysis, Methodology, Project administration, Software, Validation, Visualisation, Writing - Original Draft,

For peer review only - http://bmjopen.bmj.com/site/about/guidelines.xhtml

Investigation; **LH and SPS:** Data curation and clinical expertise in dermatology, Formal analysis, Review & Editing**; SV:** Review & Editing, funding acquisition; **JH:** Review & Editing, funding acquisition; **NA**: Supervision, Writing - Review & Editing; **SS:** Writing - Review & Editing, Funding acquisition, Conceptualisation, Supervision. All authors approved the final version of the manuscript.

Data Availability Statement

NFBC data are available from the University of Oulu, Infrastructure for Population Studies. Permission to use the data can be applied for research purposes via an electronic material request portal. In the use of data, we follow the EU general data protection regulation (679/2016) and the Finnish Data Protection Act.

The use of personal data is based on a cohort participant's written informed consent in their latest follow-up study, which may pose limitations on its use. Please, contact the NFBC project centre (NFBCprojectcenter(at)oulu.fi) and visit the cohort website (www.oulu.fi/nfbc) for more information.

Funding

This work was supported by The Infotech Oulu project 2022-2025, under the project "Safe biomonitoring by bioICT integrated wearables"; Profi8 Health Dimensions and Business Finland; Horizon 2020 (grant number 874739, LongITools), Horizon Europe (grant number 101057739, TRIGGER; grant number 101137317, IHEN; grant number 101137146, STAGE; grant number 101080250, OBCT; grant number 101080465, OBELISK), the Research Council of Finland (decision number 356888). NFBC1966 received financial support from University of Oulu(grant number. 24000692), Oulu University Hospital (grant number. 24301140), and ERDF European Regional Development Fund (grant number. 539/2010 A31592). The funder didn't influence the results/outcomes of the study despite author affiliations with the funder.

Competing interests

For peer review only

All authors have no competing interests to declare.

References

[1] National Institute of Diabetes and Digestive and Kidney Diseases (NIDDK), *Diabetes in America*, 2nd ed., NIH Pub. No. 95-1468. Bethesda, MD, USA: National Institutes of Health, 1995.

[2] H. Sun *et al.*, "IDF Diabetes Atlas: Global, regional and country-level diabetes prevalence estimates for 2021 and projections for 2045," *Diabetes Res. Clin. Pract.*, vol. 183, p. 109119, 2022.

[3] S. Del Prato, B. Torbeyns, and C. Mathieu, "2024: The year to take European action on diabetes to the next level," *Diabetologia*, vol. 67, no. 7, pp. 1155–1158, 2024, doi: 10.1007/s00125-024-06166-9.

[4] J. Holmes, S. McGill, P. Kind, J. Bottomley, S. Gillam, and M. Murphy, "Health-related quality of life in type 2 diabetes (TARDIS-2)," *Value Health, J. Int. Soc. Pharmacoeconomics Outcomes Res.*, vol. 3, Suppl 1, pp. 47–51, 2000, doi: 10.1046/j.1524-4733.2000.36028.x.

[5] P. Gkogkolou and M. Böhm, "Skin disorders in diabetes mellitus," *JDDG - J. Ger. Soc. Dermatology*, vol. 12, no. 10, pp. 847–864, 2014, doi: 10.1111/ddg.12424.

[6] A. S. Lonberg, L. Skov, A. Skytthe, K. O. Kyvik, O. B. Pedersen, and S. F. Thomsen, "Association of psoriasis with the risk for type 2 diabetes mellitus and obesity," *JAMA Dermatology*, vol. 152, no. 7, pp. 761–767, 2016, doi: 10.1001/jamadermatol.2015.6262.

[7] J. I. Silverberg and P. Greenland, "Eczema and cardiovascular risk factors in 2 US adult population studies," *J. Allergy Clin. Immunol.*, vol. 135, no. 3, pp. 721-728.e6, 2015, doi: 10.1016/j.jaci.2014.11.023.

[8] R. S. Azfar, N. M. Seminara, D. B. Shin, A. B. Troxel, D. J. Margolis, and J. M. Gelfand, "Increased risk of diabetes mellitus and likelihood of receiving diabetes mellitus treatment in patients with psoriasis," *Arch. Dermatol.*, vol. 148, no. 9, pp. 995–1000, Sep. 2012, doi: 10.1001/archdermatol.2012.1401.

For peer review only

For peer review only - http://bmjopen.bmj.com/site/about/guidelines.xhtml

[9] M. I. Perez and S. R. Kohn, “Cutaneous manifestations of diabetes mellitus,” *J. Am. Acad. Dermatol.*, vol. 30, no. 4, pp. 514–519, Apr. 1994, doi: 10.1016/s0190-9622(94)70058-3.

[10] E. C. Stefanadi *et al.*, “Metabolic syndrome and the skin: a more than superficial association. Reviewing the association between skin diseases and metabolic syndrome and a clinical decision algorithm for high risk patients,” *Diabetol. Metab. Syndr.*, vol. 10, p. 9, 2018, doi: 10.1186/s13098-018-0311-z.

[11] University of Oulu, “Northern Finland Birth Cohort 1966,” 1966. http://urn.fi/urn:nbn:fi:att:bc1e5408-980e-4a62-b899-43bec3755243

[12] T. Nordström *et al.*, “Cohort Profile: 46 years of follow-up of the Northern Finland Birth Cohort 1966 (NFBC1966),” *Int. J. Epidemiol.*, vol. 50, no. 6, pp. 1786-1787j, Jan. 2022, doi: 10.1093/ije/dyab109.

[13] S.-P. Sinikumpu *et al.*, “High prevalence of skin diseases and need for treatment in a middle-aged population. A Northern Finland Birth Cohort 1966 study,” *PLoS One*, vol. 9, no. 6, p. e99533, 2014, doi: 10.1371/journal.pone.0099533.

[14] J. Laitinen, K. Pietiläinen, M. Wadsworth, U. Sovio, and M.-R. Järvelin, “Predictors of abdominal obesity among 31-y-old men and women born in Northern Finland in 1966,” *Eur. J. Clin. Nutr.*, vol. 58, no. 1, pp. 180–190, Jan. 2004, doi: 10.1038/sj.ejcn.1601765.

[15] D. Lhewa, S. Banu, B. Rosenfeld, and A. Keller, “Validation of a Tibetan translation of the Hopkins Symptom Checklist 25 and the Harvard Trauma Questionnaire,” *Assessment*, vol. 14, no. 3, pp. 223–230, Sep. 2007, doi: 10.1177/1073191106298876.

[16] T. Hastie, “The elements of statistical learning: data mining, inference, and prediction.” Springer, 2009.

[17] H. L. Kundel and M. Polansky, “Measurement of observer agreement,” *Radiology*, vol. 228, no. 2, pp. 303–308, Aug. 2003, doi: 10.1148/radiol.2282011860.

[18] M. H. Kutner, *Applied Linear Statistical Models*, 2nd ed. New York, NY, USA: McGraw-Hill, 1984.

[19] R. B. Kline, *Principles and practice of structural equation modeling*. Guilford publications,

For peer review only

For peer review only - http://bmjopen.bmj.com/site/about/guidelines.xhtml

2023.

[20] G. J. J. van den Burg, P. J. F. Groenen, and A. Alfons, “SparseStep: Approximating the counting norm for sparse regularization,” pp. 1–15, 2017, [Online]. Available: http://arxiv.org/abs/1701.06967

[21] H. Mirghani *et al.*, “The association of psoriasis, diabetes Mellitus, and hypertension: A meta-analysis,” *Cureus*, vol. 15, no. 11, p. e48855, Nov. 2023, doi: 10.7759/cureus.48855.

[22] R. Abramczyk, J. N. Queller, A. W. Rachfal, and S. S. Schwartz, “Diabetes and psoriasis: Different sides of the same prism,” *Diabetes. Metab. Syndr. Obes.*, vol. 13, pp. 3571–3577, 2020, doi: 10.2147/DMSO.S273147.

[23] A. Vesikansa *et al.*, “Comorbidities and medication use in Finnish patients with psoriasis: A population-based registry study,” *Acta Derm. Venereol.*, vol. 103, p. adv00886, Mar. 2023, doi: 10.2340/actadv.v103.3491.

[24] A. W. Armstrong, C. T. Harskamp, and E. J. Armstrong, “Psoriasis and the risk of diabetes mellitus: a systematic review and meta-analysis,” *JAMA dermatology*, vol. 149, no. 1, pp. 84–91, Jan. 2013, doi: 10.1001/2013.jamadermatol.406.

[25] M. T. Wan, D. B. Shin, R. A. Hubbard, M. H. Noe, N. N. Mehta, and J. M. Gelfand, “Psoriasis and the risk of diabetes: A prospective population-based cohort study.,” *J. Am. Acad. Dermatol.*, vol. 78, no. 2, pp. 315-322.e1, Feb. 2018, doi: 10.1016/j.jaad.2017.10.050.

[26] M. Mamizadeh, Z. Tardeh, and M. Azami, “The association between psoriasis and diabetes mellitus: A systematic review and meta-analysis,” *Diabetes Metab. Syndr.*, vol. 13, no. 2, pp. 1405–1412, 2019, doi: 10.1016/j.dsx.2019.01.009.

[27] A. L. Neimann, D. B. Shin, X. Wang, D. J. Margolis, A. B. Troxel, and J. M. Gelfand, “Prevalence of cardiovascular risk factors in patients with psoriasis,” *J. Am. Acad. Dermatol.*, vol. 55, no. 5, pp. 829–835, Nov. 2006, doi: 10.1016/j.jaad.2006.08.040.

[28] L. Yang, L. Zhang, Q. Du, X. Gong, and J. Tian, “Exploring the molecular mechanism underlying the psoriasis and T2D by using microarray data analysis,” *Sci. Rep.*, vol. 13,

For peer review only

For peer review only - http://bmjopen.bmj.com/site/about/guidelines.xhtml

no. 1, p. 19313, Nov. 2023, doi: 10.1038/s41598-023-46795-5.

[29] B. Saud *et al.*, "Fungal infection among diabetic and nondiabetic individuals in Nepal," *Interdiscip. Perspect. Infect. Dis.*, vol. 2020, p. 7949868, 2020, doi: 10.1155/2020/7949868.

[30] S.-P. Sinikumpu *et al.*, "Abnormal skin in toe webs is a marker for abnormal glucose metabolism. A cross-sectional survey among 1,849 adults in Finland," *Sci. Rep.*, vol. 7, no. 1, p. 9125, Aug. 2017, doi: 10.1038/s41598-017-09354-3.

[31] J. Spoendlin, J. J. Voegel, S. S. Jick, and C. R. Meier, "Risk of rosacea in patients with diabetes using insulin or oral antidiabetic drugs," *J. Invest. Dermatol.*, vol. 133, no. 12, pp. 2790–2793, Dec. 2013, doi: 10.1038/jid.2013.225.

[32] S. Chahal, K. Vohra, and A. Syngle, "Association of sudomotor function with peripheral artery disease in type 2 diabetes," *Neurol. Sci. Off. J. Ital. Neurol. Soc. Ital. Soc. Clin. Neurophysiol.*, vol. 38, no. 1, pp. 151–156, Jan. 2017, doi: 10.1007/s10072-016-2742-3.

[33] W. B. Horton and E. J. Barrett, "Microvascular dysfunction in diabetes mellitus and cardiometabolic disease," *Endocr. Rev.*, vol. 42, no. 1, pp. 29–55, Jan. 2021, doi: 10.1210/endrev/bnaa025.

[34] Y. Liu *et al.*, "Factors influencing sweat gland innervation in diabetes," *Neurology*, vol. 84, no. 16, pp. 1652–1659, Apr. 2015, doi: 10.1212/WNL.0000000000001488.

[35] G. P. Kenny, R. J. Sigal, and R. McGinn, "Body temperature regulation in diabetes," *Temp. (Austin, Tex.)*, vol. 3, no. 1, pp. 119–145, 2016, doi: 10.1080/23328940.2015.1131506.

[36] B. Liu, X. Cheng, M. Chen, W. Dong, J. Sun, and Y. Lun, "Changes of sweat gland function in type 2 diabetes mellitus patients with peripheral neuropathy," *Int. J. Diabetes Dev. Ctries.*, vol. 44, no. 1, pp. 84–90, 2024.

Figure legends

Figure 1 Flow chart demonstrating the process used to select the study participants.

For peer review only

For peer review only - http://bmjopen.bmj.com/site/about/guidelines.xhtml

547 *Figure 2 Results of variable selection methods*

548

549 *Figure 3 The path analysis was performed in 1930 individuals aged 46 years. All depicted arrows represent statistically*
550 *significant associations (Beta coefficients). Model fit values: SRMR = 0.045, RMSEA = 0.060.*

551

552 *Figure S1 Flow chart demonstrating the analytical plan*

553

554

For peer review only

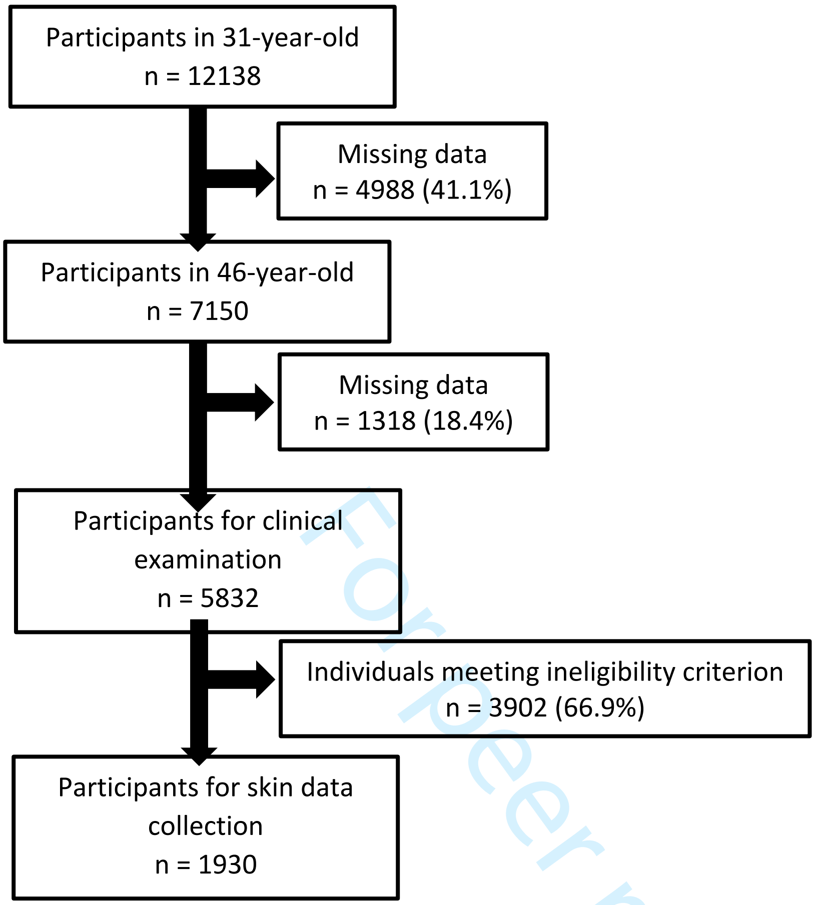

Figure 1 Flow chart demonstrating the process used to select the study participants.

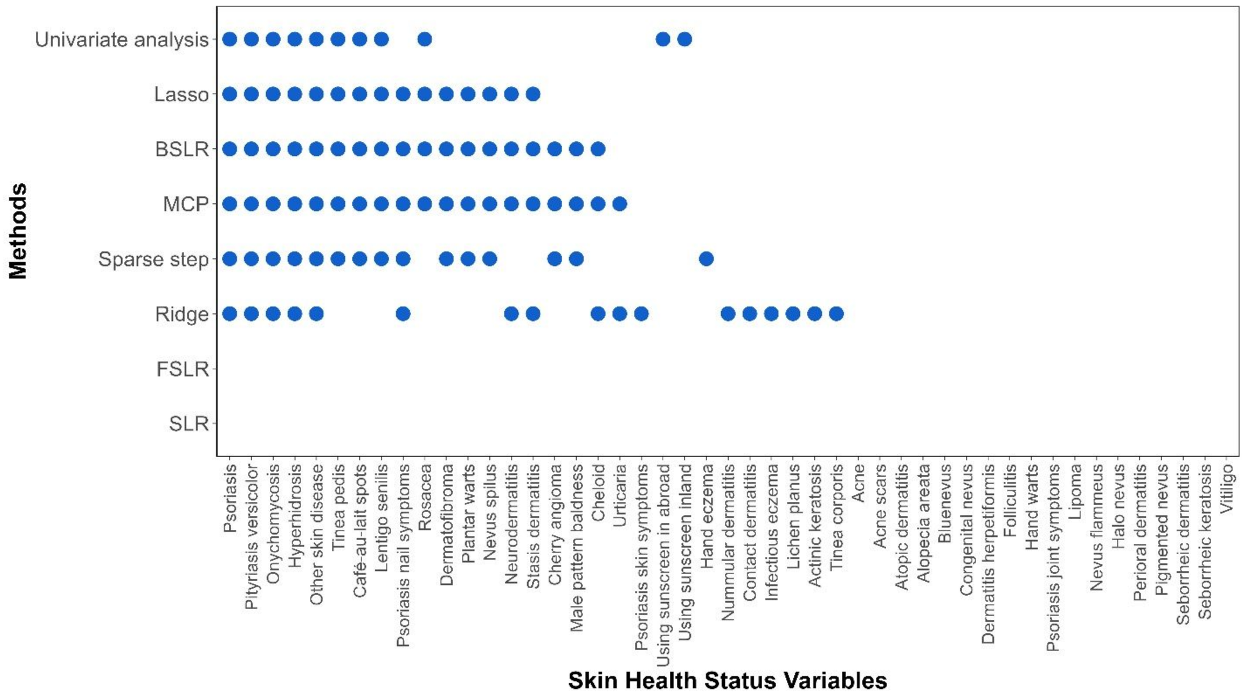

Figure 2 Results of variable selection methods

For peer review only

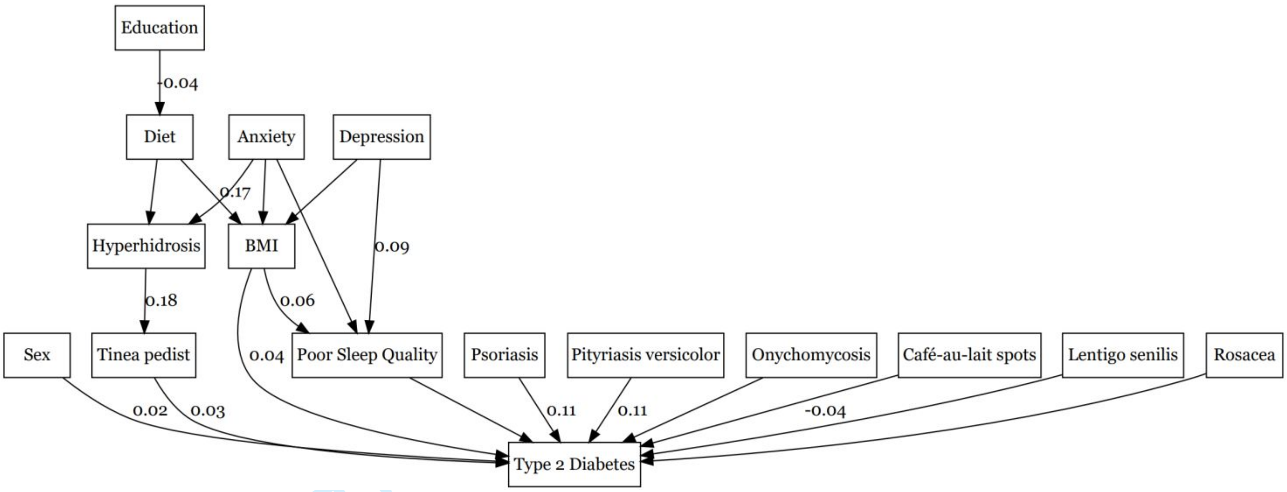

Figure 3 The path analysis was performed in 1930 individuals aged 46 years. All depicted arrows represent statistically significant associations (Beta coefficients). Model fit values: SRMR = 0.045, RMSEA = 0.060.

For peer review only

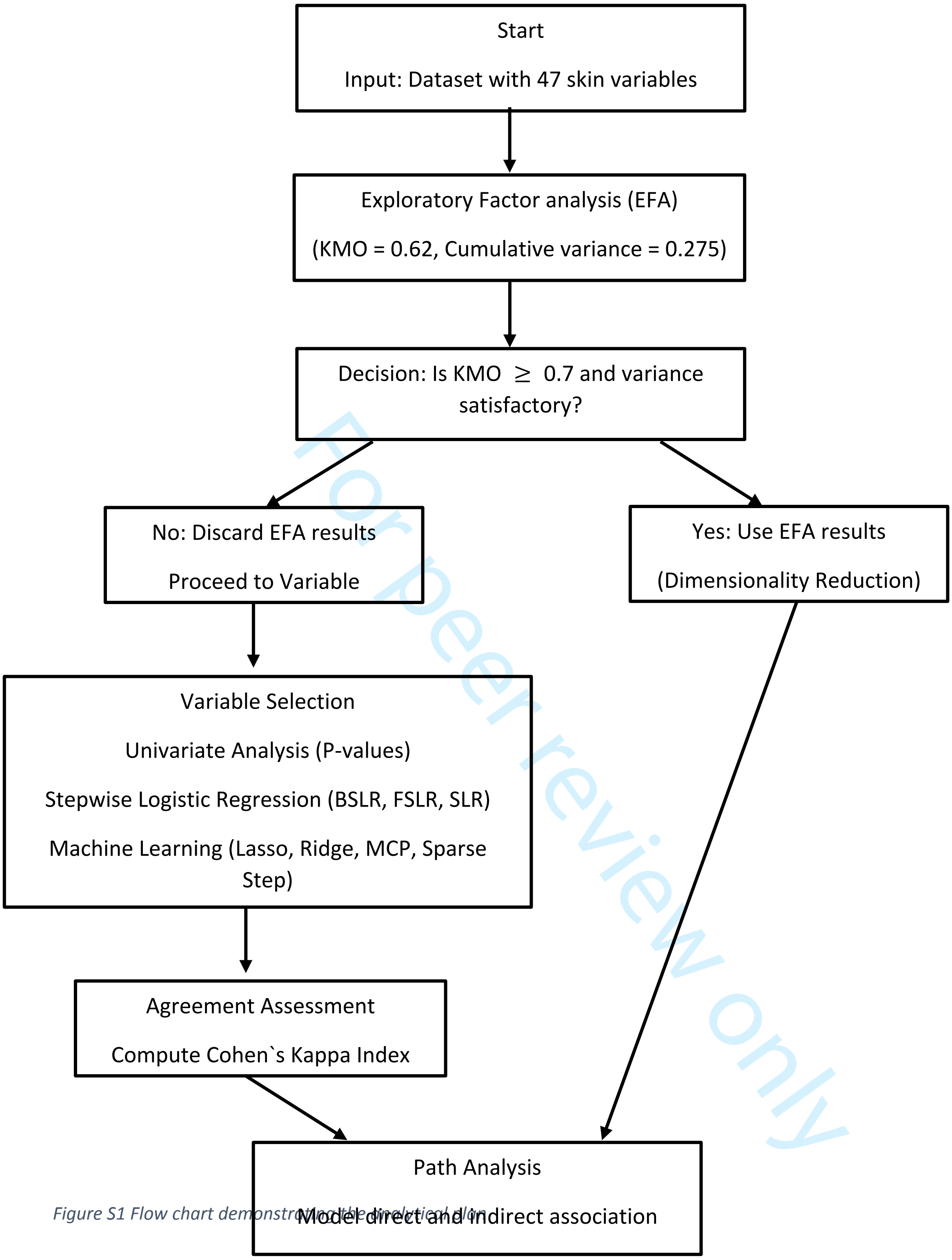

Figure S1 Flow chart demonstrating the analytical plan

For peer review only

Table S1 Descriptive characteristics of the study population

Variable	Category	Overall	Missing	Men	Women	P-value[a]	Type 2 diabetes	healthy	P-value[b]
Tinea pedis	No	1392 (73.0 %)	24 (1.24 %)	557 (63.2 %)	835 (81.5 %)	<0.001	55 (55.6 %)	1316 (74.0 %)	<0.001
	Yes	514 (27.0 %)		325 (36.8 %)	189 (18.5 %)		44 (44.4 %)	463 (26.0 %)	
Hyperhidrosis	No	1864 (97.8 %)	24 (1.24 %)	850 (96.4 %)	1014 (99.0 %)	<0.001	92 (92.9 %)	1744 (98.0 %)	0.003
	Yes	42 (2.2 %)		32 (3.6 %)	10 (1.0 %)		7 (7.1 %)	35 (2.0 %)	
Café-au-lait spots	No	1668 (87.5 %)	24 (1.24 %)	776 (88.0 %)	892 (87.1 %)	0.614	96 (97.0 %)	1550 (87.1 %)	0.006
	Yes	238 (12.5 %)		106 (12.0 %)	132 (12.9 %)		3 (3.0 %)	229 (12.9 %)	
Pigmented nevus	No	1685 (88.4 %)	24 (1.24 %)	766 (86.8 %)	919 (89.7 %)	0.058	88 (88.9 %)	1572 (88.4 %)	1
	Yes	221 (11.6 %)		116 (13.2 %)	105 (10.3 %)		11 (11.1 %)	207 (11.6 %)	
Lentigo senilis	No	1650 (86.6 %)	24 (1.24 %)	794 (90.0 %)	856 (83.6 %)	<0.001	94 (94.9 %)	1531 (86.1 %)	0.018
	Yes	256 (13.4 %)		88 (10.0 %)	168 (16.4 %)		5 (5.1 %)	248 (13.9 %)	
Psoriasis skin symptoms	No	1862 (97.9 %)	28 (1.45 %)	856 (97.4 %)	1006 (98.3 %)	0.198	93 (94.9 %)	1741 (98.0 %)	0.084
	Yes	40 (2.1 %)		23 (2.6 %)	17 (1.7 %)		5 (5.1 %)	35 (2.0 %)	
Psoriasis nail symptoms	No	1889 (99.4 %)	29 (1.5 %)	869 (98.9 %)	1020 (99.8 %)	0.022	98 (100.0 %)	1763 (99.3 %)	0.868
	Yes	12 (0.6 %)		10 (1.1 %)	2 (0.2 %)		0 (0.0 %)	12 (0.7 %)	
Psoriasis joint symptoms	No	1888 (99.3 %)	29 (1.5 %)	874 (99.4 %)	1014 (99.2 %)	0.775	97 (99.0 %)	1763 (99.3 %)	1
	Yes	13 (0.7 %)		5 (0.6 %)	8 (0.8 %)		1 (1.0 %)	12 (0.7 %)	
Psoriasis	No	6575 (97.7 %)	419 (21.71 %)	2996 (97.6 %)	3579 (97.8 %)	0.702	304 (95.3 %)	6253 (97.8 %)	0.006
	Yes	156 (2.3 %)		74 (2.4 %)	82 (2.2 %)		15 (4.7 %)	140 (2.2 %)	
Rosacea	No	1614 (84.8 %)	26 (1.35 %)	790 (89.6 %)	824 (80.6 %)	<0.001	76 (76.8 %)	1513 (85.1 %)	0.035
	Yes	290 (15.2 %)		92 (10.4 %)	198 (19.4 %)		23 (23.2 %)	264 (14.9 %)	
Stasis dermatitis	No	1890 (99.2 %)	25 (1.3 %)	874 (99.1 %)	1016 (99.3 %)	0.773	97 (98.0 %)	1765 (99.3 %)	0.411
	Yes	15 (0.8 %)		8 (0.9 %)	7 (0.7 %)		2 (2.0 %)	13 (0.7 %)	
Bluenevus	No	1880 (98.6 %)	24 (1.24 %)	874 (99.1 %)	1006 (98.2 %)	0.162	97 (98.0 %)	1755 (98.7 %)	0.909
	Yes	26 (1.4 %)		8 (0.9 %)	18 (1.8 %)		2 (2.0 %)	24 (1.3 %)	
Nevus spilus	No	1817 (95.3 %)	24 (1.24 %)	843 (95.6 %)	974 (95.1 %)	0.714	98 (99.0 %)	1691 (95.1 %)	0.121

For peer review only

	Yes	89 (4.7 %)		39 (4.4 %)	50 (4.9 %)		1 (1.0 %)	88 (4.9 %)	
Halo nevus	No	1891 (99.2 %)	24 (1.24 %)	876 (99.3 %)	1015 (99.1 %)	0.819	99 (100.0 %)	1764 (99.2 %)	0.736
	Yes	15 (0.8 %)		6 (0.7 %)	9 (0.9 %)		0 (0.0 %)	15 (0.8 %)	
Dermatofibroma	No	1481 (77.7 %)	24 (1.24 %)	730 (82.8 %)	751 (73.3 %)	<0.001	71 (71.7 %)	1390 (78.1 %)	0.17
	Yes	425 (22.3 %)		152 (17.2 %)	273 (26.7 %)		28 (28.3 %)	389 (21.9 %)	
Cherry angioma	No	759 (39.8 %)	25 (1.3 %)	410 (46.5 %)	349 (34.1 %)	<0.001	44 (44.4 %)	705 (39.7 %)	0.4
	Yes	1146 (60.2 %)		472 (53.5 %)	674 (65.9 %)		55 (55.6 %)	1073 (60.3 %)	
Cheloid	No	1857 (97.4 %)	24 (1.24 %)	859 (97.4 %)	998 (97.5 %)	1	95 (96.0 %)	1735 (97.5 %)	0.526
	Yes	49 (2.6 %)		23 (2.6 %)	26 (2.5 %)		4 (4.0 %)	44 (2.5 %)	
Seborrheic keratosis	No	1061 (55.7 %)	24 (1.24 %)	522 (59.2 %)	539 (52.6 %)	0.005	58 (58.6 %)	987 (55.5 %)	0.616
	Yes	845 (44.3 %)		360 (40.8 %)	485 (47.4 %)		41 (41.4 %)	792 (44.5 %)	
Lipoma	No	1845 (96.8 %)	24 (1.24 %)	841 (95.4 %)	1004 (98.0 %)	0.001	94 (94.9 %)	1726 (97.0 %)	0.389
	Yes	61 (3.2 %)		41 (4.6 %)	20 (2.0 %)		5 (5.1 %)	53 (3.0 %)	
Congenital nevus	No	1828 (95.9 %)	24 (1.24 %)	838 (95.0 %)	990 (96.7 %)	0.086	98 (99.0 %)	1703 (95.7 %)	0.183
	Yes	78 (4.1 %)		44 (5.0 %)	34 (3.3 %)		1 (1.0 %)	76 (4.3 %)	
Nevus flammeus	No	1419 (74.4 %)	24 (1.24 %)	690 (78.2 %)	729 (71.2 %)	0.001	79 (79.8 %)	1316 (74.0 %)	0.241
	Yes	487 (25.6 %)		192 (21.8 %)	295 (28.8 %)		20 (20.2 %)	463 (26.0 %)	
Actinic keratosis	No	1894 (99.4 %)	24 (1.24 %)	875 (99.2 %)	1019 (99.5 %)	0.582	99 (100.0 %)	1767 (99.3 %)	0.864
	Yes	12 (0.6 %)		7 (0.8 %)	5 (0.5 %)		0 (0.0 %)	12 (0.7 %)	
Atopic dermatitis	No	1813 (95.2 %)	25 (1.3 %)	847 (96.0 %)	966 (94.4 %)	0.128	97 (98.0 %)	1689 (95.0 %)	0.269
	Yes	92 (4.8 %)		35 (4.0 %)	57 (5.6 %)		2 (2.0 %)	89 (5.0 %)	
Hand eczema	No	1736 (91.1 %)	25 (1.3 %)	817 (92.6 %)	919 (89.8 %)	0.039	95 (96.0 %)	1614 (90.8 %)	0.115
	Yes	169 (8.9 %)		65 (7.4 %)	104 (10.2 %)		4 (4.0 %)	164 (9.2 %)	
Seborrheic dermatitis	No	1765 (92.7 %)	25 (1.3 %)	774 (87.8 %)	991 (96.9 %)	<0.001	90 (90.9 %)	1650 (92.8 %)	0.613
	Yes	140 (7.3 %)		108 (12.2 %)	32 (3.1 %)		9 (9.1 %)	128 (7.2 %)	
Nummular dermatitis	No	1869 (98.1 %)	25 (1.3 %)	858 (97.3 %)	1011 (98.8 %)	0.021	96 (97.0 %)	1746 (98.2 %)	0.618
	Yes	36 (1.9 %)		24 (2.7 %)	12 (1.2 %)		3 (3.0 %)	32 (1.8 %)	
Infectious eczema	No	1878 (98.6 %)	25 (1.3 %)	862 (97.7 %)	1016 (99.3 %)	0.007	96 (97.0 %)	1754 (98.7 %)	0.351

For peer review only

	Yes	27 (1.4 %)		20 (2.3 %)	7 (0.7 %)		3 (3.0 %)	24 (1.3 %)	
Contact dermatitis	No	1887 (99.1 %)	25 (1.3 %)	880 (99.8 %)	1007 (98.4 %)	0.006	99 (100.0 %)	1761 (99.0 %)	0.665
	Yes	18 (0.9 %)		2 (0.2 %)	16 (1.6 %)		0 (0.0 %)	17 (1.0 %)	
Neurodermatitis	No	1871 (98.2 %)	25 (1.3 %)	857 (97.2 %)	1014 (99.1 %)	0.002	95 (96.0 %)	1748 (98.3 %)	0.186
	Yes	34 (1.8 %)		25 (2.8 %)	9 (0.9 %)		4 (4.0 %)	30 (1.7 %)	
Lichen planus	No	1893 (99.3 %)	24 (1.24 %)	874 (99.1 %)	1019 (99.5 %)	0.407	99 (100.0 %)	1766 (99.3 %)	0.817
	Yes	13 (0.7 %)		8 (0.9 %)	5 (0.5 %)		0 (0.0 %)	13 (0.7 %)	
Dermatitis herpetiformis	No	1900 (99.7 %)	24 (1.24 %)	880 (99.8 %)	1020 (99.6 %)	0.821	99 (100.0 %)	1773 (99.7 %)	1
	Yes	6 (0.3 %)		2 (0.2 %)	4 (0.4 %)		0 (0.0 %)	6 (0.3 %)	
Vitiligo	No	1874 (98.3 %)	24 (1.24 %)	872 (98.9 %)	1002 (97.9 %)	0.123	98 (99.0 %)	1748 (98.3 %)	0.881
	Yes	32 (1.7 %)		10 (1.1 %)	22 (2.1 %)		1 (1.0 %)	31 (1.7 %)	
Urticaria	No	1896 (99.5 %)	24 (1.24 %)	877 (99.4 %)	1019 (99.5 %)	1	98 (99.0 %)	1770 (99.5 %)	1
	Yes	10 (0.5 %)		5 (0.6 %)	5 (0.5 %)		1 (1.0 %)	9 (0.5 %)	
Acne	No	1755 (92.1 %)	25 (1.3 %)	818 (92.7 %)	937 (91.6 %)	0.399	94 (94.9 %)	1635 (92.0 %)	0.377
	Yes	150 (7.9 %)		64 (7.3 %)	86 (8.4 %)		5 (5.1 %)	143 (8.0 %)	
Perioral dermatitis	No	1888 (99.3 %)	28 (1.45 %)	881 (99.9 %)	1007 (98.7 %)	0.007	98 (99.0 %)	1762 (99.3 %)	1
	Yes	14 (0.7 %)		1 (0.1 %)	13 (1.3 %)		1 (1.0 %)	13 (0.7 %)	
Acne scars	No	1716 (90.2 %)	28 (1.45 %)	780 (88.5 %)	936 (91.7 %)	0.026	89 (90.8 %)	1602 (90.2 %)	0.981
	Yes	186 (9.8 %)		101 (11.5 %)	85 (8.3 %)		9 (9.2 %)	174 (9.8 %)	
Pityriasis versicolor	No	1869 (98.1 %)	24 (1.24 %)	864 (98.0 %)	1005 (98.1 %)	0.9	94 (94.9 %)	1747 (98.2 %)	0.058
	Yes	37 (1.9 %)		18 (2.0 %)	19 (1.9 %)		5 (5.1 %)	32 (1.8 %)	
Onychomycosis	No	1726 (90.6 %)	24 (1.24 %)	757 (85.8 %)	969 (94.6 %)	<0.001	83 (83.8 %)	1619 (91.0 %)	0.027
	Yes	180 (9.4 %)		125 (14.2 %)	55 (5.4 %)		16 (16.2 %)	160 (9.0 %)	
Tinea corporis	No	1898 (99.6 %)	24 (1.24 %)	878 (99.5 %)	1020 (99.6 %)	1	98 (99.0 %)	1772 (99.6 %)	0.901
	Yes	8 (0.4 %)		4 (0.5 %)	4 (0.4 %)		1 (1.0 %)	7 (0.4 %)	
Folliculitis	No	1790 (93.9 %)	24 (1.24 %)	796 (90.2 %)	994 (97.1 %)	<0.001	95 (96.0 %)	1667 (93.7 %)	0.488
	Yes	116 (6.1 %)		86 (9.8 %)	30 (2.9 %)		4 (4.0 %)	112 (6.3 %)	

Plantar warts	No	1732 (90.9 %)	24 (1.24 %)	809 (91.7 %)	923 (90.1 %)	0.263	94 (94.9 %)	1613 (90.7 %)	0.207
	Yes	174 (9.1 %)		73 (8.3 %)	101 (9.9 %)		5 (5.1 %)	166 (9.3 %)	
Hand warts	No	1855 (97.3 %)	24 (1.24 %)	860 (97.5 %)	995 (97.2 %)	0.754	96 (97.0 %)	1731 (97.3 %)	1
	Yes	51 (2.7 %)		22 (2.5 %)	29 (2.8 %)		3 (3.0 %)	48 (2.7 %)	
Alopecia areata	No	1899 (99.6 %)	24 (1.24 %)	877 (99.4 %)	1022 (99.8 %)	0.338	99 (100.0 %)	1773 (99.7 %)	1
	Yes	7 (0.4 %)		5 (0.6 %)	2 (0.2 %)		0 (0.0 %)	6 (0.3 %)	
Male pattern baldness	No	1291 (67.7 %)	24 (1.24 %)	280 (31.7 %)	1011 (98.7 %)	<0.001	69 (69.7 %)	1202 (67.6 %)	0.741
	Yes	615 (32.3 %)		602 (68.3 %)	13 (1.3 %)		30 (30.3 %)	577 (32.4 %)	
Other skin disease	No	1595 (90.2 %)	162 (8.39 %)	753 (91.2 %)	842 (89.4 %)	0.453	74 (82.2 %)	1497 (90.6 %)	0.021
	Yes	168 (88.4 %)		71 (8.6 %)	97 (10.3 %)		16 (17.8 %)	150 (9.1 %)	
	Perhaps	5 (11.6 %)		2 (0.2 %)	3 (0.3 %)		0 (0.0 %)	5 (0.3 %)	
Using sunscreen inland	Not at all	1068 (15.9 %)	423 (21.92 %)	759 (24.7 %)	309 (8.5 %)	<0.001	75 (23.2 %)	987 (15.5 %)	<0.001
	Sometimes	3170 (47.1 %)		1600 (52.1 %)	1570 (43.0 %)		150 (46.4 %)	3004 (47.1 %)	
	Reqularly	2210 (32.9 %)		624 (20.3 %)	1586 (43.4 %)		78 (24.1 %)	2128 (33.4 %)	
	I do not spend time in the sun	279 (4.1 %)		89 (2.9 %)	190 (5.2 %)		20 (6.2 %)	259 (4.1 %)	
Using sunscreen in abroad	Not at all	960 (15.1 %)	805 (41.71 %)	640 (22.1 %)	320 (9.3 %)	<0.001	71 (23.1 %)	885 (14.7 %)	<0.001
	Sometimes	574 (9.0 %)		367 (12.7 %)	207 (6.0 %)		28 (9.1 %)	542 (9.0 %)	
	Reqularly	4079 (64.3 %)		1620 (55.9 %)	2459 (71.4 %)		166 (54.1 %)	3900 (64.8 %)	
	I do not spend time in the sun	732 (11.5 %)		272 (9.4 %)	460 (13.3 %)		42 (13.7 %)	690 (11.5 %)	

P-value[a] for differences between male and female. P-value[b] for differences between people with diabetes and healthy.

For peer review only

Table S2 Agreement coefficient between different methods

Method 1	Method 2	Cohen kappa (95% CI)	P_value
Ridge	Lasso	0.24 (-0.043, 0.53)	0.094
Ridge	Univariate analysis	0.16 (-0.122, 0.448)	0.248
Ridge	BSLR	0.23 (-0.058, 0.511)	0.12
Ridge	MCP	0.28 (0.002, 0.56)	0.053
Ridge	Sparse step	0.05 (-0.233, 0.342)	0.708
Lasso	Univariate analysis	0.53 (0.27, 0.799)	<0.001
Lasso	BSLR	0.86 (0.709, 1)	<0.001
Lasso	MCP	0.82 (0.649, 0.986)	<0.001
Lasso	Sparse step	0.71 (0.488, 0.924)	<0.001
Univariate analysis	BSLR	0.42 (0.159, 0.687)	0.002
Univariate analysis	MCP	0.39 (0.128, 0.651)	0.005
Univariate analysis	Sparse step	0.43 (0.151, 0.711)	0.003
BSLR	MCP	0.96 (0.869, 1)	<0.001
BSLR	Sparse step	0.77 (0.577, 0.958)	<0.001
MCP	Sparse step	0.73 (0.524, 0.927)	<0.001

For peer review only

Table S3 Variance inflation factor for selected variables

Variable	VIF
BMI	1.08
Diet	1.04
Poor Quality of sleep	1.06
Anxiety	2.38
Depression	2.38
Education	1.06
Psoriasis	1.03
Pityriasis versicolor	1.02
Tinea pedis	1.16
Onychomycosis	1.12
Café_au_lait_spots	1.01
Lentigo senilis	1.01
Rosacea	1.04
Hyperhidrosis	1.05

For peer review only

Table S4 Exponentiated Path coefficient analysis

Independent Variable	Total Effect	Direct Effect	Indirect Effect
Sex	1.024 (1.001, 1.047)	1.024 (1.001, 1.047)	-
BMI	1.043 (1.031, 1.056)	1.043 (1.031, 1.055)	1 (1, 1.001)
Diet	1 (1, 1.001)	-	1 (1, 1.001)
Poor Quality of sleep	0.995 (0.984, 1.006)	0.995 (0.984, 1.006)	-
Anxiety	0.999 (0.997, 1.005)	-	0.999 (0.997, 1.005)
Depression	1 (1, 1.001)	-	1 (1, 1.001)
Education	1 (1, 1)	-	1 (1, 1)
Psoriasis	1.115 (1.038, 1.198)	1.115 (1.038, 1.198)	-
Pityriasis versicolor	1.115 (1.03, 1.208)	1.115 (1.03, 1.208)	-
Tinea pedis	1.033 (1.006, 1.059)	1.033 (1.006, 1.059)	-
Onychomycosis	1.034 (0.995, 1.075)	1.034 (0.995, 1.075)	-
Café-au-lait spots	0.963 (0.931, 0.996)	0.963 (0.931, 0.996)	-
Lentigo senilis	0.975 (0.945, 1.007)	0.975 (0.945, 1.007)	-
Rosacea	1.011 (0.979, 1.043)	1.011 (0.979, 1.043)	-
Hyperhidrosis	1.006 (1, 1.019)	-	1.006 (1, 1.019)

For peer review only

BMJ Open

Assessing the association of Type 2 diabetes with skin health status: A study of the Northern Finland Birth Cohort 1966

Journal:	*BMJ Open*
Manuscript ID	bmjopen-2025-109709.R2
Article Type:	Original research
Date Submitted by the Author:	24-Jun-2026
Complete List of Authors:	Nasserinejad, Maryam; University of Oulu, Research Unit of Population Health, Faculty of Medicine<br>Huilaja, Laura; University of Oulu, Research Unit of Clinical Medicine, Medical Research Center<br>Sinikumpu, Suvi-Päivikki; University of Oulu, Research Unit of Clinical Medicine, Medical Research Center<br>Vainio, Seppo ; University of Oulu, Faculty of Biochemistry and Molecular Medicine, Disease Networks, Laboratory of Developmental Biology<br>Röning, Juha ; University of Oulu, Biomimetics and Intelligent Systems Group, Faculty of Information Technology and Electrical Engineering<br>Ali, Nsrein ; University of Oulu, Faculty of Biochemistry and Molecular Medicine, Disease Networks, Laboratory of Developmental Biology; University of Oulu, Research Unit of Health Sciences and Technology. Faculty of Medicine<br>Sebert, Sylvain; University of Oulu, Research Unit of Population Health, Faculty of Medicine
**Primary Subject Heading**:	Diabetes and endocrinology
Secondary Subject Heading:	Dermatology
Keywords:	Dermatology < INTERNAL MEDICINE, Public health < INFECTIOUS DISEASES, General diabetes < DIABETES & ENDOCRINOLOGY

SCHOLARONE
MANUSCRIPTS

I, the Submitting Author has the right to grant and does grant on behalf of all authors of the Work (as defined in the below author licence), an exclusive licence and/or a non-exclusive licence for contributions from authors who are: i) UK Crown employees; ii) where BMJ has agreed a CC-BY licence shall apply, and/or iii) in accordance with the terms applicable for US Federal Government officers or employees acting as part of their official duties; on a worldwide, perpetual, irrevocable, royalty-free basis to BMJ Publishing Group Ltd ("BMJ") its licensees and where the relevant Journal is co-owned by BMJ to the co-owners of the Journal, to publish the Work in this journal and any other BMJ products and to exploit all rights, as set out in our licence.

The Submitting Author accepts and understands that any supply made under these terms is made by BMJ to the Submitting Author unless you are acting as an employee on behalf of your employer or a postgraduate student of an affiliated institution which is paying any applicable article publishing charge ("APC") for Open Access articles. Where the Submitting Author wishes to make the Work available on an Open Access basis (and intends to pay the relevant APC), the terms of reuse of such Open Access shall be governed by a Creative Commons licence – details of these licences and which Creative Commons licence will apply to this Work are set out in our licence referred to above.

Other than as permitted in any relevant BMJ Author's Self Archiving Policies, I confirm this Work has not been accepted for publication elsewhere, is not being considered for publication elsewhere and does not duplicate material already published. I confirm all authors consent to publication of this Work and authorise the granting of this licence.

For peer review only

Assessing the association of Type 2 diabetes with skin health status: A study of the Northern Finland Birth Cohort 1966

Maryam Nasserinejad [1]✉ Laura Huilaja [2], Suvi-Päivikki Sinikumpu [2], Seppo Vainio [3], Juha Röning [4,5], Nsrein Ali [2,3,6], Sylvain Sebert [1]

[1] Research Unit of Population Health, Faculty of Medicine, University of Oulu, Oulu, Finland. [2] Research Unit of Clinical Medicine, Medical Research Center, University of Oulu and Department of Dermatology, Oulu University Hospital, Oulu, Finland. [3] Faculty of Biochemistry and Molecular Medicine, Disease Networks, Laboratory of Developmental Biology, University of Oulu, Oulu, Finland. [4] Biomimetics and Intelligent Systems Group, Faculty of Information Technology and Electrical Engineering, University of Oulu, 90014 Oulu, Finland. [5] Symbiosis Institute of Technology, Symbiosis International (Deemed University) India. [6] Research Unit of Health Sciences and Technology. Faculty of Medicine, 90220Oulu. Oulu Finland. ✉ email: maryam.nasserinejad@oulu.fi

For peer review only - http://bmjopen.bmj.com/site/about/guidelines.xhtml

Abstract

Objectives: There is evidence to suggest that alterations in skin health often co-occur with type 2 diabetes (T2D). However, the underlying mechanisms remain unclear. Despite their clinical relevance, skin conditions are often underdiagnosed in diabetes care. This study applied variable selection methods to identify key dermatological factors linked to T2D and examined their direct and indirect associations.

Design: Prospective birth cohort study.

Participants: This study analysed data from the Northern Finland Birth Cohort 1966 (NFBC1966), including 1,906 participants (1,024 women, 882 men) at ages 31y and 46y. At age 46y the cohort participants, living in the region of Northern Finland, were invited to a general health clinical examination including the assessment of their T2D status and a dermatological examination.

Methods: Prior to performing a path analysis, we developed a data driven procedure to select 47 possible skin-related variables into the model. The variable selection combined test-based methods (i.e. a univariate analysis, and a backward, forward, and stepwise logistic regression) and penalty-based/machine learning methods (i.e. Lasso, Ridge, minimax convex penalty, and Sparse Stepwise Regression). The selected skin-related variables were then included in a path analyses accounting for sex, body mass index (BMI), diet, education, depression, anxiety and sleep quality.

Results: Eight skin-related variables were selected and included in the path analysis. Psoriasis (Odds Raito (OR) 1.105, 95% CI 1.058–1.156) and Tinea pedis (OR 1.067, 95% CI 1.04–1.096) showed direct associations with T2D. Hyperhidrosis (OR 1.017, 95% CI 1.005–1.037) exhibited an indirect effect.

Conclusion: These results underscored the interconnectedness of dermatological conditions such as psoriasis and Tinea pedis with T2D. These findings suggest that certain skin conditions

For peer review only

may serve as potential indicators of T2D. Early recognition and appropriate management of these conditions may help improve patients' quality of life.

Strengths and limitations of this study:

- Dermatological examinations were diagnosed by dermatologists, enhancing the accuracy and validity of the data.
- Use of path analyses to examine direct and indirect associations while adjusting for key confounders.
- Applied multiple statistical and machine learning methods for variable selection to improve reliability.
- A community-based study with limited sample size may introduce selection factors and restrict generalisability to the broader Finnish population.
- Data were collected at a single time point, preventing a longitudinal assessment and causal inference.

For peer review only

For peer review only - http://bmjopen.bmj.com/site/about/guidelines.xhtml

Introduction

Type 2 diabetes (T2D) is one main segments of chronic non-communicable diseases characterised by elevated levels of blood glucose and is associated with serious complications to the heart, blood vessels, digestive system, eyes, kidneys, nerves, skin, and even psychosocial aspects and early mortality [1].

An estimated 537 million adults aged 20–79 years worldwide (10.5% of all adults in this age group) had diabetes in 2021. By 2030, this number is projected to rise to 643 millions, and by 2045, it could reach 783 millions. This would represent a 46% increase in the number of people living with diabetes, while the global population is estimated to grow by only 20% over the same period [2]. According to the World Health Organisation, in the European Region alone, approximately 64 millions of adults and around 300,000 children and adolescents are living with diabetes as of 2024, making it one of the most common chronic conditions in Europe [3]. According to the latest reports, among the European Nordic countries, Finland has the highest prevalence of diabetes, at 9.7%. The number of adults with diabetes is estimated to be 392.9 thousand (95% CI: 334.8,435.9) [2].

The burden of and the premature mortality attributed to T2D are very high, and most people living with T2D are reported to suffer other complications leading to a dramatic reduction in their quality of life [4]. The comorbidity of skin disease and T2D is undeniable, and almost one third of people with diabetes have skin lesions [5].

Numerous studies have shown associations between different types of skin disorders with T2D. For example, substantial evidence supports a link between inflammatory diseases, such as psoriasis and eczema, and T2D [6][7]. In addition, Azfar et *al.* proposed previous incidence of psoriasis as a risk factor for the development of T2D [8]. Other skin conditions associated with T2D, such as acanthosis nigricans, diabetic dermopathy, necrobiosis lipoidica, bacterial and fungal infections, and xerosis (dry skin), often appear early, sometimes before T2D is diagnosed. These skin disorders may not only reflect the systemic effects of diabetes but can also serve as

For peer review only - http://bmjopen.bmj.com/site/about/guidelines.xhtml

early indicators, emphasising the importance of timely recognition and management to improve the prevention and/or reduce the burden of T2D [9][10].

It is currently hypothesised that a large number of skin-related conditions may be directly associated with T2D either through causal mechanisms or psycho-social pathways. This study was designed to identify the most relevant dermatological conditions associated with T2D in the general population living in Northern Finland to examine possible direct and indirect relationships between skin health and T2D, while adjusting for potential confounders and covariates such diet, education, BMI, anxiety, depression, sleep quality, and sex.

Materials and methods

Overview and data resources

The selected population

The current study was derived from a longitudinal birth cohort study and used data from the participants of the Northern Finland Birth Cohort 1966 (NFBC1966) [11]. The NFBC1966 is a longitudinal population-based study initiated to investigate factors affecting health and development from prenatal stages into adulthood. The cohort comprised 12,138 live births from 1966, representing 96.3% of all births that year in Finland's two northernmost provinces, Oulu and Lapland [12]. The selection process to define the selected study participants from the eligible population is shown in the study flowchart (Fig. 1). The study included all eligible participants with available follow-up data on their skin health status and T2D defined at age 46 years (in 2012) and all available covariates. Due to logistic and financial constraints, the clinical examination for dermatology diagnosis was only performed for participants living in the region of Oulu and was conducted at the University Hospital of Oulu. It included a total of 882 males and 1,024 females (N = 1,906). To handle missing data, multiple imputations were performed which implements multiple imputation by chained equations (MICE). This approach imputes missing values iteratively by specifying a regression model for each variable with missing data, conditional on the other variables in the dataset. The number of missing values for each variable is presented

For peer review only - http://bmjopen.bmj.com/site/about/guidelines.xhtml

in Supplementary Table S1. The studies received ethical approval from Northern Ostrobothnia Hospital District Ethical Committee 94/2011 (12.12.2011).

Patient and public involvement

Neither patient groups nor participants in the study were involved in the design, conduct, reporting, or dissemination plans of this research.

Exposure variables: Skin health status variables

Each individual skin health status could be defined from 46 different variables (Supplementary Table S1). These variables, also referred to as measures, were part of a clinical protocol for a comprehensive dermatological assessment, designed by dermatologist, to study the prevalence of major skin diseases and disorders in mid-life at age 46 [13]. All variables were collected through clinical examinations, except for doctor-diagnosed psoriasis, which was obtained via a postal questionnaire, and two variables related to sunscreen use, which were self-reported by the participants.

Outcome variable: T2D

All T2D-related data were obtained from the 46-year follow-up of the NFBC1966 cohort. We defined cases with T2D according to the following criteria depending on their level of participation or prior status for T2D. All eligible participants were invited to an oral glucose tolerance test (OGTT) with a 75g anhydrous glucose ingestion. From this test, we classified individuals with T2D (N cases = 267) if the fasting OGTT value was 7 mmol/l or higher, and the value at two hours-post OGTT was 11.1 mmol/l or greater. Additionally, cases with T2D (N cases = 192) were included according to self-reports with the following question: “Do you currently have or have you ever had adult-onset diabetes (type 2)?” Finaly, we included additional cases should they report the use of blood sugar-lowering medications like metformin (N cases = 102). Some participants met more than one of the above criteria, resulting in overlap between the groups. The total number of unique individuals with T2D was 336.

For peer review only - http://bmjopen.bmj.com/site/about/guidelines.xhtml

This comprehensive methodology ensured a robust identification process for individuals with T2D, incorporating both clinical measurements and self-reported information.

Covariate variables

To account for potential confounding, the model included covariates measured at two different follow-ups. Education, anxiety, depression, and diet were used to capture long-term influences from the 31-year follow-up. In contrast, BMI and sleep quality were included as more proximal and time-sensitive factors from the 46-year follow-up to better reflect their temporal proximity to the outcome.

Diet was categorised as a binary variable (healthy vs. unhealthy) based on consumption patterns of vegetables, fruits, rye, and processed foods [14]. Education was defined by a combination of two different questions concerning basic education and vocational qualifications with three levels from basic educated to highly educated.

For defining anxiety and depression, we followed the Hopkins symptom checklist [15]. This questionnaire consists of 14 questions aimed at diagnosing anxiety, focusing on symptoms such as pain, tension, restlessness, trembling, fear, and heart pounding. Additionally, there are 13 questions designed to assess depression, covering issues such as difficulty falling asleep, feelings of loneliness and worthlessness, sexual difficulties, lack of energy, and suicidal thoughts. The questions were presented in a four-point Likert format, with one being 'not at all' and four being 'very much'. The depression and anxiety scores were derived from the mean scores, which ranged slightly from 1 to 4, where 1 indicated no anxiety or depression and 4 indicated a high level of anxiety or depression.

Information on sleep quality was collected by questionnaire at age 46 years.

To assess potential selection bias arising from participant attrition between the two follow-ups, we compared the baseline covariate distributions at the 31-year follow-up between participants

For peer review only

who attended the 46-year follow-up and those who did not; the results are presented in Supplementary Table S2.

Statistical methods

Descriptive statistics, including counts and percentages for all variables, were computed overall and stratified by sex (male and female) and T2D status (healthy and T2D groups). Chi-square tests were used to compare categorical variables between sex groups and between health status groups as presented. In addition, covariates were compared over time, and McNemar's test and paired t-tests were used (Supplementary Table S3). Initially, we applied an exploratory factor analysis (EFA) to reduce the dimensions of the skin status variables, aiming to derive components from the pool of 47 skin health status. However, due to the Kaiser–Meyer–Olkin (KMO) index of 0.62 and a low cumulative variance (0.275 out of 1.00) for just the first 22 components, we opted against utilising the outcomes of this model. We opted for an alternative approach to reducing the dimensionality through variable selection.

Variable selection

Variable selection is a crucial step in inferential modelling, as accurately identifying the subset of variables truly associated with the outcome of interest is essential. General approaches to variable selection fall into three main categories: test-based, penalty-based, and screening-based methods [16]. While various techniques for variable selection exist, each has distinct mathematical properties, which can lead to differing outcomes in terms of the variables selected. This raises an important challenge for researchers: determining how the choice of selection method influences the study results and their reproducibility across different methods. Investigating the “between-method” variability in variable selection is, therefore, an important area of study.

Best practices suggest employing multiple methods and assigning confidence to variables based on their consistency across methods. In this study, we employed a variety of methods to identify the most appropriate variables for path analysis modelling, including a univariate logistic

For peer review only

For peer review only - http://bmjopen.bmj.com/site/about/guidelines.xhtml

regression analysis based on p-values, as well as a backward stepwise logistic regression (BSLR), forward stepwise logistic regression (FSLR), and stepwise logistic regression (SLR). Additionally, we utilised machine learning techniques, specifically least absolute shrinkage and selection operator regression (Lasso), Ridge regression, minimax convex penalty (MCP), and Sparse Stepwise Regression. The degree of agreement between the results of the various methods was assessed using Cohen's Kappa index, which ranges from -1 to 1. Values less than 0 indicate poor agreement (worse than chance), A Kappa value below 0 indicates poor agreement (worse than chance), while values between 0.61 and 0.80 suggest substantial agreement, and values above 0.80 indicate almost perfect agreement [17]. The multicollinearity of the selected variables was assessed using the variance inflation factor (VIF), with a maximum VIF exceeding 10 indicating potential influence of multicollinearity on the least squares estimates [18].

Path analysis

Following the selection of skin health status, we employed a path analysis to examine both direct and indirect associations, while controlling for the effects of sex, body mass index (BMI), diet, education, depression, and anxiety (Supplementary Fig. S1).

In this study, variables were standardised to facilitate comparison and to eliminate variation arising from differences in scale. The standardisation transformed the original variables so they would have a mean of 0 and a standard deviation of 1, making comparisons between variables with different scales or units more straightforward and for model estimation, the weighted least squares mean and variance adjusted (WLSMV) method was used.

The model fit was evaluated using the root mean square error of approximation (RMSEA), the standardised root mean square residual (SRMR), and the relative chi-square (Chi-square minimum discrepancy (CMIN)/Degrees of Freedom (DF)). Values of RMSEA and SRMR below 0.08 and CMIN/DF below 5 were considered indicative of an acceptable model fit, based on established recommendations in the structural equation modelling literature [19].

For peer review only

For peer review only - http://bmjopen.bmj.com/site/about/guidelines.xhtml

All analyses were conducted using R software, version 4.5.2. specifically the glmnet package for Lasso and Ridge regression, ncvreg package for MCP method, sparsestep [20] for sparse step method and the lavaan package for path analysis.

Results

Among the study participants, the overall prevalence of T2D was 4.8%, with a significantly higher rate observed in men (5.6%) compared to women (4.1%). The prevalence of tinea pedis was 27%, with a higher prevalence in men than in women. Specifically, among patients with T2D, the prevalence of tinea pedis increased to 44.4%, with 55.5% in men and 35.2% in women (Table 1). Psoriasis was reported by 2.5% of the participants overall, with a higher prevalence in patients with T2D at 7.1%. In contrast with tinea pedis, this prevalence was higher in women (7.4%) compared to men (6.8%). Rosacea affected 15.2% of the participants, and among those with T2D, the prevalence rose to 23.2%, showing a significant gender disparity with 8.9% in men and 35.2% in women (Table 1). Onychomycosis was observed in 9.4% of the participants. Café-au-lait spots were found in 12.5% of the overall population, but only in 3.0% of those with T2D. This was with a notable gender difference: 5.6% in women and no cases reported in men with T2D. Hyperhidrosis was reported in 2.2% of the participants. Lentigo senilis had a prevalence of 13.4%, while Pityriasis versicolor was observed in 1.9% of the participants. Among those with T2D, the prevalence of Pityriasis versicolor was 5.1%, with a higher prevalence in men (6.7%) compared to women (3.7%) (Table 1).

For peer review only

Table 1 Descriptive characteristics of the study population

Variables	Category	Overall	Men	Women	Healthy	Type 2 diabetes	Type 2 diabetes	
							Men	Women
Sex	Male	3027 (45.5 %)	-	-	3027 (45.5 %)	180 (53.6 %) *	-	-
	Female	3633 (54.5 %)	-	-	3633 (54.5 %)	156 (46.4 %)	-	-
BMI (kg/m2)	Underweight	37 (0.6 %)	7 (0.3 %)	30 (0.9 %) *	37 (0.7 %)	0 (0.0 %) *	0 (0.0 %)	0 (0.0 %)
	Normalweight	2188 (37.6 %)	735 (28.7 %)	1453 (44.6 %)	2137 (39.3 %)	25 (8.8 %)	8 (32 %)	17 (68 %)
	Overweight	3594 (61.8 %)	1819 (71.0 %)	1775 (54.5 %)	3262 (60.0 %)	259 (91.2 %)	148 (57 %)	111 (0.0 %)
Diet	Unhealthy	5524 (88.8 %)	2317 (83.4 %)	3207 (93.2 %) *	5208 (89.2 %)	234 (83.0 %) *	104 (72.2 %)	130 (94.2 %)
	Healthy	695 (11.2 %)	461 (16.6 %)	234 (6.8 %)	630 (10.8 %)	48 (17.0 %)	40 (27.8 %)	8 (5.8 %)
Sleep quality	Satisfactory	3810 (57.2 %)	1730 (57.3 %)	2080 (57.1 %)	3624 (57.3 %)	173 (55.1 %)	87 (52.7 %)	86 (57.7 %)
	Somewhat unsatisfactory	2295 (34.5 %)	1037 (34.4 %)	1258 (34.5 %)	2177 (34.4 %)	109 (34.7 %)	60 (36.4 %)	49 (32.9 %)
	Significantly unsatisfactory	486 (7.3 %)	217 (7.2 %)	269 (7.4 %)	458 (7.2 %)	27 (8.6 %)	16 (9.7 %)	11 (7.4 %)
	Totally unsatisfactory	69 (1 %)	33 (1.1 %)	36 (1.0 %)	62 (1.0 %)	5 (1.6 %)	2 (1.2 %)	3 (2.0 %)
Anxiety	Mean (sd)	1.3 (0.3)	1.27 (0.28)	1.33 (0.31) *	1.30 (0.30 %)	1.36 (0.33) *	1.36 (0.33)	1.36 (0.34)
Depression	Mean (sd)	1.34 (0.34)	1.29 (0.31)	1.39 (0.36) *	1.34 (0.34 %)	1.40 (0.35) *	1.38 (0.35)	1.43 (0.35)
Education	less than 9 years of comprehensive school	306 (5.5 %)	152 (5.8 %)	154 (5.2 %)	280 (5.4 %)	19 (7.3 %) *	8 (5.6 %)	11 (9.4 %)
	Comprehensive school	4362 (78.4 %)	2053 (78.9 %)	2309 (77.9 %)	4080 (78.1 %)	214 (82.6 %)	121 (85.2 %)	93 (79.5 %)
	High educated	898 (16.1 %)	397 (15.3 %)	501 (16.9 %)	867 (16.6 %)	26 (10.0 %)	13 (9.2 %)	13 (11.1 %)
Psoriasis	No	1783 (97.5 %)	820 (97.4 %)	963 (97.7 %)	1686 (97.8 %)	91 (92.9 %) *	41 (93.2 %)	50 (92.6 %)
	Yes	45 (2.5 %)	22 (2.6 %)	23 (2.3 %)	38 (2.2 %)	7 (7.1 %)	3 (6.8 %)	4 (7.4 %)
Pityriasis versicolor	No	1869 (98.1 %)	864 (98.0 %)	1005 (98.1 %)	1747 (98.2 %)	94 (94.9 %) *	42 (93.3 %)	52 (96.3 %)

For peer review only - http://bmjopen.bmj.com/site/about/guidelines.xhtml

For peer review only

	Yes	37 (1.9 %)	18 (2.0 %)	19 (1.9 %)	32 (1.8 %)	5 (5.1 %)	3 (6.7 %)	2 (3.7 %)
Tinea pedis	No	1392 (73 %)	557 (63.2 %)	835 (81.5 %) *	1316 (74.0 %)	55 (55.6 %) *	20 (44.4 %)	35 (64.8 %)
	Yes	514 (27 %)	325 (36.8 %)	189 (18.5 %)	463 (26.0 %)	44 (44.4 %)	25 (55.6 %)	19 (35.2 %)
Onychomycosis	No	1726 (90.6 %)	757 (85.8 %)	969 (94.6 %) *	1619 (91.0 %)	83 (83.8 %) *	34 (75.6 %)	49 (90.7 %)
	Yes	180 (9.4 %)	125 (14.2 %)	55 (5.4 %)	160 (9.0 %)	16 (16.2 %)	11 (24.4 %)	5 (9.3 %)
Café-au-lait spots	No	1668 (87.5 %)	776 (88.0 %)	892 (87.1 %) *	1550 (87.1 %)	96 (97.0 %) *	45 (100.0 %)	51 (94.4 %)
	Yes	238 (12.5 %)	106 (12.0 %)	132 (12.9 %)	229 (12.9 %)	3 (3.0 %)	0 (0.0 %)	3 (5.6 %)
Lentigo senilis	No	1650 (86.6 %)	794 (90.0 %)	856 (83.6 %) *	1531 (86.1 %)	94 (94.9 %) *	45 (100.0 %)	49 (90.7 %)
	Yes	256 (13.4 %)	88 (10.0 %)	168 (16.4 %)	248 (13.9 %)	5 (5.1 %)	0 (0.0 %)	5 (9.3 %)
Rosacea	No	1614 (84.8 %)	790 (89.6 %)	824 (80.6 %) *	1513 (85.1 %)	76 (76.8 %) *	41 (91.1 %)	35 (64.8 %)
	Yes	290 (15.2 %)	92 (10.4 %)	198 (19.4 %)	264 (14.9 %)	23 (23.2 %)	4 (8.9 %)	19 (35.2 %)
Hyperhidrosis	No	1864 (97.8 %)	850 (96.4 %)	1014 (99.0 %) *	1744 (98.0 %)	92 (92.9 %) *	42 (93.3 %)	50 (92.6 %)
	Yes	42 (2.2 %)	32 (3.6 %)	10 (1.0 %)	35 (2.0 %)	7 (7.1 %)	3 (6.7 %)	4 (7.4 %)
Type 2 diabetes	No	6660 (95.2 %)	3027 (94.4 %)	3633 (95.9 %) *	-	-	-	-
	Yes	336 (4.8 %)	180 (5.6 %)	156 (4.1 %)	-	-	180 (53.6 %)	156 (46.4 %)

An asterisk (*) indicates statistical significance at the 0.05 level for differences between male and female and differences between people with diabetes and healthy people

For peer review only - http://bmjopen.bmj.com/site/about/guidelines.xhtml

Variable selection

A total of 17 variables were selected using the ridge method, 15 variables with the lasso method, 11 through univariate analysis, 18 with BSLR, 19 with MCP, and 15 with sparse step. The FSLR and SLR methods did not select any variables, and 18 variables were not selected by any of the methods (Fig. 2). Significant variables were identified based on predefined, method-specific criteria: ridge and lasso regression retained variables with nonzero coefficients, univariate analysis defined significance at $p < 0.05$, and BSLR and sparse step methods applied model-specific thresholds optimising the predictive performance.

Overall, excluding the two FALR and SLR methods, the combined agreement among the remaining methods was 0.507, a strong value for six different methods, indicating reliable results. In addition, a pairwise agreement analysis showed that MCP and BSLR, as well as BSLR and Lasso, had the highest agreement values, at 0.96 and 0.86, respectively. The lowest agreement was observed between univariate analysis and Ridge (Supplementary Table S4). A total of 15 variables were initially selected, each identified by a minimum of 4 different selection methods. From these, to ensure proper control over Type I and Type II errors, we filtered results with significant p-values in the univariate analysis. Machine learning models optimise for prediction accuracy, they do not inherently provide statistical significance measures. By applying a p-value threshold, we identified statistically robust associations and reduced the risk of false positives. This approach allowed us to combine the predictive power of machine learning with the inferential strength of traditional statistical methods, ensuring more reliable and interpretable findings. These variables included: Tinea pedis, Psoriasis, Rosacea, Onychomycosis, café-au-lait spots, hyperhidrosis, Lentigo senilis, and pityriasis versicolor. The variable “other skin disease” was eligible for inclusion in the final list of variables based on our selection strategy but was excluded due to its limited informativeness, likely reflecting its broad and non-specific definition during data collection. This highlights the importance of precise variable definitions, as poorly defined variables may reduce their analytical utility.

For peer review only - http://bmjopen.bmj.com/site/about/guidelines.xhtml

The maximum VIF observed was 2.38 (for Anxiety and Depression), and the mean VIF across all variables was 1.21, both well below the commonly used threshold of 10. This indicates that multicollinearity did not pose a concern in the model (Supplementary Table S5)

Path analysis

The results of the path analysis with the standardised path coefficients are depicted in Fig. 3 and Table 2 and the exponentiate of the coefficients is shown in supplementary Table S6. The model fit was evaluated using several indices. The RMSEA (0.040) and SRMR (0.031) were both below 0.08 and the relative Chi-square (CMIN/DF) was calculated as 4.12, which is < 5. These findings indicate an acceptable fit of the model to the data. In the model, sex was not significantly associated with T2D (OR 1.004, 95%CI 0.982–1.026), indicating no evidence of a meaningful difference in risk between males and females. BMI had a direct effect on T2D (OR 1.011, 95%CI 1.008- 1.014) and an indirect effect, mediated through sleep quality (OR 1.00, 95 %CI 1.00-1.001). The total effect was the sum of direct and indirect effects, at 1.011 with a 95% CI of 1.008-1.014. The total effect was significant at the 0.05 level.

Sleep quality did not show any direct effect on T2D (OR 0.995, 95%CI 0.984-1.005). Diet (OR 1, 95%CI 1- 1.001), anxiety (OR 0.999, 95%CI 0.992- 1.018), depression (OR 0.998, 95%CI 0.998-1.003) and education (OR 1, 95%CI 1-1) did not show any indirect effect on T2D. The path analysis showed that tinea pedis (OR 1.067 95%CI 1.04-1.096), psoriasis (OR 1.105 95%CI 1.058-1.156), rosacea (OR 1.03 95%CI 1.004-1.058), onychomycosis (OR 1.042 95%CI 1.01-1.074), and pityriasis versicolor (OR 1.091 95%CI 1.038-1.147) had positive direct effects on T2D. Finally, we observed that hyperhidrosis had a direct effect on T2D, mediated through tinea pedis (OR 1.017, 95%CI 1.005- 1.037) (Supplementary Table S6).

Table 2 Standardized path coefficients

	Estimate (95% CI)	P-value
**T2D**		

For peer review only - http://bmjopen.bmj.com/site/about/guidelines.xhtml

SEX → T2D	0.004 (-0.018, 0.026)	0.746
BMI → T2D	0.011 (0.008, 0.014)	<0.001
Sleep quality → T2D	-0.005 (-0.016, 0.005)	0.332
Psoriasis → T2D	0.1 (0.056, 0.145)	<0.001
Pityriasis versicolor → T2D	0.087 (0.037, 0.137)	0.001
Tinea pedis → T2D	0.065 (0.039, 0.092)	<0.001
Onychomycosis → T2D	0.041 (0.01, 0.071)	0.008
Café-au-lait spots → T2D	-0.043 (-0.101, 0.015)	0.142
Lentigo senilis → T2D	-0.035 (-0.08, 0.011)	0.136
Rosacea → T2D	0.03 (0.004, 0.056)	0.023
**BMI**		
Diet → BMI	0.949 (0.386, 1.512)	0.001
Anxiety → BMI	0.012 (-0.91, 0.934)	0.979
Depression → BMI	0.383 (-0.403, 1.168)	0.34
**Diet**		
Education → Diet	-0.097 (-0.135, -0.06)	<0.001
**Hyperhidrosis**		
Diet → Hyperhidrosis	-0.005 (-0.023, 0.014)	0.615
Anxiety → Hyperhidrosis	0.018 (-0.023, 0.059)	0.382
**Sleep quality**		
Bmi → Sleep quality	0.017 (0.004, 0.03)	0.011
Anxiety → Sleep quality	0.285 (0.022, 0.549)	0.034
Depression → Sleep quality	0.351 (0.113, 0.589)	0.004
**Tinea pedis**		
Hyperhidrosis → Tinea pedis	0.262 (0.131, 0.392)	<0.001
**Chi-square goodness fit test**	226.773	<0.001
**DF**	55	
**CMIN/DF**	4.12	
**RMSEA**	0.04	
**SRMR**	0.031	

293 Note that: T2D: Type 2 diabetes; BMI: body mass index; SE: Standard Error; RMSEA: root mean square
294 error approximation; SRMR: standardized root mean square residual. Variables listed in the above are
295 standardized variables that are transformed version of an original variable that have been adjusted to
296 have a mean of 0 and a standard deviation of 1

297

298

299 Discussion

300 To the best of our knowledge, this is the first study to simultaneously investigate multiple skin
301 diseases in relation to type II diabetes and to further incorporate the selected variables into a
302 path analysis. The innovative use of a combination of machine learning techniques and traditional

For peer review only - http://bmjopen.bmj.com/site/about/guidelines.xhtml

statistical methods for variable selection has led to more robust and high-quality results, with a promising agreement rate of 0.507 across six different methods. This study identified a significant direct association of psoriasis, pityriasis versicolor, tinea pedis, onychomycosis and rosacea with T2D. The corresponding odds ratios were 1.105 (95% CI: 1.058, 1.156), 1.091 (95% CI: 1.038, 1.147), 1.067 (95% CI:1.04, 1.096), 1.042 (95% CI: 1.01, 1.074), and 1.03 (95% CI:1.004, 1.058), respectively.

In our study, we found that the prevalence of psoriasis from self-reported information in the population of 46 years was 2.5% and we did not find any difference between men and women. We identified a significant association between T2D and psoriasis, with individuals who have psoriasis being 1.105 times more likely to develop T2D compared to those without psoriasis. This translates to a 0.105 increase in the odds of having T2D for individuals with psoriasis. These findings align with prior research, such as the meta-analysis conducted by Mirghani *et al.,* which reported an OR of 1.38 (95% CI: 1.17-1.64) for the association between psoriasis and diabetes. It is worth noting that while the meta-analysis included both type 1 and type 2 diabetes, our study specifically focused on T2D [21].

It is suggested that diabetes and psoriasis may exacerbate each other, potentially creating a vicious cycle where the presence of one condition aggravates the severity of the other [22][23]. The link between psoriasis and diabetes has been proposed in other reports demonstrating that psoriasis may increase the risk of developing diabetes. People with psoriasis are more likely to develop diabetes compared to those without the condition, and the risk appears to be even higher in severe cases [24][25][26]. Additionally, psoriasis may contribute to insulin resistance, a key factor in the onset of T2D [27]. On the other hand, diabetes, especially when poorly managed, might also increase the likelihood of developing psoriasis. This complex, possibly bidirectional relationship suggests that chronic inflammation and immune system dysfunction are central to the development of both diseases. This may be explained by the shared genes and molecular pathways recently identified to be linked with T2D and psoriasis pathogenesis [28]. This bidirectional relationship highlights the importance of careful management and monitoring of both conditions to prevent worsening outcomes.

For peer review only - http://bmjopen.bmj.com/site/about/guidelines.xhtml

331 In addition, we examined three fungal diseases: pityriasis versicolor, tinea pedis, and onychomycosis. We observed a direct relationship between diabetes and all three conditions, Our findings regarding fungal infections are consistent with previous research [21][22]. Saud B *et al.*, reported that the prevalence of fungal diseases in diabetic patients is 7.2 times higher compared to individuals without diabetes [29]. Furthermore, participants exhibiting abnormal skin findings in the toe web areas had a 2.5 times increased risk of having undiagnosed diabetes based on OGTT results. The risk was even higher (6 times) when assessed using haemoglobin A1c levels [30]. This highlights the potential role of fungal disease, particularly in the toe web regions, as an indicator for identifying individuals at higher risk of diabetes, which may facilitate earlier detection and intervention.

In our study, individuals with pityriasis versicolor, tinea pedis, and onychomycosis were found to be 1.09, 1.07 and 1.04 times more likely, respectively, to present with T2D compared to those without these fungal infections. These results highlight the importance of monitoring and managing fungal infections in patients with diabetes to potentially mitigate the risk and complications associated with T2D.

Furthermore, this study is the first to investigate the relationship between café-au-lait spots and lentigo senilis with T2D. However, we did not identify a statistically significant association between either of these skin conditions and T2D. Further studies are warranted to confirm these findings and to better understand any potential relationship. Regarding rosacea, we identified a direct association with T2D, Individuals with rosacea were found to be 1.03 times more likely to present with T2D compared to those without this skin condition. Interestingly, a study by Stefani EC et al. identified T2D as a risk factor for rosacea [10]. This hypothesis has been addressed by Spoendlin et al., who conducted a population-based case–control analysis using the UK-based General Practice Research Database to analyse the association between rosacea and T2D, reporting a significantly reduced risk of rosacea among individuals with diabetes mellitus (OR = 0.80, 95% CI 0.74–0.85). This inverse association was more pronounced in individuals with higher HbA1c levels and a longer diabetes duration, suggesting that advanced stages of diabetes may be linked to a lower likelihood of developing rosacea [31].

For peer review only - http://bmjopen.bmj.com/site/about/guidelines.xhtml

Moreover, our study identified hyperhidrosis as an indirect risk factor for T2D. This finding aligns with the literature, which indicates that individuals with T2D exhibit impaired thermoregulation compared to healthy individuals, resulting in sweating dysfunction. This impairment may be attributed to several factors, including cardiovascular complications, metabolic imbalances such as irregular insulin distribution and chronic hyperglycaemia, alterations in autonomic nervous function, and reduced blood flow to the skin. Collectively, these factors may influence the sweat gland's function leading to a diminished ability to sweat [32][33][34][35][36].

Limitations

This study has several limitations. In the NFBC1966 study, data on skin conditions were collected by dermatologists at the age of 46 for individuals who were readily accessible and primarily residing in northern Finland. While the involvement of dermatologists enhances the validity of the data, this regional focus, along with the limited sample size, restricts the generalisability of the findings to the broader Finnish population. Additionally, as the data used in this study were collected in 1997 and 2012, the findings may not fully reflect current population characteristics or trends, which could limit their applicability to present-day contexts. Furthermore, the definition of type 2 diabetes was based on the OGTT results, self-reports, and antidiabetic medication use, which may introduce potential misclassification.

In addition, attrition and loss to follow-up are common in longitudinal studies. In the NFBC1966 cohort, differences in characteristics were observed between participants who participated in the 46-year follow-up and those who did not. These differences suggest the possibility of differential attrition, which may introduce selection bias and should be considered when interpreting the findings.

Furthermore, the data were collected at a single time point, so it was impossible to conduct a longitudinal assessment of skin diseases. The relatively small sample size also limited the statistical power and restricted the feasibility of applying more complex models. Consequently, we had to rely on simpler models and relationships to maintain the validity of our findings. Moreover, this was a observational study with a cross-sectional assessment where external

For peer review only - http://bmjopen.bmj.com/site/about/guidelines.xhtml

factors were not controlled, making it impossible to establish causal relationships. Therefore, it is recommended to use a randomised controlled trial study to investigate causal relationships or employ genetic methods such as Mendelian Randomisation (MR) to explore the causal link between skin diseases and diabetes.

Implications

These results may have important implications for people's health and quality of life. First, skin diseases are often considered insignificant, and, particularly when accompanied by other illnesses, their treatment is prioritised secondarily. However, people's skin health status is closely linked to their overall health and neglecting it may lead to more serious complications. Second, in people with T2D, skin problems are frequently overlooked until they become severe. Our study has shown that skin diseases are associated with T2D. Based on these findings, we strongly recommend that healthcare providers pay more attention to skin health in people with T2D, ensuring timely diagnosis and treatment to prevent complications and improve people's overall well-being.

Conclusion

In this study, our aim was to investigate the pathway effects of skin diseases on T2D. We employed advanced statistical techniques for variable selection and innovatively combined machine learning approaches with traditional methods, ensuring more robust and reliable findings. The results of a path analysis revealed that tinea pedis, psoriasis, and pityriasis versicolor had a positive effect on T2D, while café-au-lait spots were associated with a negative effect on the condition.

Finally, it is essential to note that skin problems are common in patients with T2D and can be indicative of poor blood sugar control. Early diagnosis and appropriate treatment of these issues can significantly improve patients' quality of life.

Acknowledgments

For peer review only

For peer review only - http://bmjopen.bmj.com/site/about/guidelines.xhtml

We wish to thank all cohort members, researchers and NFBC project centre personnel who participated in the NFBC data collections.

Author contributions

MN: Writing - Review & Editing, Conceptualisation, Data curation, Formal analysis, Methodology, Project administration, Software, Validation, Visualisation, Writing - Original Draft, Investigation; Maryam Nasserinejad is the guarantor of the study. **LH and SPS:** Data curation and clinical expertise in dermatology, Formal analysis, Review & Editing**; SV:** Review & Editing, funding acquisition; **JH:** Review & Editing, funding acquisition; **NA**: Supervision, Writing - Review & Editing; **SS:** Writing - Review & Editing, Funding acquisition, Conceptualisation, Supervision. All authors approved the final version of the manuscript.

Data Availability Statement

NFBC data are available from the University of Oulu, Infrastructure for Population Studies. Permission to use the data can be applied for research purposes via an electronic material request portal. In the use of data, we follow the EU general data protection regulation (679/2016) and the Finnish Data Protection Act.

The use of personal data is based on a cohort participant's written informed consent in their latest follow-up study, which may pose limitations on its use. Please, contact the NFBC project centre (NFBCprojectcenter(at)oulu.fi) and visit the cohort website (www.oulu.fi/nfbc) for more information.

Funding

This work was supported by The Infotech Oulu project 2022-2025, under the project "Safe biomonitoring by bioICT integrated wearables"; Profi8 Health Dimensions and Business Finland; Horizon 2020 (grant number 874739, LongITools), Horizon Europe (grant number 101057739, TRIGGER; grant number 101137317, IHEN; grant number 101137146, STAGE; grant number 101080250, OBCT; grant number 101080465, OBELISK), the Research Council

For peer review only - http://bmjopen.bmj.com/site/about/guidelines.xhtml

For peer review only

of Finland (decision number 356888). NFBC1966 received financial support from University of Oulu (grant number. 24000692), Oulu University Hospital (grant number. 24301140), and ERDF European Regional Development Fund (grant number. 539/2010 A31592). The funder didn't influence the results/outcomes of the study despite author affiliations with the funder.

Competing interests

All authors have no competing interests to declare.

References

[1] National Institute of Diabetes and Digestive and Kidney Diseases (NIDDK), *Diabetes in America*, 2nd ed., NIH Pub. No. 95-1468. Bethesda, MD, USA: National Institutes of Health, 1995.

[2] H. Sun *et al.*, "IDF Diabetes Atlas: Global, regional and country-level diabetes prevalence estimates for 2021 and projections for 2045," *Diabetes Res. Clin. Pract.*, vol. 183, p. 109119, 2022.

[3] S. Del Prato, B. Torbeyns, and C. Mathieu, "2024: The year to take European action on diabetes to the next level," *Diabetologia*, vol. 67, no. 7, pp. 1155–1158, 2024, doi: 10.1007/s00125-024-06166-9.

[4] J. Holmes, S. McGill, P. Kind, J. Bottomley, S. Gillam, and M. Murphy, "Health-related quality of life in type 2 diabetes (TARDIS-2)," *Value Health, J. Int. Soc. Pharmacoeconomics Outcomes Res.*, vol. 3, Suppl 1, pp. 47–51, 2000, doi: 10.1046/j.1524-4733.2000.36028.x.

[5] P. Gkogkolou and M. Böhm, "Skin disorders in diabetes mellitus," *JDDG - J. Ger. Soc. Dermatology*, vol. 12, no. 10, pp. 847–864, 2014, doi: 10.1111/ddg.12424.

[6] A. S. Lonberg, L. Skov, A. Skytthe, K. O. Kyvik, O. B. Pedersen, and S. F. Thomsen, "Association of psoriasis with the risk for type 2 diabetes mellitus and obesity," *JAMA Dermatology*, vol. 152, no. 7, pp. 761–767, 2016, doi: 10.1001/jamadermatol.2015.6262.

[7] J. I. Silverberg and P. Greenland, "Eczema and cardiovascular risk factors in 2 US adult

For peer review only - http://bmjopen.bmj.com/site/about/guidelines.xhtml

population studies," *J. Allergy Clin. Immunol.*, vol. 135, no. 3, pp. 721-728.e6, 2015, doi: 10.1016/j.jaci.2014.11.023.

[8] R. S. Azfar, N. M. Seminara, D. B. Shin, A. B. Troxel, D. J. Margolis, and J. M. Gelfand, "Increased risk of diabetes mellitus and likelihood of receiving diabetes mellitus treatment in patients with psoriasis," *Arch. Dermatol.*, vol. 148, no. 9, pp. 995–1000, Sep. 2012, doi: 10.1001/archdermatol.2012.1401.

[9] M. I. Perez and S. R. Kohn, "Cutaneous manifestations of diabetes mellitus," *J. Am. Acad. Dermatol.*, vol. 30, no. 4, pp. 514–519, Apr. 1994, doi: 10.1016/s0190-9622(94)70058-3.

[10] E. C. Stefanadi *et al.*, "Metabolic syndrome and the skin: a more than superficial association. Reviewing the association between skin diseases and metabolic syndrome and a clinical decision algorithm for high risk patients," *Diabetol. Metab. Syndr.*, vol. 10, p. 9, 2018, doi: 10.1186/s13098-018-0311-z.

[11] University of Oulu, "Northern Finland Birth Cohort 1966," 1966. http://urn.fi/urn:nbn:fi:att:bc1e5408-980e-4a62-b899-43bec3755243

[12] T. Nordström *et al.*, "Cohort Profile: 46 years of follow-up of the Northern Finland Birth Cohort 1966 (NFBC1966)," *Int. J. Epidemiol.*, vol. 50, no. 6, pp. 1786-1787j, Jan. 2022, doi: 10.1093/ije/dyab109.

[13] S.-P. Sinikumpu *et al.*, "High prevalence of skin diseases and need for treatment in a middle-aged population. A Northern Finland Birth Cohort 1966 study," *PLoS One*, vol. 9, no. 6, p. e99533, 2014, doi: 10.1371/journal.pone.0099533.

[14] J. Laitinen, K. Pietiläinen, M. Wadsworth, U. Sovio, and M.-R. Järvelin, "Predictors of abdominal obesity among 31-y-old men and women born in Northern Finland in 1966," *Eur. J. Clin. Nutr.*, vol. 58, no. 1, pp. 180–190, Jan. 2004, doi: 10.1038/sj.ejcn.1601765.

[15] D. Lhewa, S. Banu, B. Rosenfeld, and A. Keller, "Validation of a Tibetan translation of the Hopkins Symptom Checklist 25 and the Harvard Trauma Questionnaire," *Assessment*, vol. 14, no. 3, pp. 223–230, Sep. 2007, doi: 10.1177/1073191106298876.

[16] T. Hastie, "The elements of statistical learning: data mining, inference, and prediction."

For peer review only

For peer review only - http://bmjopen.bmj.com/site/about/guidelines.xhtml

Springer, 2009.

[17] H. L. Kundel and M. Polansky, “Measurement of observer agreement,” *Radiology*, vol. 228, no. 2, pp. 303–308, Aug. 2003, doi: 10.1148/radiol.2282011860.

[18] M. H. Kutner, *Applied Linear Statistical Models*, 2nd ed. New York, NY, USA: McGraw-Hill, 1984.

[19] R. B. Kline, *Principles and practice of structural equation modeling*. Guilford publications, 2023.

[20] G. J. J. van den Burg, P. J. F. Groenen, and A. Alfons, “SparseStep: Approximating the counting norm for sparse regularization,” pp. 1–15, 2017, [Online]. Available: http://arxiv.org/abs/1701.06967

[21] H. Mirghani *et al.*, “The association of psoriasis, diabetes Mellitus, and hypertension: A meta-analysis,” *Cureus*, vol. 15, no. 11, p. e48855, Nov. 2023, doi: 10.7759/cureus.48855.

[22] R. Abramczyk, J. N. Queller, A. W. Rachfal, and S. S. Schwartz, “Diabetes and psoriasis: Different sides of the same prism,” *Diabetes. Metab. Syndr. Obes.*, vol. 13, pp. 3571–3577, 2020, doi: 10.2147/DMSO.S273147.

[23] A. Vesikansa *et al.*, “Comorbidities and medication use in Finnish patients with psoriasis: A population-based registry study,” *Acta Derm. Venereol.*, vol. 103, p. adv00886, Mar. 2023, doi: 10.2340/actadv.v103.3491.

[24] A. W. Armstrong, C. T. Harskamp, and E. J. Armstrong, “Psoriasis and the risk of diabetes mellitus: a systematic review and meta-analysis,” *JAMA dermatology*, vol. 149, no. 1, pp. 84–91, Jan. 2013, doi: 10.1001/2013.jamadermatol.406.

[25] M. T. Wan, D. B. Shin, R. A. Hubbard, M. H. Noe, N. N. Mehta, and J. M. Gelfand, “Psoriasis and the risk of diabetes: A prospective population-based cohort study.,” *J. Am. Acad. Dermatol.*, vol. 78, no. 2, pp. 315-322.e1, Feb. 2018, doi: 10.1016/j.jaad.2017.10.050.

[26] M. Mamizadeh, Z. Tardeh, and M. Azami, “The association between psoriasis and diabetes mellitus: A systematic review and meta-analysis,” *Diabetes Metab. Syndr.*, vol.

For peer review only

For peer review only - http://bmjopen.bmj.com/site/about/guidelines.xhtml

13, no. 2, pp. 1405–1412, 2019, doi: 10.1016/j.dsx.2019.01.009.

[27] A. L. Neimann, D. B. Shin, X. Wang, D. J. Margolis, A. B. Troxel, and J. M. Gelfand, “Prevalence of cardiovascular risk factors in patients with psoriasis,” *J. Am. Acad. Dermatol.*, vol. 55, no. 5, pp. 829–835, Nov. 2006, doi: 10.1016/j.jaad.2006.08.040.

[28] L. Yang, L. Zhang, Q. Du, X. Gong, and J. Tian, “Exploring the molecular mechanism underlying the psoriasis and T2D by using microarray data analysis,” *Sci. Rep.*, vol. 13, no. 1, p. 19313, Nov. 2023, doi: 10.1038/s41598-023-46795-5.

[29] B. Saud *et al.*, “Fungal infection among diabetic and nondiabetic individuals in Nepal,” *Interdiscip. Perspect. Infect. Dis.*, vol. 2020, p. 7949868, 2020, doi: 10.1155/2020/7949868.

[30] S.-P. Sinikumpu *et al.*, “Abnormal skin in toe webs is a marker for abnormal glucose metabolism. A cross-sectional survey among 1,849 adults in Finland,” *Sci. Rep.*, vol. 7, no. 1, p. 9125, Aug. 2017, doi: 10.1038/s41598-017-09354-3.

[31] J. Spoendlin, J. J. Voegel, S. S. Jick, and C. R. Meier, “Risk of rosacea in patients with diabetes using insulin or oral antidiabetic drugs,” *J. Invest. Dermatol.*, vol. 133, no. 12, pp. 2790–2793, Dec. 2013, doi: 10.1038/jid.2013.225.

[32] S. Chahal, K. Vohra, and A. Syngle, “Association of sudomotor function with peripheral artery disease in type 2 diabetes,” *Neurol. Sci. Off. J. Ital. Neurol. Soc. Ital. Soc. Clin. Neurophysiol.*, vol. 38, no. 1, pp. 151–156, Jan. 2017, doi: 10.1007/s10072-016-2742-3.

[33] W. B. Horton and E. J. Barrett, “Microvascular dysfunction in diabetes mellitus and cardiometabolic disease,” *Endocr. Rev.*, vol. 42, no. 1, pp. 29–55, Jan. 2021, doi: 10.1210/endrev/bnaa025.

[34] Y. Liu *et al.*, “Factors influencing sweat gland innervation in diabetes,” *Neurology*, vol. 84, no. 16, pp. 1652–1659, Apr. 2015, doi: 10.1212/WNL.0000000000001488.

[35] G. P. Kenny, R. J. Sigal, and R. McGinn, “Body temperature regulation in diabetes,” *Temp. (Austin, Tex.)*, vol. 3, no. 1, pp. 119–145, 2016, doi: 10.1080/23328940.2015.1131506.

[36] B. Liu, X. Cheng, M. Chen, W. Dong, J. Sun, and Y. Lun, “Changes of sweat gland

For peer review only

For peer review only - http://bmjopen.bmj.com/site/about/guidelines.xhtml

function in type 2 diabetes mellitus patients with peripheral neuropathy," *Int. J. Diabetes Dev. Ctries.*, vol. 44, no. 1, pp. 84–90, 2024.

Figure legends

Figure 1 Flow chart demonstrating the process used to select the study participants.

Figure 2 Results of variable selection methods

Figure 3 The path analysis was performed in 1906 individuals aged 46 years. All depicted arrows represent statistically significant associations (Beta coefficients). Model fit values: SRMR = 0.031, RMSEA = 0.040.

Figure S1 Flow chart demonstrating the analytical plan

For peer review only

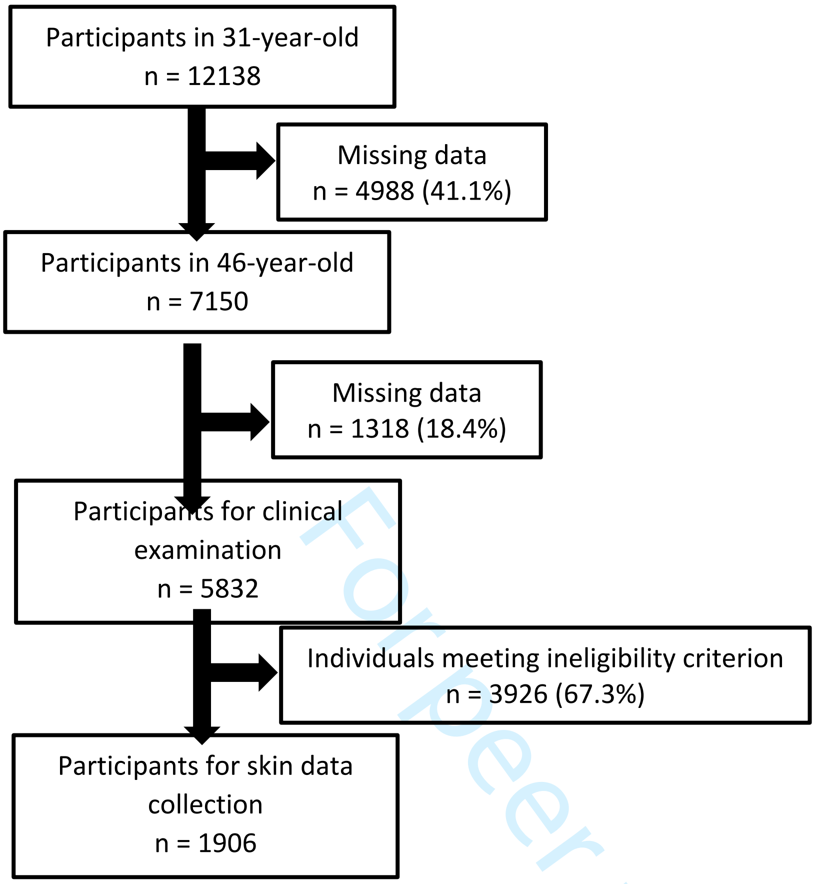

Figure 1 Flow chart demonstrating the process used to select the study participants.

For peer review only

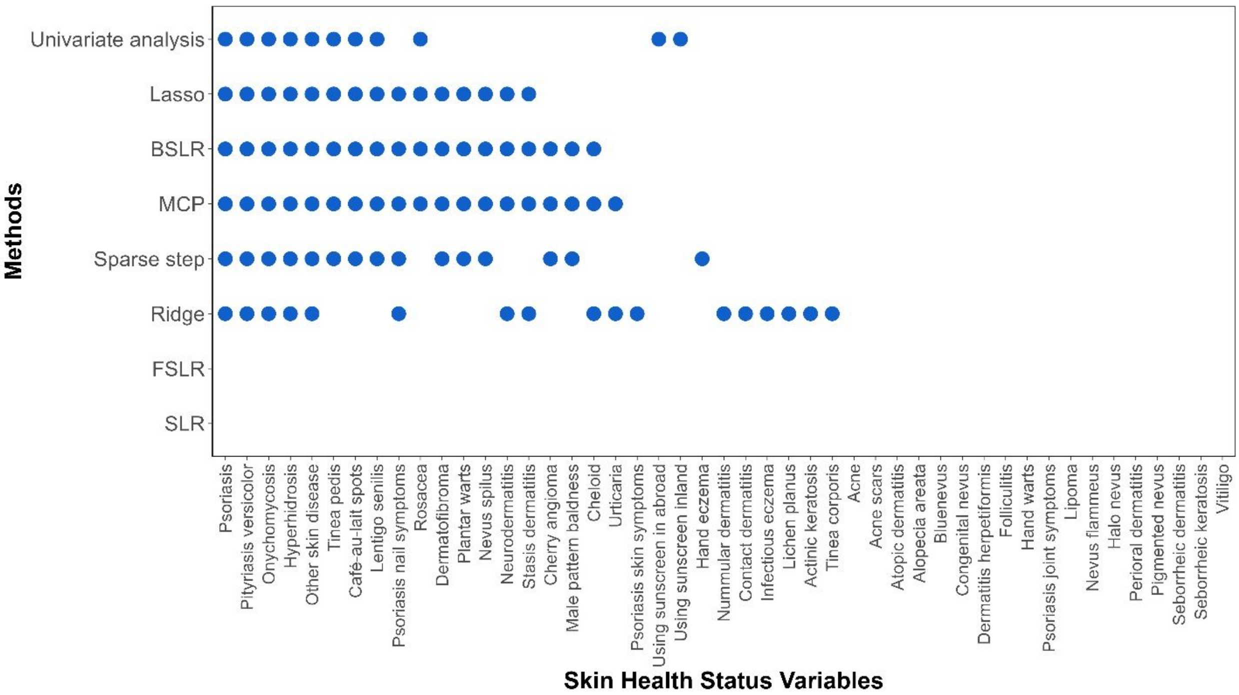

Figure 2 Results of variable selection methods

For peer review only

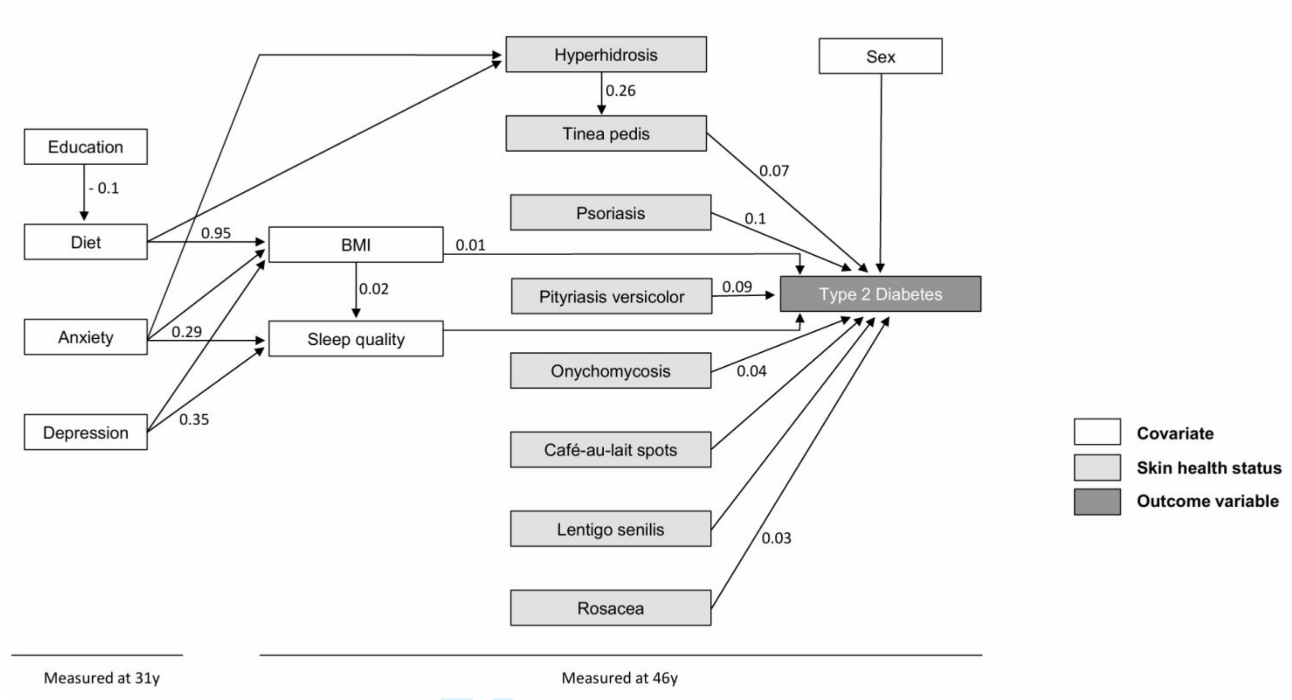

Figure 3 The path analysis was performed in 1906 individuals aged 46 years. All depicted arrows represent statistically significant associations (Beta coefficients). Model fit values: SRMR = 0.031, RMSEA = 0.040.

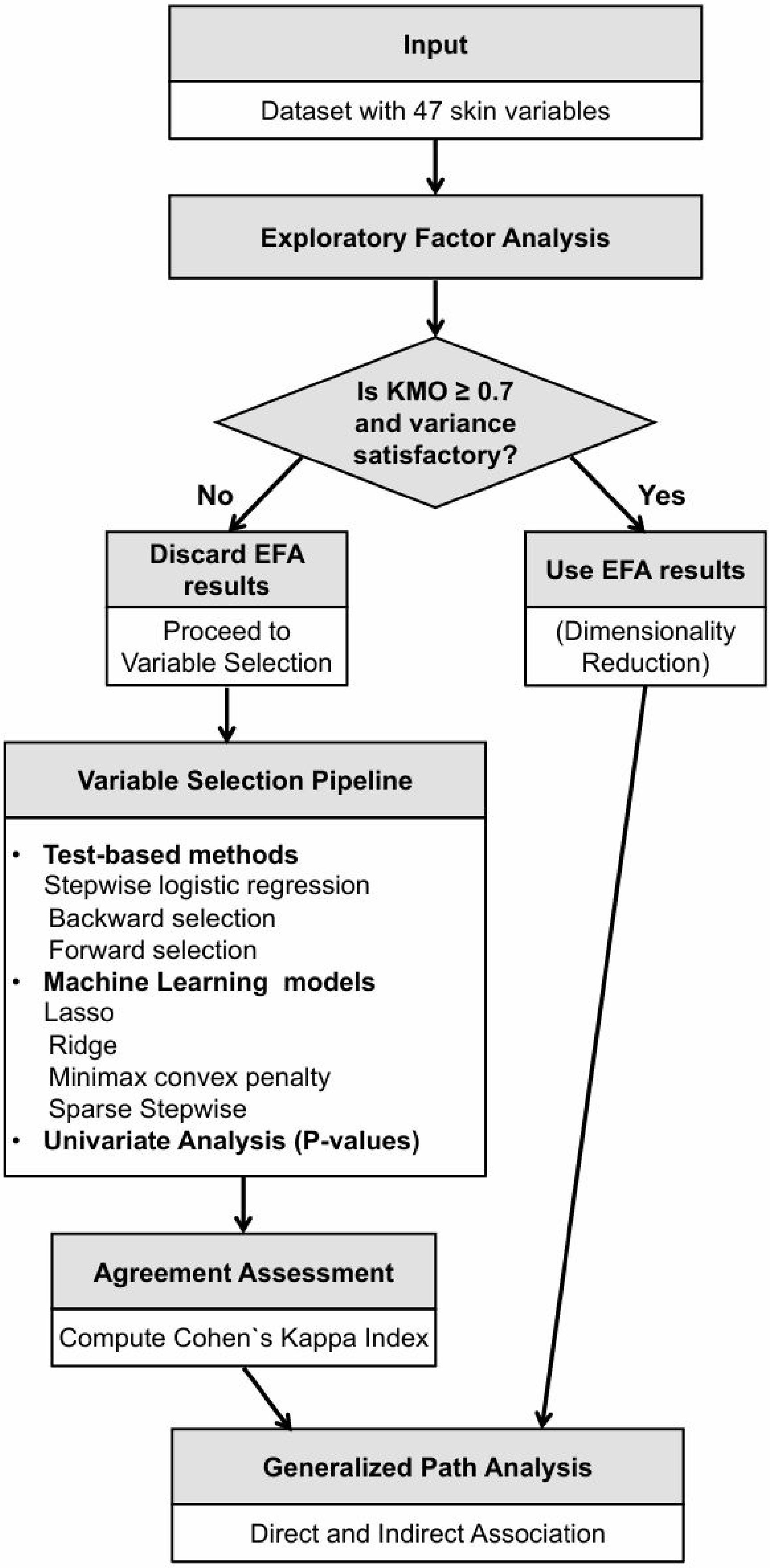

Figure S1 Flow chart demonstrating the analytical plan

Table S1 Descriptive characteristics of the study population

Variable	Category	Overall	Missing	Men	Women	P-value[a]	Healthy	Type 2 diabetes	P-value[b]
Tinea pedis	No	1392 (73.0 %)	0 (0 %)	557 (63.2 %)	835 (81.5 %)	<0.001	1316 (74.0 %)	55 (55.6 %)	<0.001
	Yes	514 (27.0 %)		325 (36.8 %)	189 (18.5 %)		463 (26.0 %)	44 (44.4 %)	
Hyperhidrosis	No	1864 (97.8 %)	0 (0 %)	850 (96.4 %)	1014 (99.0 %)	<0.001	1744 (98.0 %)	92 (92.9 %)	0.003
	Yes	42 (2.2 %)		32 (3.6 %)	10 (1.0 %)		35 (2.0 %)	7 (7.1 %)	
Café-au-lait spots	No	1668 (87.5 %)	0 (0 %)	776 (88.0 %)	892 (87.1 %)	0.614	1550 (87.1 %)	96 (97.0 %)	0.006
	Yes	238 (12.5 %)		106 (12.0 %)	132 (12.9 %)		229 (12.9 %)	3 (3.0 %)	
Pigmented nevus	No	1685 (88.4 %)	0 (0 %)	766 (86.8 %)	919 (89.7 %)	0.058	1572 (88.4 %)	88 (88.9 %)	1
	Yes	221 (11.6 %)		116 (13.2 %)	105 (10.3 %)		207 (11.6 %)	11 (11.1 %)	
Lentigo senilis	No	1650 (86.6 %)	0 (0 %)	794 (90.0 %)	856 (83.6 %)	<0.001	1531 (86.1 %)	94 (94.9 %)	0.018
	Yes	256 (13.4 %)		88 (10.0 %)	168 (16.4 %)		248 (13.9 %)	5 (5.1 %)	
Psoriasis	No	1783 (97.5 %)	78 (4.04 %)	820 (97.4 %)	963 (97.7 %)	0.815	1686 (97.8 %)	91 (92.9 %)	0.006
	Yes	45 (2.5 %)		22 (2.6 %)	23 (2.3 %)		38 (2.2 %)	7 (7.1 %)	
Psoriasis skin symptoms	No	1862 (97.9 %)	4 (0.21 %)	856 (97.4 %)	1006 (98.3 %)	0.198	1741 (98.0 %)	93 (94.9 %)	0.084
	Yes	40 (2.1 %)		23 (2.6 %)	17 (1.7 %)		35 (2.0 %)	5 (5.1 %)	
Psoriasis nail symptoms	No	1889 (99.4 %)	5 (0.26 %)	869 (98.9 %)	1020 (99.8 %)	0.022	1763 (99.3 %)	98 (100.0 %)	0.868
	Yes	12 (0.6 %)		10 (1.1 %)	2 (0.2 %)		12 (0.7 %)	0 (0.0 %)	
Psoriasis joint symptoms	No	1888 (99.3 %)	5 (0.26 %)	874 (99.4 %)	1014 (99.2 %)	0.775	1763 (99.3 %)	97 (99.0 %)	1
	Yes	13 (0.7 %)		5 (0.6 %)	8 (0.8 %)		12 (0.7 %)	1 (1.0 %)	
Rosacea	No	1614 (84.8 %)	2 (0.1 %)	790 (89.6 %)	824 (80.6 %)	<0.001	1513 (85.1 %)	76 (76.8 %)	0.035
	Yes	290 (15.2 %)		92 (10.4 %)	198 (19.4 %)		264 (14.9 %)	23 (23.2 %)	
Stasis dermatitis	No	1890 (99.2 %)	1 (0.05 %)	874 (99.1 %)	1016 (99.3 %)	0.773	1765 (99.3 %)	97 (98.0 %)	0.411
	Yes	15 (0.8 %)		8 (0.9 %)	7 (0.7 %)		13 (0.7 %)	2 (2.0 %)	
Bluenevus	No	1880 (98.6 %)	0 (0 %)	874 (99.1 %)	1006 (98.2 %)	0.162	1755 (98.7 %)	97 (98.0 %)	0.909
	Yes	26 (1.4 %)		8 (0.9 %)	18 (1.8 %)		24 (1.3 %)	2 (2.0 %)	
Nevus spilus	No	1817 (95.3 %)	0 (0 %)	843 (95.6 %)	974 (95.1 %)	0.714	1691 (95.1 %)	98 (99.0 %)	0.121
	Yes	89 (4.7 %)		39 (4.4 %)	50 (4.9 %)		88 (4.9 %)	1 (1.0 %)	
Halo nevus	No	1891 (99.2 %)	0 (0 %)	876 (99.3 %)	1015 (99.1 %)	0.819	1764 (99.2 %)	99 (100.0 %)	0.736
	Yes	15 (0.8 %)		6 (0.7 %)	9 (0.9 %)		15 (0.8 %)	0 (0.0 %)	
Dermatofibroma	No	1481 (77.7 %)	0 (0 %)	730 (82.8 %)	751 (73.3 %)	<0.001	1390 (78.1 %)	71 (71.7 %)	0.17
	Yes	425 (22.3 %)		152 (17.2 %)	273 (26.7 %)		389 (21.9 %)	28 (28.3 %)	
Cherry angioma	No	759 (39.8 %)	1 (0.05 %)	410 (46.5 %)	349 (34.1 %)	<0.001	705 (39.7 %)	44 (44.4 %)	0.4
	Yes	1146 (60.2 %)		472 (53.5 %)	674 (65.9 %)		1073 (60.3 %)	55 (55.6 %)	

For peer review only

Cheloid	No	1857 (97.4 %)	0 (0 %)	859 (97.4 %)	998 (97.5 %)	1	1735 (97.5 %)	95 (96.0 %)	0.526
	Yes	49 (2.6 %)		23 (2.6 %)	26 (2.5 %)		44 (2.5 %)	4 (4.0 %)	
Seborrheic keratosis	No	1061 (55.7 %)	0 (0 %)	522 (59.2 %)	539 (52.6 %)	0.005	987 (55.5 %)	58 (58.6 %)	0.616
	Yes	845 (44.3 %)		360 (40.8 %)	485 (47.4 %)		792 (44.5 %)	41 (41.4 %)	
Lipoma	No	1845 (96.8 %)	0 (0 %)	841 (95.4 %)	1004 (98.0 %)	0.001	1726 (97.0 %)	94 (94.9 %)	0.389
	Yes	61 (3.2 %)		41 (4.6 %)	20 (2.0 %)		53 (3.0 %)	5 (5.1 %)	
Congenital nevus	No	1828 (95.9 %)	0 (0 %)	838 (95.0)	990 (96.7 %)	0.086	1703 (95.7 %)	98 (99.0 %)	0.183
	Yes	78 (4.1 %)		44 (5.0 %)	34 (3.3 %)		76 (4.3 %)	1 (1.0 %)	
Nevus flammeus	No	1419 (74.4 %)	0 (0 %)	690 (78.2 %)	729 (71.2 %)	0.001	1316 (74.0 %)	79 79.8 %)	0.241
	Yes	487 (25.6 %)		192 (21.8 %)	295 (28.8 %)		463 (26.0 %)	20 (20.2 %)	
Actinic keratosis	No	1894 (99.4 %)	0 (0 %)	875 (99.2 %)	1019 (99.5 %)	0.582	1767 (99.3 %)	99 (100.0 %)	0.864
	Yes	12 (0.6 %)		7 (0.8 %)	5 (0.5 %)		12 (0.7 %)	0 (0.0 %)	
Atopic dermatitis	No	1813 (95.2 %)	1 (0.05 %)	847 (96.0 %)	966 (94.4 %)	0.128	1689 (95.0 %)	97 (98.0 %)	0.269
	Yes	92 (4.8 %)		35 (4.0 %)	57 (5.6 %)		89 (5.0 %)	2 (2.0 %)	
Hand eczema	No	1736 (91.1 %)	1 (0.05 %)	817 (92.6 %)	919 (89.8 %)	0.039	1614 (90.8 %)	95 (96.0 %)	0.115
	Yes	169 (8.9 %)		65 (7.4 %)	104 (10.2 %)		164 (9.2 %)	4 (4.0 %)	
Seborrheic dermatitis	No	1765 (92.7 %)	1 (0.05 %)	774 (87.8 %)	991 (96.9 %)	<0.001	1650 (92.8 %)	90 (90.9 %)	0.613
	Yes	140 (7.3 %)		108 (12.2 %)	32 (3.1 %)		128 (7.2 %)	9 (9.1 %)	
Nummular dermatitis	No	1869 (98.1 %)	1 (0.05 %)	858 (97.3 %)	1011 (98.8 %)	0.021	1746 (98.2 %)	96 (97.0 %)	0.618
	Yes	36 (1.9 %)		24 (2.7 %)	12 (1.2 %)		32 (1.8 %)	3 (3.0 %)	
Infectious eczema	No	1878 (98.6 %)	1 (0.05 %)	862 (97.7 %)	1016 (99.3 %)	0.007	1754 (98.7 %)	96 (97.0 %)	0.351
	Yes	27 (1.4 %)		20 (2.3 %)	7 (0.7 %)		24 (1.3 %)	3 (3.0 %)	
Contact dermatitis	No	1887 (99.1 %)	1 (0.05 %)	880 (99.8 %)	1007 (98.4 %)	0.006	1761 (99.0 %)	99 (100.0 %)	0.665
	Yes	18 (0.9 %)		2 (0.2 %)	16 (1.6 %)		17 (1.0 %)	0 (0.0 %)	
Neurodermatitis	No	1871 (98.2 %)	1 (0.05 %)	857 (97.2 %)	1014 (99.1 %)	0.002	1748 (98.3 %)	95 (96.0 %)	0.186
	Yes	34 (1.8 %)		25 (2.8 %)	9 (0.9 %)		30 (1.7 %)	4 (4.0 %)	
Lichen planus	No	1893 (99.3 %)	0 (0 %)	874 (99.1 %)	1019 (99.5 %)	0.407	1766 (99.3 %)	99 (100.0 %)	0.817
	Yes	13 (0.7 %)		8 (0.9 %)	5 (0.5 %)		13 (0.7 %)	0 (0.0 %)	
Dermatitis herpetiformis	No	1900 (99.7 %)	0 (0 %)	880 (99.8 %)	1020 (99.6 %)	0.821	1773 (99.7 %)	99 (100.0 %)	1
	Yes	6 (0.3 %)		2 (0.2 %)	4 (0.4 %)		6 (0.3 %)	0 (0.0 %)	
Vitiligo	No	1874 (98.3 %)	0 (0 %)	872 (98.9 %)	1002 (97.9 %)	0.123	1748 (98.3 %)	98 (99.0 %)	0.881
	Yes	32 (1.7 %)		10 (1.1 %)	22 (2.1 %)		31 (1.7 %)	1 (1.0 %)	
Urticaria	No	1896 (99.5 %)	0 (0 %)	877 (99.4 %)	1019 (99.5 %)	1	1770 (99.5 %)	98 (99.0 %)	1
	Yes	10 (0.5 %)		5 (0.6 %)	5 (0.5 %)		9 (0.5 %)	1 (1.0 %)	

For peer review only

Acne	No	1755 (92.1 %)	1 (0.05 %)	818 (92.7 %)	937 (91.6 %)	0.399	1635 (92.0 %)	94 (94.9 %)	0.377
	Yes	150 (7.9 %)		64 (7.3 %)	86 (8.4 %)		143 (8.0 %)	5 (5.1 %)	
Perioral dermatitis	No	1888 (99.3 %)	4 (0.21 %)	881 (99.9 %)	1007 (98.7 %)	0.007	1762 (99.3 %)	98 (99.0 %)	1
	Yes	14 (0.7 %)		1 (0.1 %)	13 (1.3 %)		13 (0.7 %)	1 (1.0 %)	
Acne scars	No	1716 (90.2 %)	4 (0.21 %)	780 (88.5 %)	936 (91.7 %)	0.026	1602 (90.2 %)	89 (90.8 %)	0.981
	Yes	186 (9.8 %)		101 (11.5 %)	85 (8.3 %)		174 (9.8 %)	9 (9.2 %)	
Pityriasis versicolor	No	1869 (98.1 %)	0 (0 %)	864 (98.0 %)	1005 (98.1 %)	0.9	1747 (98.2 %)	94 (94.9 %)	0.058
	Yes	37 (1.9 %)		18 (2.0 %)	19 (1.9 %)		32 (1.8 %)	5 (5.1 %)	
Onychomycosis	No	1726 (90.6 %)	0 (0 %)	757 (85.8 %)	969 (94.6 %)	<0.001	1619 (91.0 %)	83 (83.8 %)	0.027
	Yes	180 (9.4 %)		125 (14.2 %)	55 (5.4 %)		160 (9.0 %)	16 (16.2 %)	
Tinea corporis	No	1898 (99.6 %)	0 (0 %)	878 (99.5 %)	1020 (99.6 %)	1	1772 (99.6 %)	98 (99.0 %)	0.901
	Yes	8 (0.4 %)		4 (0.5 %)	4 (0.4 %)		7 (0.4 %)	1 (1.0 %)	
Folliculitis	No	1790 (93.9 %)	0 (0 %)	796 (90.2 %)	994 (97.1 %)	<0.001	1667 (93.7 %)	95 (96.0 %)	0.488
	Yes	116 (6.1 %)		86 (9.8 %)	30 (2.9 %)		112 (6.3 %)	4 (4.0 %)	
Plantar warts	No	1732 (90.9 %)	0 (0 %)	809 (91.7 %)	923 (90.1 %)	0.263	1613 (90.7 %)	94 (94.9 %)	0.207
	Yes	174 (9.1 %)		73 (8.3 %)	101 (9.9 %)		166 (9.3 %)	5 (5.1 %)	
Hand warts	No	1855 (97.3 %)	0 (0 %)	860 (97.5 %)	995 (97.2 %)	0.754	1731 (97.3 %)	96 (97.0 %)	1
	Yes	51 (2.7 %)		22 (2.5 %)	29 (2.8 %)		48 (2.7 %)	3 (3.0 %)	
Alopecia areata	No	1899 (99.6 %)	0 (0 %)	877 (99.4 %)	1022 (99.8 %)	0.338	1773 (99.7 %)	99 (100.0 %)	1
	Yes	7 (0.4 %)		5 (0.6 %)	2 (0.2 %)		6 (0.3 %)	0 (0.0 %)	
Male pattern baldness	No	1291 (67.7 %)	0 (0 %)	280 (31.7 %)	1011 (98.7 %)	<0.001	1202 (67.6 %)	69 (69.7 %)	0.741
	Yes	615 (32.3 %)		602 (68.3 %)	13 (1.3 %)		577 (32.4 %)	30 (30.3 %)	
Other skin disease	No	1595 (90.2 %)	0 (0 %)	753 (91.2 %)	842 (89.4 %)	0.453	1497 (90.6 %)	74 (82.2 %)	0.021
	Yes	168 (88.4 %)		71 (8.6 %)	97 (10.3 %)		150 (9.1 %)	16 (17.8 %)	
	Perhaps	5 (11.6 %)		2 (0.2 %)	3 (0.3 %)		5 (0.3 %)	0 (0.0 %)	
Using sunscreen inland	Not at all	260 (14.1 %)	60 (3.11 %)	190 (22.3 %)	70 (7.0 %)	<0.001	241 (13.9 %)	18 (18.4 %)	0.093
	Sometimes	890 (48.2 %)		458 (53.7 %)	432 (43.5 %)		838 (48.2 %)	47 (48.0 %)	
	Reqularly	614 (33.3 %)		171 (20.0 %)	443 (44.6 %)		587 (33.7 %)	25 (25.5 %)	
	I do not spend time in the sun	82 (4.4 %)		34 (4.0 %)	48 (4.8 %)		74 (4.3 %)	8 (8.2 %)	
Using sunscreen in abroad	Not at all	230 (13.1 %)	151 (7.82 %)	147 (18.3 %)	83 (8.7 %)	<0.001	216 (13.0 %)	13 (14.4 %)	0.658
	Sometimes	147 (8.4 %)		93 (11.6 %)	54 (5.7 %)		139 (8.4 %)	7 (7.8 %)	
	Reqularly	1172 (66.8 %)		472 (58.8 %)	700 (73.5 %)		1111 (67.0 %)	56 (62.2 %)	
	I do not spend time in the sun	206 (11.7 %)		91 (11.3 %)	115 (12.1 %)		192 (11.6 %)	14 (15.6 %)	

P-value[a] for differences between male and female. P-value[b] for differences between people with diabetes and healthy people.

For peer review only

Table S2 Distribution of study covariates at 31-year who participated in the 46-year follow-up and those who did not

Variable	Participated in the 46y	Not participated	P-value
**Sex**			<0.001
Male	3280 (46 %)	2935 (58 %)	
Female	3835 (54 %)	2086 (42 %)	
**Education, N (%)**			<0.001
<9 years	306 (5.5%)	223 (11.1%)	
Comprehensive school	4359 (78.4%)	1553 (77.2%)	
Highly educated	898 (16.1%)	235 (11.7%)	
**Diet, N (%)**			<0.001
Unhealthy	5522 (88.8%)	1929 (85.0%)	
Healthy	694 (11.2%)	341 (15.0%)	
**Anxiety, Mean (sd)**	1.304 (0.299)	1.339 (0.342)	<0.001
**Depression, Mean (sd)**	1.345 (0.345)	1.382 (0.377)	<0.001

For peer review only

Table S3 Distribution of study covariates at 31-year and 46-year follow-ups

Variable	31-year follow-up	46-year follow-up	P-value
**Education, N (%)**			<0.001
<9 years	72 (4.71 %)	38 (2.08 %)	
Comprehensive school	1221 (79.91 %)	1310 (71.70 %)	
Highly educated	235 (15.38 %)	479 (26.22 %)	
Missing, N	378	79	
**Diet, N (%)**			<0.001
Unhealthy	1518 (88.51 %)	1603 (93.90 %)	
Healthy	197 (11.49 %)	401 (6.10 %)	
Missing, N	191	199	
**Anxiety, Mean (sd)**	1.29 (0.28)	1.29 (0.30)	0.8113
Missing, N	183	112	
**Depression, Mean (sd)**	1.32 (0.32)	1.35 (0.37)	0.0019
Missing, N	203	112	

For peer review only

Table S4 Agreement coefficient between different methods

Method 1	Method 2	Cohen kappa (95% CI)	P_value
Ridge	Lasso	0.24 (-0.043, 0.53)	0.094
Ridge	Univariate analysis	0.16 (-0.122, 0.448)	0.248
Ridge	BSLR	0.23 (-0.058, 0.511)	0.12
Ridge	MCP	0.28 (0.002, 0.56)	0.053
Ridge	Sparse step	0.05 (-0.233, 0.342)	0.708
Lasso	Univariate analysis	0.53 (0.27, 0.799)	<0.001
Lasso	BSLR	0.86 (0.709, 1)	<0.001
Lasso	MCP	0.82 (0.649, 0.986)	<0.001
Lasso	Sparse step	0.71 (0.488, 0.924)	<0.001
Univariate analysis	BSLR	0.42 (0.159, 0.687)	0.002
Univariate analysis	MCP	0.39 (0.128, 0.651)	0.005
Univariate analysis	Sparse step	0.43 (0.151, 0.711)	0.003
BSLR	MCP	0.96 (0.869, 1)	<0.001
BSLR	Sparse step	0.77 (0.577, 0.958)	<0.001
MCP	Sparse step	0.73 (0.524, 0.927)	<0.001

For peer review only

Table S5 Variance inflation factor for selected variables

Variable	VIF
BMI	1.08
Diet	1.04
Poor Quality of sleep	1.06
Anxiety	2.38
Depression	2.38
Education	1.06
Psoriasis	1.03
Pityriasis versicolor	1.02
Tinea pedis	1.16
Onychomycosis	1.12
Café_au_lait_spots	1.01
Lentigo senilis	1.01
Rosacea	1.04
Hyperhidrosis	1.05

For peer review only

Table S6 Exponentiated Path coefficient analysis

Independent Variable	Total Effect	Direct Effect	Indirect Effect
Sex	1.004 (0.982, 1.026)	1.004 (0.982, 1.026)	-
BMI	1.011 (1.008, 1.014)	1.011 (1.008, 1.014)	1 (1, 1.001)
Diet	1 (1, 1.001)	-	1 (1, 1.001)
Sleep quality	0.995 (0.984, 1.005)	0.995 (0.984, 1.005)	-
Anxiety	0.999 (0.992, 1.018)	-	0.999 (0.992, 1.018)
Depression	0.998 (0.998, 1.003)	-	0.998 (0.998, 1.003)
Education	1 (1, 1)	-	1 (1, 1)
Psoriasis	1.105 (1.058, 1.156)	1.105 (1.058, 1.156)	-
Pityriasis versicolor	1.091 (1.038, 1.147)	1.091 (1.038, 1.147)	-
Tinea pedis	1.067 (1.04, 1.096)	1.067 (1.04, 1.096)	-
Onychomycosis	1.042 (1.01, 1.074)	1.042 (1.01, 1.074)	-
Café-au-lait spots	0.958 (0.904, 1.015)	0.958 (0.904, 1.015)	-
Lentigo senilis	0.966 (0.923, 1.011)	0.966 (0.923, 1.011)	-
Rosacea	1.03 (1.004, 1.058)	1.03 (1.004, 1.058)	-
Hyperhidrosis	1.017 (1.005, 1.037)	-	1.017 (1.005, 1.037)

For peer review only
